# A Label-Free Proteomic and Complementary Metabolomic Analysis of Leaves of the Resurrection Plant *Xerophyta* *schlechteri* during Dehydration

**DOI:** 10.3390/life11111242

**Published:** 2021-11-16

**Authors:** Hawwa Gabier, David L. Tabb, Jill M. Farrant, Mohamed Suhail Rafudeen

**Affiliations:** 1 Department of Molecular and Cell Biology, University of Cape Town, Private Bag X3, Rondebosch 7701, South Africa; hawwa.gabier@alumni.uct.ac.za (H.G.); jill.farrant@uct.ac.za (J.M.F.); 2 Institute of Infectious Disease and Molecular Medicine, Faculty of Health Sciences, University of Cape Town, Cape Town 7700, South Africa; dtabb@sun.ac.za; 3 Division of Molecular Biology and Human Genetics, Faculty of Medicine and Health Sciences, Stellenbosch University, Cape Town 7500, South Africa; 4 Centre for Bioinformatics and Computational Biology, Stellenbosch University, Stellenbosch 7602, South Africa

**Keywords:** dehydration, desiccation tolerance, plant proteomics, label-free quantification, metabolomics, resurrection plant, *Xerophyta schlechteri*

## Abstract

Vegetative desiccation tolerance, or the ability to survive the loss of ~95% relative water content (RWC), is rare in angiosperms, with these being commonly called resurrection plants. It is a complex multigenic and multi-factorial trait, with its understanding requiring a comprehensive systems biology approach. The aim of the current study was to conduct a label-free proteomic analysis of leaves of the resurrection plant *Xerophyta schlechteri* in response to desiccation. A targeted metabolomics approach was validated and correlated to the proteomics, contributing the missing link in studies on this species. Three physiological stages were identified: an early response to drying, during which the leaf tissues declined from full turgor to a RWC of ~80–70%, a mid-response in which the RWC declined to 40% and a late response where the tissues declined to 10% RWC. We identified 517 distinct proteins that were differentially expressed, of which 253 proteins were upregulated and 264 were downregulated in response to the three drying stages. Metabolomics analyses, which included monitoring the levels of a selection of phytohormones, amino acids, sugars, sugar alcohols, fatty acids and organic acids in response to dehydration, correlated with some of the proteomic differences, giving insight into the biological processes apparently involved in desiccation tolerance in this species.

## 1. Introduction

Most higher plants cannot withstand severe water loss, except for a small group of angiosperms called resurrection plants. Resurrection plants are phylogenetically diverse and exhibit contrasting anatomy, drying and rehydration kinetics but typically co-occur in specific habitat types in which there are frequent periods of extended drought [1]. They can survive extreme water loss (ca 95% of total cellular water) without the loss of viability by employing numerous complex mechanisms that facilitate desiccation tolerance (DT). It has been proposed that for plant tissue to survive severe water loss (desiccation), three criteria should be met [2]. First, damage to the tissue should be limited, and it should be at an extent that is repairable. Secondly, the plant should be able to maintain its physiological integrity in the dried state. Lastly, the plant must be able to initiate repair and restoration of metabolism and growth upon rehydration in tissues affected by desiccation. All these criteria are variously met in resurrection plants and several comprehensive reviews on the mechanisms, and variations exist among species [3,4,5,6,7,8,9,10,11].

*Xerophyta schlechteri* (Baker) N.L. Menezes has become a model plant for studying the mechanisms of vegetative desiccation tolerance for the ultimate production of drought-tolerant cereals [12]. Considerable anatomical, ultrastructural and physiological studies have detailed the whole plant and cellular responses to desiccation and recovery there it [13,14,15], and the genomic, transcriptomic and metabolic changes that facilitate the tolerance for extreme water loss in this species have been documented [16,17,18]. Similar to other resurrection plants, *X. schlechteri* also activates antioxidant systems [5,17] and produces HSPs and LEAs [13,19], as well as certain sugars [18] purported to be core protection mechanisms in vegetative desiccation tolerance. As of yet, no label-free proteomic studies have been reported on this species, which could serve to correlate and verify observed transcriptome or metabolome changes. Furthermore, there have been few studies on phytohormones (other than ABA) in resurrection plants. Thus, the aim of the present study was to utilize a label-free proteomics approach in combination with metabolomics to elucidate the key biological processes and their potential regulation during dehydration and desiccation of *X. schlechteri*.

Label-free quantification (LFQ) utilizes several techniques for the quantification and identification of differentially expressed proteins in complex biological samples. LFQ techniques have been mainly applied to study biotic stress responses [20,21,22,23,24], and there are only a few reports on using such techniques in abiotic stresses. The approach is strengthened with downstream bioinformatics techniques where Blast2GO and MapMan are commonly used. In our study design, protein expressions were further validated using metabolomics techniques. The nature of many primary metabolites accumulated in resurrection plants contributes significantly to their ultimate survival and the induction of metabolic quiescence upon reaching the air-dry state [9,25,26]. An approach where proteomics is complemented by metabolomics allows further decoding of the key biochemical pathways and biological processes associated with desiccation tolerance in this species. This in turn could be used to improve drought tolerance in crop plants. The aim of this study was to use the LFQ proteomics approach complemented with metabolomics for the identification and validation of relevant biological processes and pathways involved in establishing desiccation tolerance in *X. schlechteri*.

## 2. Materials and Methods

### 2.1. Plant Material, Growth Conditions and Dehydration Treatments

*X. schlechteri* was collected from the Buffelskloof Nature Reserve near Lydenberg (Mpumalanga Province, South Africa). The plants were maintained under glasshouse conditions at the University of Cape Town (South Africa) until required for the dehydration experiments. Five mature plants of a similar size were then acclimated for 2 weeks in an environmental chamber (Conviron Adaptis A350, Canada) with the following controlled environmental settings: 16 h of light with a 300 μmol m^−2^ s^−1^ light intensity at 25 °C; 8 h in the dark at 20 °C; and 50% relative humidity. Senescent leaf tips [18] were removed at the start of acclimation. Dehydration was achieved by withholding water, and leaves were sampled at a specified time daily for determination of relative water content (RWC). Leaf tissue was also harvested for proteomic and metabolomics studies and immediately frozen in liquid nitrogen and stored at −80°C until further processing.

The classic method of determination of a plant’s RWC, as outlined in [27], was followed with one exception. As the leaf tissues of *X. schlechteri* do not absorb water during the required overnight incubation, the turgid weight cannot be easily and accurately calculated. Thus, in this study, the absolute water content (AWC) was gravimetrically determined and calculated as AWC = (FW − DW)/DW (=gH2O g^−1^ dwt), where FW is the tissue mass on excision from the plant and DW is the mass after oven drying of that tissue for 48 h at 70 °C. The RWC was determined utilizing the AWC of leaves at full turgor (AWCFT) prior to the initiation of dehydration, and it was expressed as follows:

AWC/AWCFT × 100.

### 2.2. Label-Free Proteomic Analyses and Workflow

The proteomic analysis workflow consisted of wet laboratory, raw data processing and bioinformatics components. The proteins extracted from *X. schlechteri* (3 replicates from 5 different plants for each RWC stage) were subjected to LC-MS/MS analyses, and the raw data was processed to obtain protein identification and the statistically significant differential expression of proteins during dehydration. These differentially expressed proteins were then functionally annotated using Blast2GO and Mercator pipelines for GO enrichment and pathway analyses.

#### 2.2.1. Protein Extraction, Sample Solubilization and Quantification

Frozen leaf material was ground in a chilled mortar and pestle to a fine powder with liquid nitrogen and 1% (*w*/*w*) insoluble polyvinylpolypyrrolidone (PVP). Protein was extracted and separated from the DNA and RNA using Tri-reagent (Sigma-Aldrich Corporation, St. Louis, MO, USA) according to the manufacturer’s instructions. Cold 100% ethanol was added to the organic phase containing the total protein, inverted at room temperature for 5 min and then centrifuged for 15 min at 2500× *g*. The supernatants containing soluble proteins were transferred to 2-mL low-bind Eppendorf tubes containing 1.5 mL isopropanol. The tubes were incubated for 10 min at room temperature and centrifuged for 15 min at 10,250× *g* at 4 °C. The resultant protein pellet was washed with 0.1 M ammonium acetate (prepared in 100% methanol) and then cold acetone. Thereafter, the washed pellets were air dried and stored at −80 °C until further use. For solubilization, the protein pellets were resuspended in 50 mM triethylammonium bicarbonate (TEAB; Sigma, USA, T7408) and 2% sodium dodecyl sulphate (SDS; Sigma, USA, 71736) and placed at 95 °C for 5 min to solubilize. The samples were centrifuged at 13,000× *g* for 5 min. Quantification was performed using the QuantiPro BCA assay kit (Sigma, USA, QPBCA) according to the manufacturer’s instructions.

#### 2.2.2. On-Bead Hydrophilic Interaction Liquid Chromatography (HILIC) and Trypsin Digestion

HILIC magnetic beads (ReSyn Biosciences, HLC010) were aliquoted into a fresh tube, and the carrier solution was removed. The beads were washed with a 250-μL wash buffer (15% ACN, 100 mM ammonium acetate (Sigma, USA, 14267); pH 4.5) for 1 min and repeated. The beads were resuspended in a loading buffer (30% ACN, 200 mM ammonium acetate; pH 4.5). The rest of the process described hereafter was performed using a Hamilton MassSTAR robotics liquid handler (Hamilton, Switzerland). A total of 50 μg of protein from each sample was transferred to a protein LoBind plate (Merck, USA, 0030504.100). The protein was reduced with tris (2-carboxyethyl) phosphine (TCEP; Sigma, USA 646547), which was added to a final concentration of 10 mM TCEP and incubated at 60 °C for 1 h. The samples were cooled to room temperature, alkylated with methylmethanethiosulphonate (MMTS; Sigma, USA, 208795) (final concentration of 10 mM MMTS) and incubated at room temperature for 15 min. HILIC magnetic beads were added at an equal volume to that of the sample with a ratio of 5:1 for the total protein. The plate was then incubated on a shaker for 30 min at room temperature for binding of the protein to the beads to take place. After binding, the beads were washed with 500 μL of 95% ACN. The protein was digested by incubation with trypsin (Promega V5111, Santa Barbara, CA, USA) for 4 h on a shaker at 37 °C. After digestion, the peptides were removed, dried and then resuspended in an LC loading buffer (0.1% FA and 2.5% ACN).

#### 2.2.3. Liquid Chromatography–Mass Spectrometry (LC-MS)

The LC-MS analysis was conducted with a Q-Exactive quadrupole-Orbitrap mass spectrometer (Thermo Fisher Scientific, Waltham, MA, USA) coupled with a Dionex Ultimate 3000 nano-HPLC system (Thermo Fisher Scientific, USA). The peptides were dissolved in 2% ACN and 0.1% FA (Sigma, USA, 56302), (Burdick & Jackson, USA, BJLC015CS) and loaded on a C18 trap column (PepMap100, 300 μm × 5 mm × 5 μm). Samples containing peptides were trapped onto the column and washed for 3 min, after which the valve was switched and the peptides were eluted onto the analytical column. The chromatographic separation was further performed using a Waters nanoEase (Zenfit, Copenhagen, Denmark) M/Z Peptide CSH C18 column (75 μm × 25 cm × 1.7 μm) with a two-solvent system (Solvent A (1% FA) and B (1% FA in ACN)). The multi-step gradient for peptide separation was generated at 300 nL as follows: time change: 5 min; gradient change: 2–5% solvent B; time change: 40 min; gradient change: 5–18% solvent B; time change: 10 min; gradient change: 18–30% solvent B; time change: 2 min; gradient change: 30–80% solvent B. The gradient was then held at 80% solvent B for 10 min before returning it to 2% solvent B and conditioning the column for 15 min. The mass spectrometer was operated with a capillary temperature of 320 °C and operated in positive ion mode. The applied electrospray voltage was 1.95 kV using Proxeon stainless steel emitters (Thermo Fisher, USA, TFES523).

#### 2.2.4. Database Identification of Peptides and Proteins

The LC-MS/MS raw data (spectra) output was converted to mzML format. The MS-GF+ (v2018.10.15) search engine [28], which scores MS/MS spectra against peptides derived from a protein sequence database, was used to determine the potential peptides. The search was performed with semi-tryptic specificity for the 52 *X. schlechteri* mzML files and searched against the *X. schlechteri* database (version 3).

The downloaded protein FASTA file (Xvis3_proteins.fa) contained 25,425 sequences with description strings. The MS-GF+ database search default settings were standard amino acids with fixed C+46, a precursor mass tolerance of 10 ppm and limited sets of modifications for methionine oxidation and cysteine beta-methylthiolation. The mzid identification files were processed using IDPICKER v3.1 [29] to yield a 2% peptide spectrum match (PSM) FDR and required two distinct peptide sequences for each protein. A 4.44% empirical FDR was obtained with 2889 distinguishable protein groups matching 19,222 distinct peptide sequences and 152,912 peptide spectrum matches (spectra). DESeq2 v1.4.5 in R-Bioconductor [30] was used to analyze the data, detect differentially expressed proteins, normalize the counts and test for the significant differences between the desiccation states according to their abundance in the samples using the Wald test and a *p*-value of 0.05.

### 2.3. Gene Ontology Analysis Pipeline

#### 2.3.1. Protein Annotation and GO-Term Retrieval

The protein identifications were further enriched by adding functional ontological information using Blast2GO v.5.2 [31] based on the biological process, molecular function and cellular components. Investigation of the proteins was conducted using the *X. schlechteri* FASTA database file (August 2018) searched against the UNIPROTKB/SwissProt database using the BLASTP algorithm. The blast parameters were at their default settings (GO weight of 5, *e*-value filter of 1 × 10^−6^, an hsp-hit coverage cut-off of 0 and an annotation cut-off of 55).

#### 2.3.2. Mercator and MapMan Analysis and Workflow

Mercator v.3.6 [32] is an online tool that assigns functional annotations to protein sequences using the MapMan “BIN” ontology [33]. The *X. schlechteri* input text file was uploaded to Mercator, which assigned each input identifier using protein domain searches or BLAST-based searches to one or more BINs based on the significant similarity to the reference databases. The output file was then directly used as a mapping file for further high-throughput visualization using MapMan software [33].

MapMan (v.3.6) software was used for the visualization of the differentially expressed *X. schlechteri* proteins with the “Scavenger” and “ImageAnnotator” modules [34]. The Scavenger module creates a non-redundant gene ontology, while the ImageAnnotator module allows for the visualization of differentially expressed data. The MapMan software was configured to display the *X. schlechteri* upregulated proteins and downregulated proteins across the dehydration treatments based on a Log2 fold change with a *p*-value ≤0.05 indicating statistical significance using the Wilcoxon rank-sum test.

### 2.4. Metabolomics Workflow

#### 2.4.1. Gas Chromatography–Mass Spectrometry (GC-MS) Analysis

Extraction and derivatization of the metabolites was conducted as described previously [35]. The library of standards used in this study was prepared in the same manner, using a mixture of amino acids, sugars, phytohormones, organic acids and fatty acids (amino acids: alanine, 5-oxoproline, L-serine, aspartic acid, serine, L-histidine, glycine, L-phenylalanine, valine, proline, norleucine, tyrosine, methionine, asparagine, lysine and tryptophan; sugars: galactose, fructose, glucose, erythritol, raffinose, sucrose and cellobiose; sugar alcohols: myo-inositol, sorbitol, mannitol and arabitol; organic acids: malic acid, citric acid and lactic acid; and fatty acids: quininic acid, palmitic acid and stearic acid).

The experiments were performed on an Agilent 7000C gas chromatograph equipped with a 7693 auto sampler and interfaced to a 7000A Triple Quadrupole mass spectrometer (Agilent Technologies, Santa Clara, CA, USA). MassHunter B.05 GC MS/MS software (Agilent, Waldbronn, Germany) was used to identify and quantify the metabolites from the spectrum using the acquired spectrum data, identifying the metabolites using the known standards given above and the internal standard (ribitol).

#### 2.4.2. LC/MS Metabolite Profiling of Phytohormones

The phytohormones analyzed were gibberellic acid (GA), Jasmonyl-l-94 isoleucine (JA lle), salicylic acid (SA), indole-3-carboxylic acid (ICA), jasmonate (JA), indole-3-acetic acid (IAA-Asp), abscisic acid (ABA) and indole-3-butyric acid (IBA). These were run individually on an AB SCIEX QTRAP 4000 LC-MSMS system coupled to an ACQUITY UPLC (Waters, Milford, UK). The samples were analyzed in positive ionization mode, followed by the same samples being analyzed in negative ionization mode.

#### 2.4.3. Data Processing

The chromatogram data obtained from both the GC MS/MS and LCMS was analyzed in Openchrom 1.3 [Available online: https://www.openchrom.net/download, accessed on 24 January 2020] with Savitsky–Golay smoothing, using the AMDIS and NIST MS (https://chemdata.nist.gov/mass-spc/ms-search/, accessed on 24 January 2020) libraries to identify specific metabolites. MetaboAnalyst version 4 (https://www.metaboanalyst.ca/, accessed on 24 January 2020) [36] performed comparative statistics using ANOVA with Fisher’s PLS-DA method and a *t*-test to compare the relative abundance across samples, taking into account variances in the dataset [37]. These results were further validated by performing heatmap hierarchical clusters based on the difference in the respective metabolites across the drying stages. The hierarchical cluster was represented as a heatmap with dendrograms using the Euclidean distance.

## 3. Results

### 3.1. Physiological Characterization

**Figure 1:**
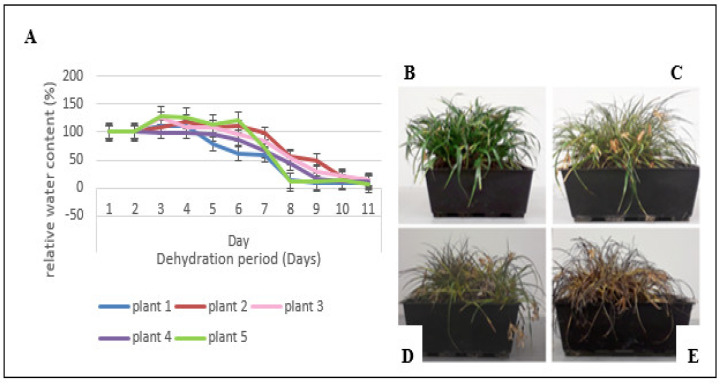
(**A**) Changes in relative water content of leaves of *X. schlechteri* under dehydration stress over a period of 11 days. Dehydration was performed on five biological replicates (*n* = 5). (**B**) *X. schlechteri* representing hydrated (control) (~100% relative water content, ~2.1 gH2O g^−1^ dwt). (**C**) *X. schlechteri* represented at the early response to drying (~80–70% relative water content, from ~1.61 gH_2_O g^−1^ dwt to ~1.5 gH_2_O g^−1^ dwt) (**D**) *X. schlechteri* at mid-response to drying (~60–40% relative water content, from ~1.5 gH_2_O g^−1^ dwt to ~1.0 gH_2_O g^−1^ dwt). (**E**) *X. schlechteri* represented at the late response to drying (~40–10% relative water content, from ~1.0 gH_2_O g^−1^ dwt to ~ 0.5 gH_2_O g^−1^ dwt).

Under the conditions used in the current study, the plants took 8–10 days (with individual variation) to reach an air-dry state (Figure 1). Three phases of dehydration could be visualized. There was an early response to dehydration (ERD) occurring over the first 6–8 days, in which the RWC declined from full turgor to ~80–70% RWC (from ~1.61 gH_2_O g^−1^ dwt to ~1.5 gH_2_O g^−1^ dwt), during which the leaves maintained their green color (Figure 1C). This was followed by a mid-response to dehydration (MRD) of 2–3 days, during which the RWC further declined to between ~60–40% RWC (from ~1.5 gH_2_O g^−1^ dwt to ~1.0 gH_2_O g^−1^ dwt), and the leaves started losing their green color (Figure 1D). During the late response to dehydration (LRD) (~40–10% RWC; from ~1.0 gH_2_O g^−1^ dwt to ~0.5 gH_2_O g^−1^ dwt), the leaves were folded such that only the abaxial surfaces were exposed to light, with these becoming anthocyanin rich as seen by the dark shade of purple (Figure 1E).

### 3.2. Proteomic Analysis

The statistical design of the R package DESeq 2 analyses was based on identifying the differentially expressed proteins and their differential expression across different treatments (Supplementary Tables S1–S3). The differential expression was expressed as a Log2 fold change for the *p*-values (≤0.05) and was complemented with *X. schlechteri* accession, protein, InterPro and GO-term information. A total of 3125 unique proteins were identified in the *X. schlechteri* leaf material across the treatments (early, mid and late), of which a combined 517 proteins were differentially expressed in response to drying. Among the differentially expressed proteins, 253 proteins were upregulated, and 264 proteins were downregulated. At ERD (Supplementary Table S1), 29 proteins were found to be upregulated, while 33 were downregulated. For MRD (Supplementary Table S2), 56 proteins were upregulated, and 128 were downregulated, while LRD (Supplementary Table S3) had 168 proteins that were upregulated and 103 proteins that were downregulated. There were 20 *X. schlechteri* proteins that were differentially regulated across all three dehydration stages, while most differentially regulated proteins were only identified in a specific dehydration stage or across two stages (Supplementary Tables S4 and S5). There were 65 proteins that were identified as proteins of unknown function (Supplementary Table S6).

The proteomic data gave a myriad of protein identifications which required annotation, biological context and visualization in order to understand the key processes and networks underlying the drying responses of *X*. *schlechteri.* In order to gain this understanding, differentially expressed proteins were compared across the three stages of drying (including the up- and downregulated proteins) using gene ontology tools such as Blast2GO and MapMan.

#### 3.2.1. Blast2GO Analyses

**Figure 2:**
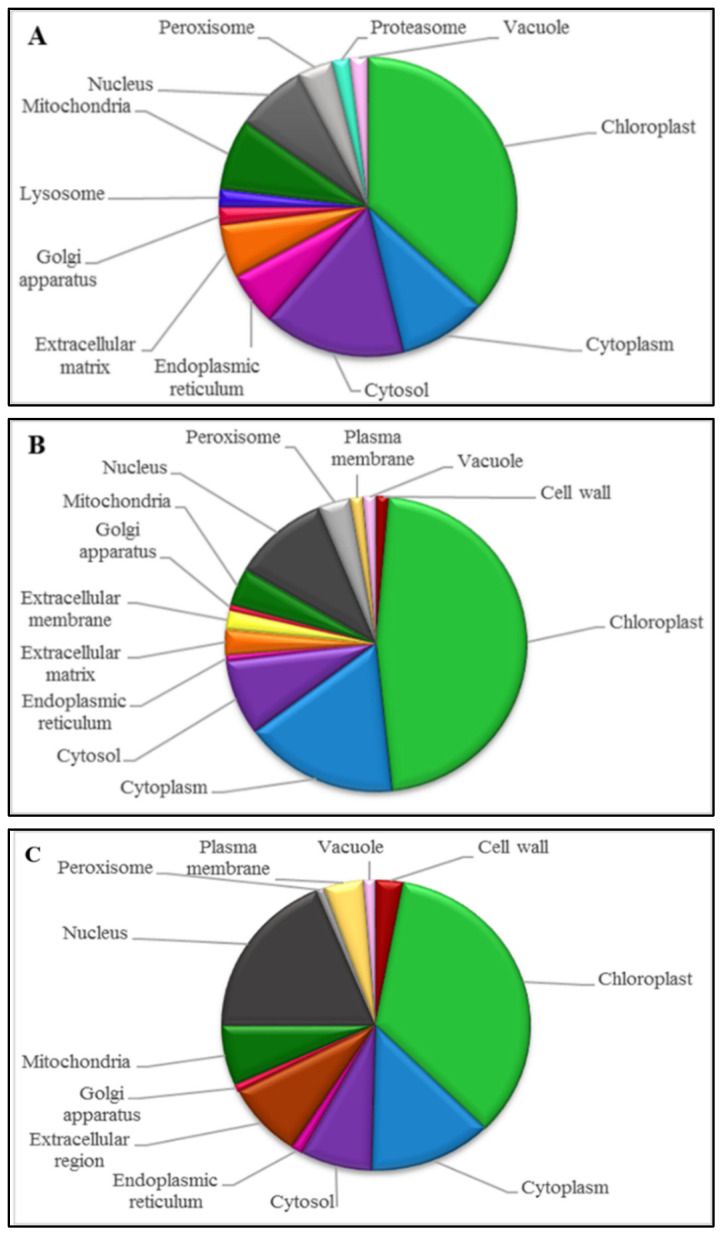
Representation of cellular component prediction of the identified *X. schlechteri* differentially expressed proteins based on GO-annotation at the (**A**) early response to drying, (**B**) mid-response to drying and (**C**) late response to drying. The proportion of each cellular component can be compared to early response to drying which was as follows: chloroplast (36%), cytosol (15%), cytoplasm (9%), nucleus (8%), mitochondria (8%), endoplasmic reticulum (6%), extracellular matrix (6%), peroxisome (4%), Golgi apparatus (2%), lysosome (2%), vacuole (2%) and proteasome (2%) respectively.

**Figure 3:**
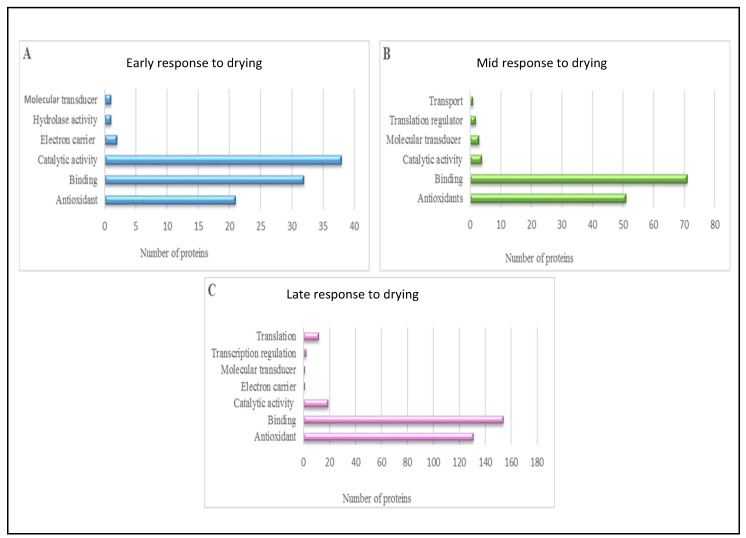
Molecular process predictions of the identified *X. schlechteri* differentially expressed proteins based on GO-annotation. (**A**) Early response to drying. (**B**) Mid-response to drying. (**C**) Late response to drying.

**Figure 4:**
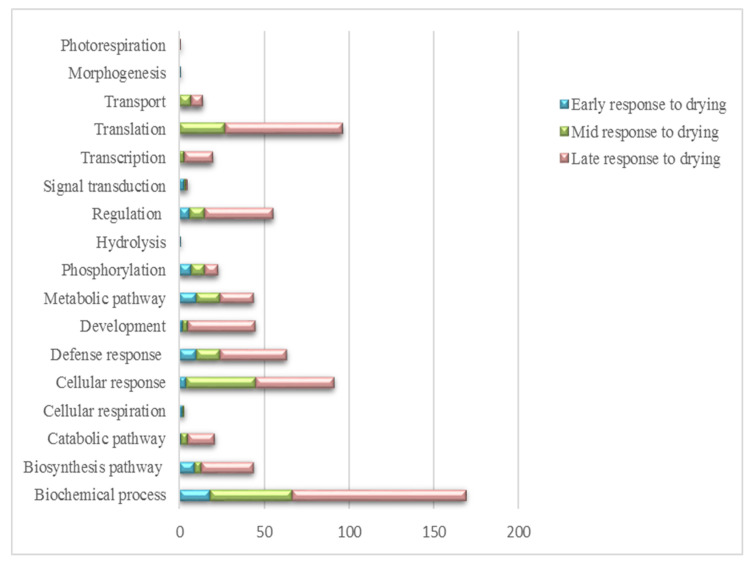
Biological process predictions of *X. schlechteri* differentially expressed proteins across treatments.

The Blast2GO program allows proteins to be assigned to three non-overlapping ontologies, namely cellular component, molecular function and biological process (Figure 2, Figure 3 and Figure 4, respectively). The respective ontologies and proteins assigned to each process were examined according to the ERD, MRD and late LRD responses to dehydration and desiccation.

Unsurprisingly, the most highly represented cellular components during the ERD (Figure 2A) included those from the chloroplasts (36%) and the cytosol (15%). Other cellular components that were observed were the cytoplasm (9%), nucleus (8%), mitochondria (8%), endoplasmic reticulum (6%), extracellular matrix (6%), peroxisome (4%), golgi apparatus (2%), lysosome (2%), vacuole (2%) and proteasome. At the MRD (Figure 2B) as well as for the LRD (Figure 2C), similar cellular compartments were observed. However, there was an increase in the chloroplasts (47%) at the MRD, while for the LRD, there was a decrease in this organelle (35%). This is in line with the poikilochlorophyllous nature of the species. Furthermore, at the MRD, the cytoplasm (17%) and the nucleus (10%) increased in relative expression, while the cytosol (8%) decreased compared with the ERD. The cell wall (1%) and plasma membrane (1%) were represented at the MRD and LRD and not at the ERD, suggesting increased modifications of these organs with dehydration and desiccation, as has been shown to occur in this and other *Xerophyta* species [38,39].

The GO-terms allocated to the molecular functions of *X. schlechteri* revealed that the ERD was mostly associated with antioxidants (4%), binding (6%) and catalytic activity (15%) (Figure 3A). As the drying progressed and reached the MRD (Figure 3B), the proteins belonging to antioxidants (9%), binding activity (13%) and catalytic activity (3%) increased compared with the ERD. In addition, the proteins belonging to transport (1%) and translation regulation (3%) were observed at the MRD. A significant increase in the proteins belonging to binding (47%), antioxidants (24%) and catalytic activity (7%) was observed at the LRD (Figure 3C). Transcription (2%) and translation (5%) also became associated with the LRD, while proteins belonging to electron carriers and molecular transducers decreased compared with the ERD. What is striking about these results is the steady increase in binding and catalytic activity, particularly at the LRD of the antioxidants. This supports the well-known association between antioxidant protection and dehydration and desiccation in resurrection plants. Furthermore, this suggests that there was an increase chaperonin-like activity and favorable molecular associations with increased water loss, with an increase in the catalytic potential in such water-limited environments. Ongoing transcription [16,40] and translation [41,42] in the LRD is a phenomenon observed in resurrection plants, but it is not entirely understood.

The GO-terms allocated to the biological processes of *X. schlechteri* indicated that proteins belonging to biochemical processes were increased at the LRD when compared with the ERD and MRD (Figure 4). Similarly, this increase at the LRD could be seen for the biosynthesis pathway, catabolic pathways and cellular and defense response categories. The proteins belonging to translation increased at the MRD and continued through to the LRD. This again points to ongoing and possibly specific biochemical metabolisms at very low water contents (<40% RWC).

#### 3.2.2. MapMan Analyses

**Figure 5:**
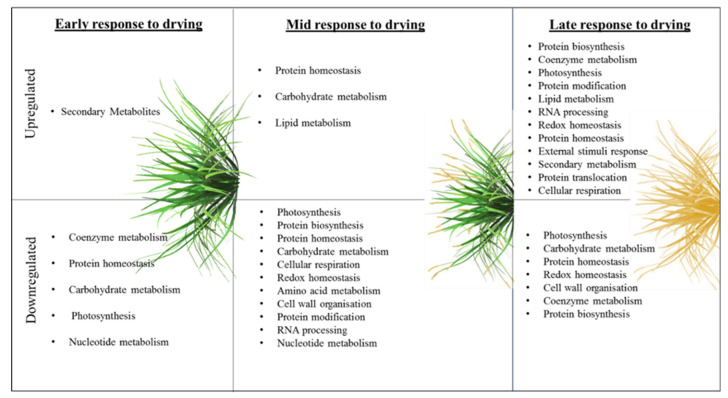
Visual representation of the *X. schlechteri* response to dehydration based on MapMan gene ontology. Gene ontologies associated with differentially expressed *X. schlechteri* proteins were mapped to BINs in MapMan (see Supplementary Tables S4–S9). The upregulated and downregulated proteins were mapped across the drying stages (early response to drying, mid-response to drying and late response to drying).

MapMan was also used to obtain the functional information of the dehydration and desiccation responsive proteins of *X. schlechteri,* where the differentially expressed proteins were assigned to various BINs based on MapMan ontology (Supplementary Tables S7–S9). A total of 253 proteins out of 269 were mapped to 25 functional BINs across the dehydration treatments. The tables display the BIN numbers, BIN names and number of elements, which were the proteins allocated to each BIN, along with the associated probabilities (*p*-value ≤ 0.05) with significance tested through the Wilcoxon rank-sum method. To obtain a holistic view of the response of *X. schlechteri* across the ERD, MRD and LRD, and based on the results from the MapMan gene ontology outline above, a visual representation of the responses is given in Figure 5.

**Figure 6:**
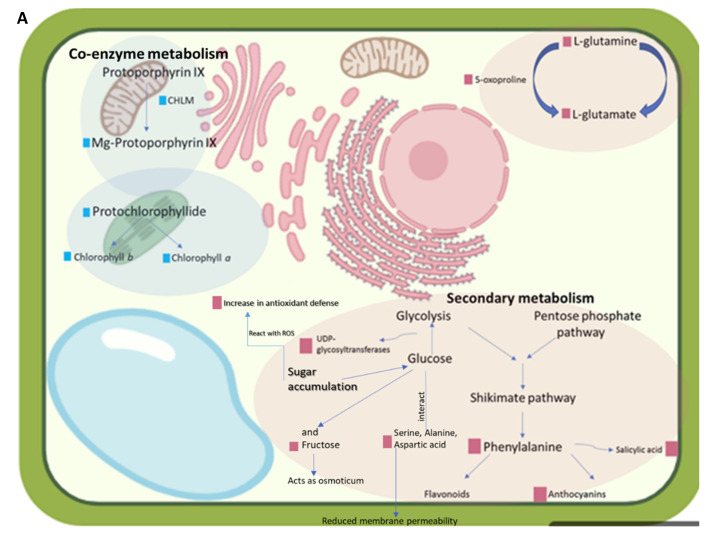

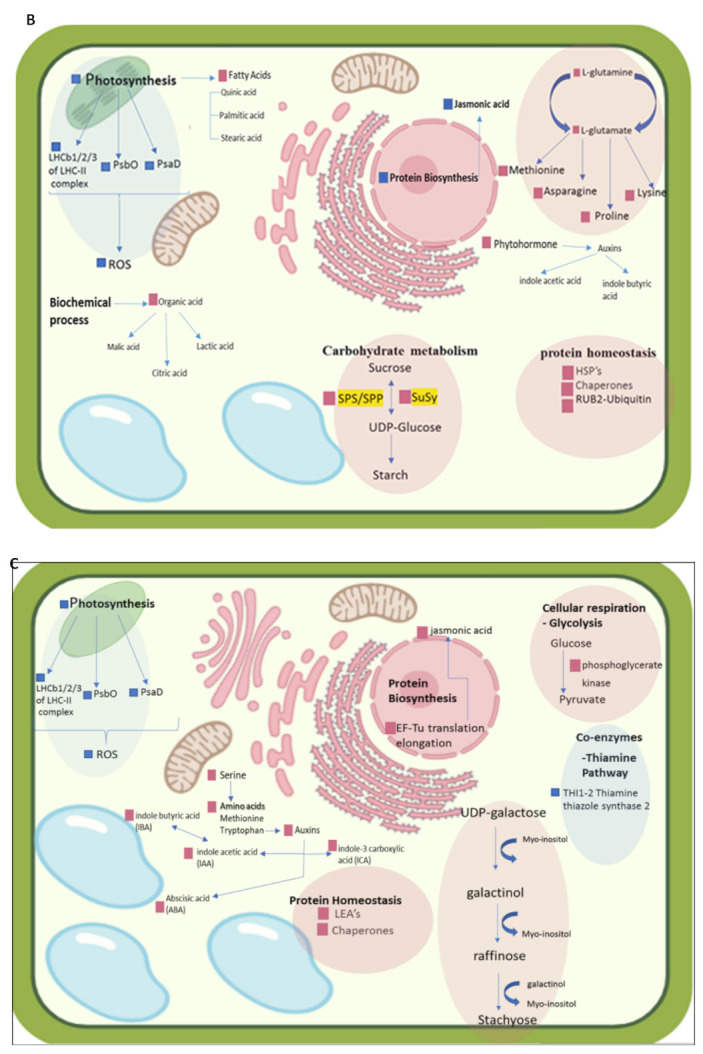
Proposed model of key responses of *X. schlechteri* at the (**A**) ERD, (**B**) MRD and (**C**) LRD. Pink squares represent upregulation, and blue squares represent downregulation.

While each stage of dehydration was associated with both up- and downregulation of various metabolic processes, the number of upregulated processes increased with dehydration and significantly so in the LRD where, despite the minimal water content, there appeared to be ongoing complex metabolism. The number of downregulated processes, presumably required for redirecting or shutting down unnecessary metabolic processes, increased in the MRD but declined considerably in the LRD. The overall trends that can be inferred from these changes, together with the metabolomics data discussed below, are modeled in Figure 6C.

### 3.3. Metabolomic Analysis

**Figure 7:**
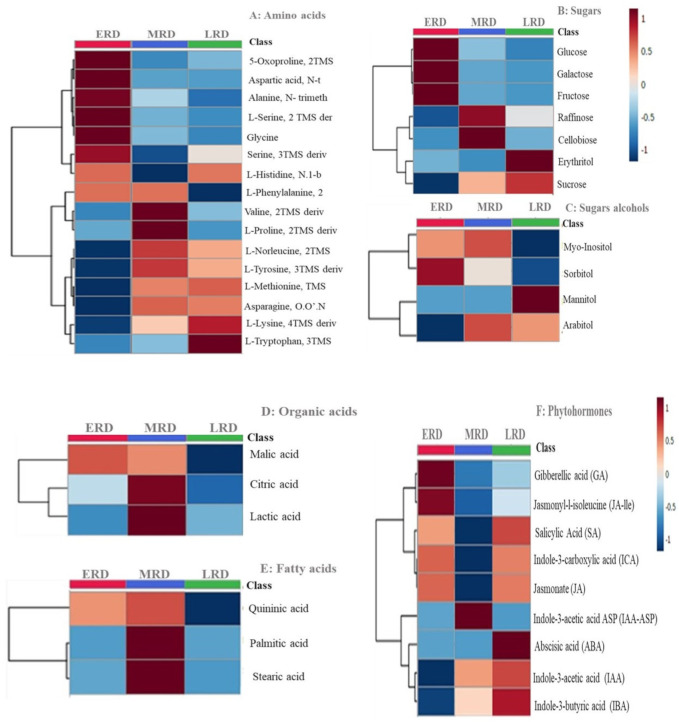
Heatmap of differentially abundant selected metabolites in *X. schlechteri*. The heatmap shows (**A**) amino acids across drying stages, (**B**) sugars across drying stages, (**C**) sugar alcohols across drying stages, (**D**) organic acids across drying stages, (**E**) fatty acids across drying stages and (**F**) phytohormones across drying stages. The “class” shown on the right side of the heatmap lists the metabolites. The drying stages represented are early response to drying (ERD), mid-response to drying (MRD) and late response to drying (LRD). Shades of maroon represent a high abundance of metabolites, and shades of blue represent a low abundance of metabolites.

Heatmap hierarchical clusters were used to illustrate the abundance of the different metabolites across the three drying stages in *X. schlechteri* leaf tissue (Figure 7). Although this study used a targeted approach in part for validation of the proteomic studies (discussed below), some meaningful observations can be drawn in terms of the metabolic responses of *X. schlechteri* to desiccation. Among the amino acids, there was an early increase in alanine, 5-oxoproline, L-serine, aspartic acid, glycine and serine, with these declining in the mid-to-late states of drying. These, together with monosaccharides (glucose, fructose and galactose) and some sugar alcohols (myoinositol and sorbitol) which were observed to increase at this stage, are likely to play an osmoregulatory or “priming” role, enabling some initial retention of water and preparation (possibly epigenetically) for subsequent further water loss [26,43,44]. During the MRD, there was an increase in valine, proline, norleucine, tyrosine, methionine, asparagine and lysine, most of which (except proline and valine) continued to be present in elevated levels in the LRD. A large increase in tryptophan was evident in the LRD. However derived, these were likely to contribute toward subcellular stabilization and as stores for recovery of metabolism upon rehydration. Changes in the nature of the amino acids between early and mid-dehydration, as well as increased levels of different amino acids present in the mid-to-late stages of drying, have been reported for most angiosperm resurrection plants [18,42,44,45], with each species tending to accumulate their own specific mixes thereof [25,26]. The trend changes in the relative quantities of sugars, sugar alcohols and organic and fatty acids observed during the MRD and LRD were similar to those reported for other resurrection plants, whereas for the amino acids, different players are involved in different species [25,26].

With respect to the phytohormones analyzed, and in light of the paucity of reportage on such metabolites in angiosperm resurrection plants, some general observations are made here. The leaf tissues in the ERD had elevated levels of gibberellic acid (GA), jasmonate (JA), Jasmonyl-l-isoleucine (JA-ile), salicylic acid (SA) and indole-3 carboxylic acid (ICA), with the levels of all of these declining in the MRD and with JA, SA and ICA peaking again in the LRD. Given the proposed and varied roles of these phytohormones [46,47], these responses were likely linked to regulation of the associated processes of photosynthesis and metabolic defense against reactive oxygen species (ROS) and osmotic stress. The well-described auxins indole acetic acid (IAA) and indole butyric acid (IBA) peaked in the LRD. In the only other study on auxins in resurrection plants, it was shown that a similar trend occurs in *Craterostigma wilmsii*, with those authors attributing this to a requirement for root generation upon rehydration [10]. Abscisic acid (ABA), known for its role in vegetative desiccation tolerance (predominantly through transcriptome studies [7,16]), increased only in the LRD. Yobi et al. (2017) [48] reported the same trend in the resurrection grass *Spolobolus stapfianus*, although Vicre et al. (2004) showed that in the dicot resurrection plant *C. wilmsii*, ABA spikes in the ERD and declines relative to this in the MRD and LRD were present [10].

#### Validating and Correlating a Subset of Proteomic Data with Metabolomics Data

Figure 6 gives schematic diagrams summarizing and modeling the major correlative changes between the proteomes and metabolomes. In the ERD, the quantitative proteomic data correlated with the metabolomics data confirmed the abundance of respective proteins and sugars associated with secondary metabolism (Figure 6A and Figure 7, Supplementary Table S10). The proteomic data demonstrated a downregulation in co-enzyme metabolism, carbohydrate metabolism and photosynthesis (Supplementary Tables S11–S15). The decline in photosynthesis continued during the MRD (Figure 6B, Supplementary Table S13), coinciding with a decline in photosynthetic proteins psbO and psbP, which are components of the luminal oxygen evolving complex of PSII. Furthermore, the chlorophyll a/b binding protein and oxygen evolving complex proteins decreased in abundance, confirming the breakdown of the photosynthetic apparatus noted to occur at this stage in *X. schlechteri* [14,18]. The induction of carbohydrate metabolism in the MRD correlated with the accumulation of sucrose and the increased presence of the enzymes sucrose phosphate synthase (SPS) and sucrose synthase (SuSy). Both enzymes have been associated with sucrose accumulation in resurrection plants [49]. Upregulation of the proteins involved in protein homeostasis during the LRD (Figure 6C, Supplementary Table S14) correlates with the accumulation of ABA (Figure 7F), which binds to the transcription factors associated with protein biosynthesis to upregulate the expression of LEA proteins [50]. ABA is also known to regulate the accumulation of osmolytes, correlating with the increases in sucrose, sugar alcohols mannitol and arabitol and several amino acids (Figure 7).

## 4. Discussion

### 4.1. Physiological Characterization

During successive dehydration, leaves of *X. schlechteri* pass through three distinct phases, namely the ERD (~80–70% RWC), MRD (~60–40% RWC) and LRD (~40–10% RWC), which correlate with presence of and distinct changes in the proteins and metabolites identified. The phenotypical changes observed during these three stages (Figure 1) are typical of the poikilochlorophyllous nature of this monocotyledonous resurrection plant [14,15,18], and the observed changes were directly related to changes in the proteins (photosynthesis and cell wall regulation) and metabolites (anthocyanins) predicted to be present from proteomic and metabolomics studies.

### 4.2. Insights Gleaned from Proteomic and Metabolomic Analyses

As the proteome in many instances gives rise to the metabolome, the roles of the selected proteins in a biological process can be validated or supported by corresponding metabolomics data. The metabolomic profile is influenced by proteins through enzymatic activities, pathways and transport, while changes in protein expression may be affected by metabolite concentrations [51]. A combination of these “omic” approaches may thus provide a more comprehensive understanding of the biological function [52]. Using this approach, we speculated on the roles of the various proteins in and where possible metabolites facilitate desiccation tolerance in *X. schlechteri*.

#### 4.2.1. Early Response to Drying

During the ERD, several of the upregulated proteins were involved in secondary metabolism (Figure 5), correlating with increased levels of the precursors in terms of amino acids, sugars, sugar alcohols and phytohormones essential to their synthesis (Figure 7). Secondary metabolites are used inter alia as antioxidants and sunscreens (e.g., anthocyanins) for protection against stresses associated with water loss and UV light [5,53], and their upregulation at this early stage is likely to be associated with protection of the photosynthetic apparatus from ROS damage before and during its dismantling. The proteins involved in secondary metabolism (Supplementary Table S10), UDP-glycosyltransferase (GO: 0016758) and chloroplastic polyphenol oxidase (PPO) (GO: 0004097) are both involved in defense against droughts [54,55], and UDP glycosyltransferases have been linked to anthocyanin production [55]. Supporting this role in *X. schlechteri*, it was previously reported that anthocyanin production increased during this stage of dehydration, which continues throughout dehydration in this species [17]. UDP-glycosyltransferases belong to the glycosyltransferase family in plants and are responsible for controlling many metabolic processes and transferring sugar moieties onto small molecules [55]. It was reported that the overexpression of UDP-glycosyltransferase enhanced the tolerance to drought, low temperatures and salt stress in *Arabidopsis thaliana* [55]. PPO catalyzes the oxidation of monophenols or o-diphenols to o-quinones with the concomitant reduction of oxygen to water. Several roles for PPO have been proposed, including defense against herbivory, pathogens and abiotic stresses, particularly those affecting photosynthesis [54], although the exact role they might play remains equivocal and may vary among species. The spike in SA, itself an active secondary metabolite, in the ERD could have several functions, given the multiple roles of this phytohormone. However, in the context of dehydration, this is likely related to the ability of SA to stimulate antioxidant defense mechanisms [46]. The increase in JA and JA-ile, known signaling molecules involved in the biosynthesis of secondary metabolites [47], further supports the noted upregulation of this category in the ERD.

The early increases in several amino acids and monosaccharide sugars which subsequently declined in the MRD suggests their involvement in inter alia osmoregulation, priming and preparation for further metabolic shifts in the MRD. Marked increased levels of alanine in the ERD have been reported in *X. schlechteri* [18] and *S. stapfianus* [44], with all authors claiming a role in osmoprotection. This amino acid also plays a role in amino acid transport, ribosomal structure and biogenesis, translation, coenzyme transport and metabolism [56], suggesting other possible roles for its abundant presence. Increased levels of 5-oxoproline, a cyclic amino acid formed as a result of the dehydration of glutamate, is also of interest. It is an intermediate substrate involved in the synthesis of glutathione, an antioxidant shown to be vital to the survival of desiccation in resurrection plants [5] and involved in suppression of programmed cell death and senescence [57]. Furthermore, our protein data suggest involvement of the glutamate pathway in the ERD (Figure 6A). Upregulation of the glutamine synthetase (GS)/glutamine-oxoglutarate aminotransferase (GOGAT) cycle indicates its production [58,59]. Among its many functions, glutamate is involved in protein synthesis and is a major amino acid donor for the synthesis of nitrogen compounds and amino acids [60], which are found to accumulate during drying in *X. schlechteri*.

Our study also shows that certain proteins associated with metabolic processes, namely co-enzyme, carbohydrate and nucleotide metabolism, were downregulated at the ERD, as were proteins involved in protein homeostasis and photosynthesis (Figure 5). Co-enzyme metabolism accounted for the largest number of proteins allocated to these processes (Supplementary Table S11) and included HEMC porphobilinogen deaminase (GO: 0004568), CHLM magnesium protoporphyrin IX methyltransferase (GO: 0004149), PORB protochlorophyllide reductase B (GO: 0016630) and CHLP geranylgeranyl diphosphate reductase (GO: 0046406), all of which are involved in photosynthesis and chlorophyll synthesis. CHLP geranylgeranyl diphosphate reductase catalyzes the reduction of geranylgeranyl diphosphate to phytyl diphosphate, providing phytol for both tocopherol and chlorophyll synthesis [61]. Its downregulation thus implies declined synthesis of either or both of these molecules. Downregulation of chlorpohyll biosynthesis is consistent with the poikilochlorophyllous nature of *X. schlechteri*. Collett et al. (2003) reported a decline in transcription of CHLP geranylgeranyl diphosphate reductase in the poikilochlorophyllous sister species *Xerophyta humilis,* although this occurred only in the MRD at an RWC below 60% [62]. PORB protochlorophyllide reductase B was another co-enzyme that was downregulated at the ERD. This enzyme is involved in the chlorophyll biosynthesis pathway [63], and thus its downregulation, along with CHLP geranylgeranyl diphosphate reductase, is further in keeping with the poikilochlorophyllous nature of *X. schlechteri*.

Fewer proteins were associated with carbohydrate and nucleotide metabolism than with co-enzyme metabolism (Supplementary Table S11). Carbohydrate metabolism showed downregulation of 6-phosphogluconolactonase (GO: 0005975) and trans aldolase (GO: 0015979), which are enzymes that are involved in the pentose phosphate pathway (PPP). This pathway is central to plant metabolism in that it parallels or can serve as an alternative to glycolysis [64]. It has two distinct connected phases, namely an oxidative phase and a non-oxidative phase. The latter serves as a precursor for nucleotide biosynthesis [65], while the oxidative phase results in the conversion of hexose phosphates to glyceraldehyde-3-phosphate and CO_2_ and thus, via its role in photosynthesis, can in turn affect various aspects of plant growth, chloroplast development and stem elongation [66]. Modulation of its activity during the ERD in *X. schlechteri* could slow photosynthesis. This could explain the decline in photosynthetic assimilation observed to occur below 80% RWC in this species [14].

In line with this, the photosynthetic processes downregulated included Rieske iron-sulfur (GO: 0005515) and the PNSL1 photosynthetic NDH subunit of lumenal (GO: 0009765). Rieske iron-sulfur belongs to a complex through which electrons flow, allowing energy to be used by the carbon source of the plant, and it was also found to increase photosynthesis by 10% [67]. The PNSL1 photosynthetic NDH subunit of lumenal is a PsbP-like protein that is involved in photosystem II repair in *A. thaliana* [68]. Interestingly, Collett et al. (2003) [62] showed a decline in PsbP transcripts below 60% RWC in *X. humilis*, confirming the finding here.

One of the homeostasis processes downregulated involved HSP 90 (GO: 0003924), which plays an important role in stress response, plant development and disease resistance [69]. HSP 90 is the most abundant HSP with multiple proposed functions, and it has been shown to be accumulated during the LRD in *X. schlechteri*, where it has been proposed to predominantly play a chaperonin role [70]. The reason for its initial decline observed in this study may reflect that it is at the ERD, and *X. schlechteri* is in the initial stages of dehydration and does not require this protection.

Other proteins involved in protein homeostasis were RPN8A 26S proteasome non-ATPase regulatory subunit 7 homolog A (GO: 0004017), which plays a role in the determination of leaf polarity [71], and asparaginyl endopeptidase (GO: 0016630), which is reported to have a role in plant defense and seed storage protein maturation [72]. The functionality of downregulation of these can only be surmised, but changes in leaf polarity may be in preparation for the leaf folding observed during dehydration (Figure 1), and suppression of maturation of the seed storage proteins could infer a tempering in the production of certain LEA proteins. Costa et al. (2017) showed de novo synthesis of LEA transcripts from four different families, including the seed maturation proteins during the ERD in *X. schlechteri* [16].

#### 4.2.2. Mid-Response to Drying

As drying continued into the MRD, three processes were upregulated (Figure 5). Protein homeostasis predominantly involved HSP70 (heat shock cognate 70 kDa protein) (GO: 0006457) and RUB2 (ubiquitin-NEDD8-like protein RUB2) (GO: 0005515) (Supplementary Table S12). Protein homeostasis is the ability of the cell to properly manufacture, fold and deactivate protein molecules in response to both internal and external environments [73]. HSPs in general are believed to combat the damaging effects of protein denaturation [74], and HSP 70 in particular is known to provide cytoprotection against macromolecular damage [75]. It has been shown to accumulate during the MRD and LRD in *Xerophyta viscosa* [74]. Increased levels of RUB2 could relate to the observed phenomenon in this and other resurrection plants of activation of the ubiquitin proteasomal system (UPS), proposed to be a less severe response than autophagy and allowing for the release of nutrients for protective purposes [40,42,76].

Carbohydrate metabolism is crucial for cellular protection in resurrection plants [3]. Its upregulation in the MRD involved UDP-glucose pyrophosphorylase (GO: 0006011) and PHS2 alpha-glucan phosphorylase 2 (GO: 0005975). The former is a key enzyme in sucrose metabolism and carbohydrate biosynthesis [77]. It is thought to work in coordination with sucrolytic enzymes (namely SuSy, fructokinase and SPS/SPP) and hexose phosphates [78] to produce sucrose and other polysaccharides [79]. The sucrose, raffinose and cellobiose contents increased considerably in the MRD, and sucrose in particular increased in the LRD (Figure 7). While sugars have numerous proposed roles, in resurrection plants, the universal presence of high levels of sucrose, and in many instances accompanying raffinose family oligosaccharides, is believed to have a signaling and ultimately structural role in vitrification of the cytoplasm (reviewed in [9,80]). Increases in the sugar alcohols arabitol and myoinosital support a protective and signaling role. Arabitol is a known osmoprotectant under water deficit stress [81], and importantly, it contributes to the formation of arabinoglycans, which have been shown to play a significant role in wall flexibility under desiccation stress in *X. schlechteri* and other resurrection plants [8]. Myoinositol is important for phosphate storage, the production of stress-related molecules, cell-to-cell communication and the storage and transport of plant hormones, and it can alleviate ionic stresses [82].

Lipid metabolism was also upregulated at the MRD, which involved phospholipase A2 (GO: 0004623) and PLP1 patatin-like protein 1 (GO: 0006629). Phospholipase A2 proteins are involved in pathways which ultimately result in the production of JA and related compounds [83]. While the levels of jasmonates declined in the MRD, there was an increase in JA at the LRD (Figure 6), and the increase in phospholipase A2 might have been in preparation for this. Interestingly, all fatty acids tested in this work were significantly upregulated in the MRD (Figure 6).

Several amino acids accumulated in the MRD that differed from those accumulated in the ERD. Most continued to be elevated in the LRD (Figure 6), and these probably generally contributed to cytoplasmic stabilization via Natural Deep Eutectic Solvent (NaDES) formation and as nitrogen stores for recovery upon rehydration.

A large number of proteins were downregulated at the MRD (Figure 5). Among these, unsuprisingly, the largest number of proteins downregulated was for those associated with photosynthesis (Supplementary Table S13), particularly those proteins associated with the photosystem II complex (PSII), with this being in line with the dismantling of photosystems at this stage of dehydration [18].

Protein biosynthesis-related proteins were also downregulated, possibly suggesting a general slowing down of translation. These proteins included RPL5 50S ribosomal protein L5 (GO: 0016620), which is responsible for the synthesis of proteins involved in the photosynthetic apparatus. Plant serine-threonine proteins are known to regulate signal transduction pathways via phosphorylation [84,85]. Downregulation of the processes associated with carbohydrate metabolism and protein homeostasis is counterintuitive, given the simultaneous upregulation of aspects of these in the MRD. However, this together with the downregulation of the processes associated with cellular respiration, redox homeostasis, cell wall organization, amino acid metabolism, RNA processing, nucleotide metabolism and protein modification all point to a regulated readjustment and slowing of metabolism as the RWC declines below 60%. The decline in respiration was corroborated by an increase in the citrate in the MRD (Figure 7), and this, together with the elevated sucrose, could imply the initiation of some degree of NaDES formation, as suggested by Radermacher et al. (2019) [18]. This in turn could facilitate antioxidant activity and temper mitochondrial ROS formation [25].

#### 4.2.3. Late Response to Drying

The numerous upregulated processes in the LRD (Figure 5 and Supplementary Table S14) again point to a finely tuned regulation of metabolism, despite the low RWC in which this metabolism is occurring. Interestingly, the process with the highest number of proteins was protein biosynthesis, an ongoing process in the LRD for many resurrection plants [42,74,86,87]. These include the production of LEA proteins almost exclusively in the LRD, which inter alia are proposed to act as chaperonins and ion sequesters in the prevention of macromolecular aggregation, in facilitating liquid-liquid phase transitions (and thus possible regulation of translation) and, together with sugars, in vitrification and subcellular stabilization [9,88]. Such proteins are induced by ABA, which peaked at this stage (Figure 6). As mentioned elsewhere, ABA is essential to desiccation tolerance, and Farrant and Hilhorst (2021) proposed that this late peak in ABA content might trigger the ABI5 transcription factor, imposing a type of dormancy which enables greater longevity in the desiccated state [26]. Increased SA and JA in the LRD correlated with the upregulation of secondary metabolism and redox homeostasis, and the marked increase in the auxins ICA, IAA and IBA at this stage, in addition to being in preparation for stimulation of root growth upon rehydration, as proposed by Vicre et al. (2003) [10], could play a regulatory role in water availability in the dry and rehydrating states [89].

Protein modification (involving S-glutathionylation and protein folding) and protein translocation were also upregulated at the LRD. An aqueous environment is believed to be essential for correct protein folding, and here, one can only assume that this occurs in isolated pockets of remaining water or, as has been proposed by du Toit et al. (2020), in regions occupied by NaDES that facilitate ongoing metabolism at low water contents [25]. The accumulation of NaDES-forming metabolites (such as sucrose, citrate, malate and various amino acids (Figure 7)) supports this suggestion. Such an environment would also facilitate protein translocation and RNA processing (Supplementary Table S14) which, among other elements, involves several chaperone proteins. The upregulation of RNA processes could also involve the transcription of RNA required for recovery, particularly for photosynthesis (a category also upregulated in the LRD). *X. schlechteri* has been shown to continue to select RNA transcription in the LRD, with transcripts associated with photosynthesis being stably stored until translation upon recovery [16,40]. Intriguingly, cellular respiration is upregulated at the LRD, as indicated by an increase in phosphoglycerate kinase (GO: 0006096), a protein involved in glycolysis, and Mundree and Farrant (2000) have shown that measurable respiration in this species continues until the RWC is as low as 10% [14].

As in the MRD, a number of proteins involved in photosynthesis, carbohydrate metabolism, protein homeostasis, redox homeostasis, cell wall organization, coenzyme metabolism and protein biosynthesis were downregulated in the LRD (Figure 5, Supplementary Table S15), again pointing to a slowing of particular metabolisms at low RWCs. The proteins downregulated in photosynthesis—LHCb1/2/3 of the LHC-II complex, PsbO and PsaD—point to continued dismantling of the photosynthetic apparatus and the prevention of ROS generation. The decline in redox homeostasis was due to lower production of redox enzymes (Supplementary Table S15) and did not necessarily mean a decline in redox potential, as it has been shown that such enzymes remain capable of activity at low water contents in *X. schlechteri* [14,15,25].

## 5. Conclusions

*X. schlechteri* undergoes numerous biological, molecular and metabolic changes in association with progressive dehydration and desiccation which, in addition to facilitating desiccation tolerance, also prepares the plant for reconstitution of the biological and metabolic processes upon rehydration. This study investigated the proteomic response of this species to dehydration and desiccation, using a metabolomics study to validate the observed changes and correlate the protein findings with the observable and reported biochemical and physiological responses to a water deficit and recovery from it. This approach proved highly successful in identifying and confirming the key processes or hallmarks of vegetative desiccation tolerance in a poikilocholorophyllous monocot species. Among these are the clear osmotic adjustment, induction of secondary metabolism and antioxidant potential during early dehydration, marked downregulation of photosynthetic metabolism and switches from growth promotion (carbon source) to accumulation of protective processes (sink) during further dehydration to 40% RWC and below this, with notable but limited ongoing metabolism geared toward entry into a biophysically stable quiescent state fully prepared for recovery upon rehydration. Furthermore, our study adds to the existing transcriptomic and genomic information of desiccation tolerance in *X. schlechteri* [16,40] which, together with numerous physiological studies [14,15,18], makes this the most comprehensively documented monocot resurrection plant to date and widens its known potential as a model [13] for the ultimate production of drought-tolerant cereals.

## Data Availability

Data are contained within the article or Supplementary Materials. The unprocessed proteomics data used in this study are available at ProteomeXchange as PXD029411.

## Supplementary Materials

The following are available online at https://www.mdpi.com/article/10.3390/life11111242/s1. Supplementary Tables S1–S15. Proteomics data are available from ProteomeXchange as PXD029411. Table S1: List of differentially expressed proteins involved early response to drying identified in X. schlechteri leaf samples. Table S2: List of differentially expressed proteins involved mid response to drying identified in X. schlechteri leaf samples. Table S3: List of differentially expressed proteins involved Late response to drying identified in X. schlechteri leaf samples. Table S4: Upregulated proteins identified in X. schlechteri leaf tissue in response to dehydration at different dehydration stages. Early Response to Dehydration (ERD), Mid Response to Dehydration (MRD) and Late Response to Dehydration (LRD). Table S5: Down-regulated proteins identified in X. schlechteri leaf tissue in response to dehydration at different dehydration stages. Early Response to Dehydration (ERD), Mid Response to Dehydration (MRD) and Late Response to Dehydration (LRD). Table S6: List of Xerophyta schlechteri unknown proteins identified during label-free proteomics. Table S7: Mapman BINs representing upregulated and downregulated X. schlechteri proteins at early response to drying. Table S8: Mapman BINs representing upregulated and downregulated X. schlechteri proteins at Mid response to drying. Table S9: Mapman BINs representing upregulated and downregulated X. schlechteri proteins at late response to drying. Table S10: List of the Bin names, id and description of the upregulated proteins at ERD. Table S11: List of the Bin names, id and description of the downregulated proteins at ERD. Table S12: List of the Bin names, id and description of the upregulated proteins at MRD. Table S13: List of the Bin names, id and description of the downregulated proteins at MRD. Table S14: List of the Bin names, id and description of the upregulated proteins at LRD. Table S15: List of the Bin names, id and description of the downregulated proteins at LRD.

## Abbreviations

Relative water content (RWC), early response to dehydration (ERD), mid-response to dehydration (MRD), late response to dehydration (LRD), heat shock proteins (HSP), late embryogenesis abundant proteins (LEAs), formic acid (FA), acetonitrile (ACN), liquid chromatography–mass spectrometry (LC-MS), gas chromatography–mass spectrometry (GC-MS), gibberellic acid (GA), jasmonyl-l-94 isoleucine (JA lle), salicylic acid (SA), indole-3-carboxylic acid (ICA), jasmonate (JA), indole-3-acetic acid (IAA-Asp), abscisic acid (ABA), indole-3-butyric acid (IBA).
