# Supplementary material for: A Label-Free Proteomic and Complementary Metabolomic Analysis of Leaves of the Resurrection Plant Xerophyta schlechteri during Dehydration
Source: Life (Basel). 2021 Nov 16;11(11):1242. doi: 10.3390/life11111242 (PMC8624122; doi:10.3390/life11111242)
Supplement: Supplementary file 1 [file life-11-01242-s001.zip › life-1448369-supplementary-done.pdf]

life

MDPI

Supplementary Materials

A Label-Free Proteomic and Complementary Metabolomic Analysis of Leaves of the Resurrection Plant *Xerophyta schlechteri* during Dehydration

Hawwa Gabier [1], David L. Tabb [2], Jill M. Farrant [1] and Mohammed Suhail Rafudeen [1,*]

[1.] Department of Molecular and Cell Biology, University of Cape Town, Private Bag X3, Rondebosch 7701, South Africa; hawwa.gabier@alumni.uct.ac.za-(H.G.); jill.farrant@uct.ac.za (J.M.F.)
[2.] Institute of Infectious Disease and Molecular Medicine, Faculty of Health Sciences, University of Cape Town, Cape Town 7700, South Africa; dtabb@sun.ac.za
[3.] Division of Molecular Biology and Human Genetics, Faculty of Medicine and Health Sciences, Stellenbosch University, Cape Town 7500, South Africa
[4.] Centre for Bioin-formatics and Computational Biology, Stellenbosch University, Stellenbosch 7602, South Africa
* Correspondence: suhail.rafudeen@uct.ac.za

Table S1: List of differentially expressed proteins involved early response to drying identified in *X. schlechteri* leaf samples.

Accession	Description	base M ean	log2FoldC hange	p-value	InterPro	Go-term
**Xvis03_221564-PA**	processed-gene-12.109- mRNA-1protein Name:"Similar to Late embryogenesis abundant protein D-34 (Gossypium hirsutum)" AED:0.11 eAED:0.19 QI:0|0|0|1|1|1|2|0|254	2.2082 56511	2.07869733	0.003377 706	IPR00701 1 (PFAM)	no GO terms
**Xvis03_200886-PA**	augustus-gene-6.42- mRNA-1 protein Name:"Similar to PLP1 Patatin-like protein 1 (Oryza sativa subsp. japonica)" AED:0.26 eAED:0.26 QI:72|0.4|0.16|1|1|1|6|0|3 1 4	2.8898 69909	1.55299452 9	0.004367 183	IPR00264 1 (PFAM)	P:GO:000662 9
**Xvis03_220002-PA**	augustus-gene-50.23-mRNA-1 protein Name:"Similar to Desiccation-related protein PCC3-06 (Craterostigma plantagineum)" AED:0.00 eAED:0.00 QI:100|1|1|1|1|1|2|285|21 8	8.3481 686	1.70902689 7	0.007333 848		no GO terms

**Xvis03_225004-PA**	processed-gene-0.29-mRNA-1 protein Name:"Similar to GLU Ferredoxin-dependent glutamate synthase, chloroplastic (Oryza sativa subsp. japonica)" AED:0.15 eAED:0.20 QI:0|0.82|0.86|0.94|1|1|36|0|1763	4.538397579	1.093872984	0.010961575	IPR017932 (PFAM)	
**Xvis03_211917-PA**	augustus-gene-8.2- mRNA-1 protein Name:"Similar to UGT78D2 UDP-glycosyltransferase 78D2 (Arabidopsis thaliana)" AED:0.00 eAED:0.00 QI:173|1|1|1|1|1|2|210|454	1.696612041	1.920989868	0.011244498	IPR002213 (PFAM)	F:GO:0016758
**Xvis03_202087-PA**	augustus-gene-8.14-mRNA-1 protein Name:"Similar to AAE3 Oxalate--CoA ligase (Arabidopsis thaliana)" AED:0.02 eAED:0.02 QI:521|1|1|1|1|1|4|258|517	2.212368933	1.706599422	0.01303	IPR000873 (PFAM)	F:GO:0003824
**Xvis03_203578-PA**	processed-gene-12.33- mRNA-1 protein Name:"Similar to Polyphenol oxidase, chloroplastic (Vitis vinifera)" AED:0.36 eAED:0.40 QI:797|0.5|0.33|1|1|1|3|0|273	4.551593209	0.950857782	0.016000633	IPR002227	F:GO:0004097;F:GO:0016491;P:GO:0055114
**Xvis03_211325-PA**	augustus-gene-1.70- mRNA-1 protein Name:"Similar to GDCSPA Glycine Dehydrogenase (decarboxylating) A, mitochondrial (Flaveria pringlei)" AED:0.11 eAED:0.11 QI:248|0.76|0.85|1|1|1|14|321|903	10.76576001	0.729163862	0.01618098	IPR002591 (PFAM);	F:GO:0003824;F:GO:0004375;P:GO:0006546; P:GO:005514
**Xvis03_221126-PA**	processed-gene-11.3-mRNA-1 protein Name:"Similar to Mavicyanin (Cucurbita pepo)" AED:0.03 eAED:0.03 QI:68|1|1|1|1|1|2|217|190	0.735849705	1.86719922	0.01686627	IPR008972	F:GO:000905

**Xvis03_225456-PA**	processed-gene-0.12- mRNA-1 protein Name:"Similar to Polyphenol oxidase, chloroplastic (Vitis vinifera)" AED:0.01 eAED:0.01 QI:289|1|1|1|1|2|284|597	3.953237343	0.91393486	0.026841309	IPR002227 (PFAM)	F:GO:0004097;F:GO:0016491;P:GO:0055114
**Xvis03_222086-PA**	processed-gene-4.0- mRNA-1 protein Name:"Similar to SBT3.3 Subtilisin-like protease SBT3.3 (Arabidopsis thaliana)" AED:0.15 eAED:0.15 QI:86|0.88|0.8|1|1|0.9|10|0|661	7.081485008	0.906750846	0.028952361	IPR037045	no GO terms
**Xvis03_215744-PA**	processed-gene-5.46- mRNA-1 protein Name:"Similar to CAL1 Calmodulin (Medicago sativa)" AED:0.17 eAED:0.17 QI:127|1|1|1|1|2|496|149	0.884696437	1.635472647	0.030612647	IPR002048 (PFAM)	F:GO:0005509;P:GO:0019722
**Xvis03_210936-PA**	augustus-gene-1.31- mRNA-1 protein Name:"Similar to At5g61250 Heparanase-like protein 2 (Arabidopsis thaliana)" AED:0.14 eAED:0.14 QI:264|0.22|0.2|1|0.88|0.9|10|0|512	4.241227829	0.954132089	0.034425962	IPR005199 (PFAM)	C:GO:0016020;F:GO:0016798
**Xvis03_225004-PA**	augustus-gene-8.38- mRNA-1 protein Name:"Similar to GLU Ferredoxin-dependent glutamate synthase, chloroplastic (Oryza sativa subsp. japonica)" AED:0.12 eAED:0.12 QI:225|0.96|1|1|0.96|1|32|887|1535	4.627647757	0.95110059	0.036775888	IPR036485	no GO terms
**Xvis03_207220-PA**	augustus-gene-29.99- mRNA-1 protein Name:"Similar to Bowman-Birk type trypsin	1.467485389	1.490311914	0.03752355	IPR000877 (PFAM)	F:GO:0004867;C:GO:0005576

	inhibitor (Triticum aestivum)" AED:0.14 eAED:0.14 QI:36|1|1|1|1|1|2|205|120					
**Xvis03_225299-PA**	processed-gene-0.7- mRNA-1 protein Name:"Similar to AGT1 Serine--glyoxylate aminotransferase (Arabidopsis thaliana)" AED:0.11 eAED:0.11 QI:101|1|1|1|0.8|0.66|6|208|401	5.45824752	0.795651548	0.042740279	IPR015421	F:GO:0003824
**Xvis03_206589-PA**	processed-gene-9.106-mRNA-1 protein Name:"Similar to CYP74A2 Allene oxide synthase 2 (Oryza sativa subsp. japonica)" AED:0.00 eAED:0.00 QI:0|-1|0|1|-1|1|1|0|486	4.189543295	0.859536898	0.042986185	IPR001128 (PFAM)	F:GO:000556;F:GO:00165 F:GO:0020037;P:GO:0055114
**Xvis03_221325-PA**	processed-gene-6.29- mRNA-1 protein Name:"Similar to B19.4 Late embryogenesis abundant protein B19.4 (Hordeum vulgare)" AED:0.16 eAED:0.16 QI:0|0.66|0.5|0.75|1|1|4|11 7|181	3.843070937	1.159483161	0.044432356	IPR038956 (PFAM)	no GO terms
**Xvis03_223476-PA**	augustus-gene-2.101-mRNA-1 protein Name:"Similar to Pyruvate kinase, cytosolic isozyme (Glycine max)" AED:0.04 eAED:0.04 QI:138|1|1|1|1|1|3|406|533	3.260231664	0.98750497	0.047705112	IPR001697	F:GO:0000287;F:GO:0003824;F:GO:0004743;P:GO:0006096;F:GO:0030955
**Xvis03_223774-PA**	processed-gene-15.13- mRNA-1 protein Name:"Similar to Late embryogenesis abundant protein 76 (Brassica napus)" AED:0.02 eAED:0.02 QI:314|1|1|1|1|1|3|870|266	3.830648677	1.533858234	0.047990256	IPR004864 (PFAM	no GO terms
**Xvis03_221252-PA**	processed-gene-1.52-mRNA-1 protein Name:"Similar to SPG20 Spartin (Homo sapiens)" AED:0.03 eAED:0.03	2.614950623	1.383758686	0.049376875	IPR009686	no GO terms

	QI:113|1|1|1|1|1|4|834|401					

Table S2: List of differentially expressed proteins involved mid response to drying identified in *X. schlechteri* leaf samples.

Accession	Description	base Mean	log2FoldChange	pvalue	InterPro	Go-Terms
**Xvis03_220002-PA**	processed-gene-8.116-mRNA-1 protein Name:"Similar to Desiccation-related protein PCC3-06 (Craterostigma plantagineum)" AED:0.00 eAED:0.00 QI:334|1|1|1|1|1|2|386|231	8.34817	2.74168	1.13E-05		no GO terms
**Xvis03_202087-PA**	augustus-gene-8.14-mRNA-1 protein Name:"Similar to AAE3 Oxalate--CoA ligase (Arabidopsis thaliana)" AED:0.02 eAED:0.02 QI:521|1|1|1|1|1|4|258|517	2.21237	2.3688	0.00046	IPR000873 (PFAM)	F:GO:0003824
**Xvis03_204003-PA**	augustus-gene-27.86-mRNA-1 protein Name:"Similar to Aldose reductase (Hordeum vulgare)" AED:0.09 eAED:0.09 QI:204|1|1|1|1|1|9|270|319	3.17441	1.94368	0.0005	IPR020471	F:GO:0016491; P:GO:0055114
**Xvis03_211917-PA**	augustus-gene-8.2-mRNA-1 protein Name:"Similar to UGT78D2 UDP-glycosyltransferase 78D2 (Arabidopsis thaliana)" AED:0.00 eAED:0.00 QI:173|1|1|1|1|1|2|210|454	1.69661	2.56191	0.00066	IPR002213 (PFAM)	F:GO:0016758
**Xvis03_215617-PA**	late embryogenesis abundant protein D-29	3.29873	2.03005	0.0009		no GO terms

**Xvis03_221325-PA**	processed-gene-6.29-mRNA-1 protein Name:"Similar to B19.4 Late embryogenesis abundant protein B19.4 (Hordeum vulgare)" AED:0.16 eAED:0.16 QI:0|0.66|0.5|0.75|1|1|4|117|181	3.84307	1.81639	0.00126	IPR038956 (PFAM)	no GO terms
**Xvis03_210802-PA**	processed-gene-9.8-mRNA-1 protein Name:"Similar to LTI65 Low-temperature-induced 65 kDa protein (Arabidopsis thaliana)" AED:0.00 eAED:0.00 QI:247|1|1|1|1|1|5|289|574	3.50713	2.00575	0.00161		P:GO:0009737
**Xvis03_216950-PA**	augustus-gene-2.19-mRNA-1 protein Name:"Similar to 11 kDa late embryogenesis abundant protein (Helianthus annuus)" AED:0.13 eAED:0.13 QI:0|0.5|0.33|0.66|1|1|3|337|255	5.28524	1.65506	0.00189	IPR005513 (PFAM	P:GO:0009793
**Xvis03_221252-PA**	augustus-gene-6.27-mRNA-1 protein Name:"Similar to SPG20 Spartin (Homo sapiens)" AED:0.04 eAED:0.04 QI:93|1|1|1|0.75|0.6|5|21|403	2.61495	2.13635	0.00196	IPR009686 (PFAM);	no GO terms
**Xvis03_219751-PA**	augustus-gene-2.77-mRNA-1 protein Name:"Similar to GAPC3 Glyceraldehyde-3-phosphate dehydrogenase 3, cytosolic (Oryza sativa subsp. japonica)" AED:0.27 eAED:0.27 QI:255|0.9|1|1|1|1|11|366|288	12.4061	0.86947	0.00568	IPR020831	F:GO:0016620; P:GO:0055114
**Xvis03_211956-PA**	embryonic protein DC-8 isoform X2	10.5462	1.45642	0.00591		no GO terms
**Xvis03_221252-PA**	augustus-gene-6.27-mRNA-1 protein Name:"Similar to SPG20 Spartin (Homo sapiens)" AED:0.04 eAED:0.04	1.18863	2.13471	0.00628	IPR009686 (PFAM);	no GO terms

	QI:93|1|1|1|0.75|0.6|5|21|403					
**Xvis03_205230-PA**	processed-gene-22.97-mRNA-1 protein Name:"Similar to RAB18 Dehydrin Rab18 (Arabidopsis thaliana)" AED:0.00 eAED:0.00 QI:93|1|1|1|1|1|2|247|147	5.05544	1.4358	0.00704	IPR000167 (PFAM)	P:GO:0009415
**Xvis03_220790-PA**	augustus-gene-7.89-mRNA- 1 protein Name:"Similar to SYNPCC7002_A1590 Uncharacterized protein SYNPCC7002_A1590 (Synechococcus sp. (strain ATCC 27264 / PCC 7002 / PR-6))" AED:0.04 eAED:0.04 QI:148|1|1|1|1|1|6|744|229	1.30524	2.0387	0.00743	IPR018971 (PFAM)	no GO terms
**Xvis03_221564-PA**	augustus-gene-1.8-mRNA-1 protein Name:"Similar to Late embryogenesis abundant protein D-34 (Gossypium hirsutum)" AED:0.08 eAED:0.23 QI:0|0|0.5|1|1|1|2|238|274	2.20826	1.93331	0.0076	IPR007011 (PFAM)	no GO terms
**Xvis03_223774-PA**	augustus-gene-3.64-mRNA-1 protein Name:"Similar to LEA14-A Late embryogenesis abundant protein Lea14-A (Gossypium hirsutum)" AED:0.40 eAED:0.40 QI:0|0|0|1|1|1|2|0|155	3.55628	1.5758	0.00796	IPR004864 (PFAM	no GO terms
**Xvis03_205230-PA**	processed-gene-22.97-mRNA-1 protein Name:"Similar to RAB18 Dehydrin Rab18 (Arabidopsis thaliana)" AED:0.00 eAED:0.00 QI:93|1|1|1|1|1|2|247|147	7.33068	1.1034	0.01074	IPR000167 (PFAM)	P:GO:0009415

**Xvis03_208058-PA**	Processed-gene-23.43-mRNA-1 protein Name:"Similar to CML18 Probable calcium-binding protein CML18 (Oryza sativa subsp. japonica)" AED: 0.02 eAED: 0.02 QI:0|-1|0|1|-1|1|1|0|161	0.9707	1.97236	0.01121		F:GO:0005509
**Xvis03_218200-PA**	augustus-gene-6.42-mRNA-1 protein Name:"Similar to PLP1 Patatin-like protein 1 (Oryza sativa subsp. japonica)" AED:0.26 eAED:0.26 QI:72|0.4|0.16|1|1|1|6|0|314	2.88987	1.4167	0.01223	IPR002641 (PFAM)	P:GO:0006629
**Xvis03_220002-PA**	processed-gene-8.116-mRNA-1 protein Name:"Similar to Desiccation-related protein PCC3-06 (Craterostigma plantagineum)" AED:0.00 eAED:0.00 QI:334|1|1|1|1|1|2|386|231	8.7458	1.4657	0.01353		no GO terms
**Xvis03_221512-PA**	processed-gene-2.27-mRNA-1 protein Name:"Similar to Late embryogenesis abundant protein 76 (Brassica napus)" AED:0.12 eAED:0.12 QI:0|0.33|0.5|0.75|1|1|4|219|192	1.44462	1.87719	0.01396		no GO terms
**Xvis03_202655-PA**	processed-gene-53.13-mRNA-1 protein Name:"Similar to CCT2 Choline-phosphate cytidylyltransferase 2 (Arabidopsis thaliana)" AED:0.15 eAED:0.15 QI:50|1|1|1|1|1|8|392|291	0.98801	1.89108	0.01521	IPR004821 (PFAM	F:GO:0003824; P:GO:0009058
**Xvis03_221512-PA**	processed-gene-2.27-mRNA-1 protein Name:"Similar to Late embryogenesis abundant	3.83065	1.86211	0.01641		no GO terms

	protein 76 (Brassica napus)" AED:0.12 eAED:0.12 QI:0|0.33|0.5|0.75|1|1|4|219| 192					
**Xvis03_225532-PA**	processed-gene-0.13-mRNA-1 protein Name:"Similar to DHN3 Dehydrin DHN3 (Hordeum vulgare)" AED:0.00 eAED:0.00 QI:270|1|1|1|1|1|2|257|183	6.48103	1.13443	0.01683	IPR000167 (PFAM);	P:GO:0009415
**Xvis03_221564-PA**	augustus-gene-1.8-mRNA-1 protein Name:"Similar to Late embryogenesis abundant protein D-34 (Gossypium hirsutum)" AED:0.08 eAED:0.23 QI:0|0|0.5|1|1|1|2|238|274	1.7858	1.70738	0.01734	IPR007011 (PFAM)	no GO terms
**Xvis03_220240-PA**	augustus-gene-8.38-mRNA- 1 protein Name:"Similar to LEA3 Late embryogenesis abundant protein, group 3 (Oryza sativa subsp. indica)" AED:0.00 eAED:0.00 QI:193|1|1|1|1|1|2|266|174	13.1791	1.18885	0.01741		no GO terms
**Xvis03_220597-PA**	processed-gene-1.22-mRNA-1 protein Name:"Similar to RAB18 Dehydrin Rab18 (Arabidopsis thaliana)" AED:0.00 eAED:0.00 QI:228|1|1|1|1|1|2|308|136	4.64528	1.30412	0.01772	IPR000167 (PFAM	P:GO:0009415
**Xvis03_223451-PA**	Processed-gene-3.71-mRNA-1 protein Name:"Similar to CSP1 Cold shock protein 1 (Arabidopsis thaliana)" AED: 0.30 eAED: 0.30 QI:0|-1|0|1|-1|1|1|0|215	1.65454	1.62548	0.01843	IPR002059	F:GO:0003676; F:GO:0008270
**Xvis03_223476-PA**	augustus-gene-2.101-mRNA-1 protein Name:"Similar to Pyruvate kinase, cytosolic isozyme (Glycine max)" AED:0.04 eAED:0.04 QI:138|1|1|1|1|1|3|406|533	3.02161	1.18192	0.02167	IPR001697	F:GO:0000287; F:GO:0003824; F:GO:0004743; P:GO:0006096; F:GO:0030955

**Xvis03_222223-PA**	processed-gene-2.73-mRNA-1 protein Name:"Similar to HSP70 Heat shock cognate 70 kDa protein (Petunia hybrida)" AED:0.11 eAED:0.11 QI:205|0.5|0.33|0.66|1|1|3|0|710	18.4419	0.60753	0.02505	IPR013126	no GO terms
**Xvis03_210802-PA**	processed-gene-9.8-mRNA-1 protein Name:"Similar to LTI65 Low-temperature-induced 65 kDa protein (Arabidopsis thaliana)" AED:0.00 eAED:0.00 QI:247|1|1|1|1|1|5|289|574	18.1056	1.16935	0.02542		P:GO:0009737
**Xvis03_213342-PA**	processed-gene-10.36-mRNA-1 protein Name:"Similar to Histone H4 (Glycine max)" AED: 0.00 eAED: 0.01 QI: 0|-1|0|1|-1|1|1|0|103	8.21874	0.81696	0.0291	IPR001951	F:GO:0003677; F:GO:0046982
**Xvis03_223476-PA**	augustus-gene-2.101-mRNA-1 protein Name:"Similar to Pyruvate kinase, cytosolic isozyme (Glycine max)" AED:0.04 eAED:0.04 QI:138|1|1|1|1|1|3|406|533	3.53294	1.06002	0.03324	IPR001697	F:GO:0000287;F:GO:0003824; F:GO:0004743; P:GO:0006096; F:GO:0030955
**Xvis03_202976-PA**	augustus-gene-30.2-mRNA-1 protein Name:"Similar to Blue copper protein (Pisum sativum)" AED:0.02 eAED:0.02 QI:94|1|1|1|1|1|2|158|189	0.91783	1.60444	0.03689	IPR008972	F:GO:0009055
**Xvis03_221126-PA**	augustus-gene-1.32-mRNA-1 protein Name:"Similar to Mavicyanin (Cucurbita pepo)" AED:0.01 eAED:0.01 QI:65|1|1|1|1|1|2|153|186	0.73585	1.63058	0.03825	IPR008972)	F:GO:0009055
**Xvis03_220240-PA**	augustus-gene-8.38-mRNA-1 protein Name:"Similar to LEA3 Late embryogenesis abundant protein, group 3 (Oryza sativa subsp. indica)" AED:0.00 eAED:0.00	7.28924	1.13228	0.03889		no GO terms

QI:193|1|1|1|1|1|2|266|174

**Xvis03_221252-PA**	augustus-gene-6.27-mRNA-1 protein Name:"Similar to SPG20 Spartin (Homo sapiens)" AED:0.04 eAED:0.04 QI:93|1|1|1|0.75|0.6|5|21|403	2.68011	1.34372	0.03974	IPR009686 (PFAM)	no GO terms
**Xvis03_205813-PA**	processed-gene-6.86-mRNA-1 protein Name:"Similar to RUB2 Ubiquitin-NEDD8-like protein RUB2 (Oryza sativa subsp. japonica)" AED:0.04 eAED:0.04 QI:73|1|1|1|1|1|3|301|153	7.62962	0.8021	0.04235	IPR019956 (PRINTS)	F:GO:0005515
**Xvis03_209096-PA**	processed-gene-16.57-mRNA-1 protein Name:"Similar to ABP20 Auxin-binding protein ABP20 (Prunus persica)" AED:0.02 eAED:0.02 QI:173|1|1|1|1|1|2|671|206	9.19049	0.7264	0.04322	IPR001929 (PRINTS)	F:GO:0030145; F:GO:0045735
**Xvis03_202124-PA**	processed-gene-11.0-mRNA-1 protein Name:"Similar to UGPA UTP--glucose-1-phosphate uridylyltransferase (Musa acuminata)" AED:0.18 eAED:0.18 QI:271|0.88|0.89|1|1|1|19|372|458	8.90801	0.65467	0.0444		F:GO:0003983; P:GO:0006011; F:GO:0070569
**Xvis03_204659-PA**	augustus-gene-39.13-mRNA-1 protein Name:"Similar to PHS2 Alpha-glucan phosphorylase 2, cytosolic (Arabidopsis thaliana)" AED:0.23 eAED:0.23 QI:181|0.92|0.93|1|0.92|0.86|15|169|732	2.41403	1.37564	0.04789	IPR000811	F:GO:0004645; P:GO:0005975; F:GO:0008184; F:GO:0030170

**Xvis03_221564-PA**	augustus-gene-1.8-mRNA-1 protein Name:"Similar to Late embryogenesis abundant protein D-34 (Gossypium hirsutum)" AED:0.08 eAED:0.23 QI:0|0|0.5|1|1|1|2|238|274	0.97016	1.55308	0.04808	IPR007011 (PFAM);	no GO terms
**Xvis03_209643-PA**	Processed-gene-16.29-mRNA-1 protein Name:"Similar to ERF1-3 Eukaryotic peptide chain release factor subunit 1-3 (Arabidopsis thaliana)" AED: 0.00 eAED: 0.00 QI:0|-1|0|1|-1|1|1|0|43	0.91371	1.54312	0.04923		F:GO:0003747; C:GO:0005737; P:GO:0006415

Table S3: List of differentially expressed proteins involved late response to drying identified in *X. schlechteri* leaf samples.

Accession	Description	base Mean	log2FoldChange	p-value	InterPro	Go-Terms
**Xvis03_220002-PA**	processed-gene-8.116-mRNA-1 protein Name:"Similar to Desiccation-related protein PCC3-06 (Craterostigma plantagineum)" AED:0.00 eAED:0.00 QI:334|1|1|1|1|1|2|386|231	8.7458046 6	3.3101873 2	3.45E-09		no GO terms
**Xvis03_220597-PA**	processed-gene-1.22-mRNA-1 protein Name:"Similar to RAB18 Dehydrin Rab18 (Arabidopsis thaliana)" AED:0.00 eAED:0.00 QI:228|1|1|1|1|1|2|308|136	5.0554445 4	2.6869117	3.86E-08	IPR000167 (PFAM	P:GO:0009415
**Xvis03_210802-PA**	processed-gene-9.8-mRNA-1 protein Name:"Similar to LTI65 Low-temperature-induced 65 kDa protein (Arabidopsis thaliana)" AED:0.00 eAED:0.00 QI:247|1|1|1|1|1|5|289|574	3.5071254 8	3.1803771	9.73E-08	IPR037491 (PANTHER)	P:GO:0009737
**Xvis03_216950-PA**	augustus-gene-2.19-mRNA-1 protein Name:"Similar to 11 kDa late embryogenesis abundant protein (Helianthus annuus)" AED:0.13 eAED:0.13 QI:0|0.5|0.33|0.66|1|1|3|337|255	5.2852439 6	2.5576639 8	3.13E-07	IPR005513 (PFAM)	P:GO:0009793

**Xvis03_222911-PA**	processed-gene-1.35-mRNA-1 protein Name:"Similar to CHLI Magnesium-chelatase subunit ChlI, chloroplastic (Oryza sativa subsp. japonica)" AED:0.00 eAED:0.00 QI:218|1|1|1|1|1|3|269|418	4.08563214	2.65244542	3.22E-07	IPR000523 (PFAM)	F:GO:0005524; P:GO:0015995; F:GO:0016851
**Xvis03_221512-PA**	processed-gene-2.27-mRNA-1 protein Name:"Similar to Late embryogenesis abundant protein 76 (Brassica napus)" AED:0.12 eAED:0.12 QI:0|0.33|0.5|0.75|1|1|4|219|192	3.83064868	3.86180989	3.60E-07		no GO terms
**Xvis03_220363-PA**	augustus-gene-1.54-mRNA-1 protein Name:"Similar to ECU03_1610 Uncharacterized protein ECU03_1610 (Encephalitozoon cuniculi (strain GB-M1))" AED:0.03 eAED:0.03 QI:89|1|1|1|1|1|2|303|232	3.9401913	3.03913605	4.19E-07		no GO terms
**Xvis03_200762-PA**	processed-gene-88.24-mRNA-1 protein Name:"Similar to At4g13010 Putative quinone-oxidoreductase homolog, chloroplastic (Arabidopsis thaliana)" AED:0.26 eAED:0.26 QI:0|1|0.75|1|1|1|4|216|327	4.97092425	2.45863907	4.22E-07	IPR013154 (PFAM	P:GO:0055114
**Xvis03_218117-PA**	augustus-gene-11.3-mRNA-1 protein Name:"Similar to Embryonic protein DC-8 (Daucus carota)" AED:0.04 eAED:0.04 QI:0|1|0.66|1|1|1|3|517|510	10.5461814	2.54581223	5.48E-07	PTHR33836:SF1	P:GO:0009737
**Xvis03_221252-PA**	augustus-gene-6.27-mRNA-1 protein Name:"Similar to SPG20 Spartin (Homo sapiens)" AED:0.04 eAED:0.04 QI:93|1|1|1|0.75|0.6|5|21|403	2.61495062	3.21616006	8.75E-07	IPR009686 (PFAM)	no GO terms
**Xvis03_221564-PA**	augustus-gene-1.8-mRNA-1 protein Name:"Similar to Late embryogenesis abundant protein D-34 (Gossypium	3.29872737	2.82360633	1.02E-06	IPR007011 (PFAM	no GO terms

	hirsutum)" AED:0.08 eAED:0.23 QI:0|0|0.5|1|1|1|2|238|274					
**Xvis03_222911-PA**	processed-gene-1.35-mRNA-1 protein Name:"Similar to CHLI Magnesium-chelatase subunit ChlI, chloroplastic (Oryza sativa subsp. japonica)" AED:0.00 eAED:0.00 QI:218|1|1|1|1|1|3|269|418	2.71147713	2.66300275	1.80E-06	IPR000523 (PFAM);	F:GO:0005524; P:GO:0015995; F:GO:0016851
**Xvis03_221325-PA**	processed-gene-6.29-mRNA-1 protein Name:"Similar to B19.4 Late embryogenesis abundant protein B19.4 (Hordeum vulgare)" AED:0.16 eAED:0.16 QI:0|0.66|0.5|0.75|1|1|4|117|181	3.84307094	2.5169091	1.85E-06	IPR038956 (PFAM)	no GO terms
**Xvis03_212894-PA**	augustus-gene-2.1-mRNA-1 protein Name:"Similar to PORB Protochlorophyllide reductase B, chloroplastic (Hordeum vulgare)" AED:0.09 eAED:0.09 QI:162|1|1|1|1|1|5|457|393	3.05148999	2.11243109	2.74E-06	IPR002347 (PRINTS)	F:GO:0016630; P:GO:0055114
**Xvis03_220239-PA**	augustus-gene-8.37-mRNA-1 protein Name:"Similar to Late embryogenesis abundant protein Dc3 (Daucus carota)" AED:0.00 eAED:0.00 QI:288|1|1|1|1|1|2|197|146	3.70555605	2.62485384	3.81E-06		no GO terms
**Xvis03_220597-PA**	processed-gene-1.22-mRNA-1 protein Name:"Similar to RAB18 Dehydrin Rab18 (Arabidopsis thaliana)" AED:0.00 eAED:0.00 QI:228|1|1|1|1|1|2|308|136	7.33068104	1.81808993	5.89E-06	IPR000167 (PFAM)	P:GO:0009415
**Xvis03_221564-PA**	augustus-gene-1.8-mRNA-1 protein Name:"Similar to Late embryogenesis abundant protein D-34 (Gossypium hirsutum)" AED:0.08 eAED:0.23 QI:0|0|0.5|1|1|1|2|238|274	2.20825651	3.08392405	6.38E-06	IPR007011 (PFAM)	no GO terms

**Xvis03_220240-PA**	augustus-gene-8.38-mRNA-1 protein Name:"Similar to LEA3 Late embryogenesis abundant protein, group 3 (Oryza sativa subsp. indica)" AED:0.00 eAED:0.00 QI:193|1|1|1|1|1|2|266|174	7.28923941	2.29795564	8.88E-06		no GO terms
**Xvis03_220790-PA**	augustus-gene-7.89-mRNA-1 protein Name:"Similar to SYNPCC7002_A1590 Uncharacterized protein SYNPCC7002_A1590 (Synechococcus sp. (strain ATCC 27264 / PCC 7002 / PR-6))" AED:0.04 eAED:0.04 QI:148|1|1|1|1|1|6|744|229	1.30524151	3.2089502	9.54E-06	IPR018971 (PFAM)	no GO terms
**Xvis03_221252-PA**	augustus-gene-6.27-mRNA-1 protein Name:"Similar to SPG20 Spartin (Homo sapiens)" AED:0.04 eAED:0.04 QI:93|1|1|1|0.75|0.6|5|21|403	2.68010885	2.63188195	1.13E-05	IPR009686 (PFAM)	no GO terms
**Xvis03_221564-PA**	augustus-gene-1.8-mRNA-1 protein Name:"Similar to Late embryogenesis abundant protein D-34 (Gossypium hirsutum)" AED:0.08 eAED:0.23 QI:0|0|0.5|1|1|1|2|238|274	1.78580156	2.88970967	1.70E-05	IPR007011 (PFAM)	no GO terms
**Xvis03_218117-PA**	augustus-gene-11.3-mRNA-1 protein Name:"Similar to Embryonic protein DC-8 (Daucus carota)" AED:0.04 eAED:0.04 QI:0|1|0.66|1|1|1|3|517|510	4.66984757	2.51898187	1.74E-05		P:GO:0009737
**Xvis03_225532-PA**	processed-gene-0.13-mRNA-1 protein Name:"Similar to DHN3 Dehydrin DHN3 (Hordeum vulgare)" AED:0.00 eAED:0.00 QI:270|1|1|1|1|1|2|257|183	6.48103019	1.88977715	2.04E-05	IPR000167 (PFAM)	P:GO:0009415
**Xvis03_202087-PA**	augustus-gene-8.14-mRNA-1 protein Name:"Similar to AAE3 Oxalate--CoA ligase	2.21236893	2.65731454	5.13E-05	IPR000873 (PFAM)	F:GO:0003824

	(Arabidopsis thaliana)" AED:0.02 eAED:0.02 QI:521|1|1|1|1|1|4|258|517					
**Xvis03_204003-PA**	augustus-gene-27.86-mRNA-1 protein Name:"Similar to Aldose reductase (Hordeum vulgare)" AED:0.09 eAED:0.09 QI:204|1|1|1|1|1|9|270|319	3.1744073	2.13724642	6.79E-05	IPR020471	F:GO:0016491; P:GO:0055114
**Xvis03_203592-PA**	augustus-gene-31.41-mRNA-1 protein Name:"Similar to 1-Cys peroxiredoxin (Medicago truncatula)" AED:0.00 eAED:0.00 QI:253|1|1|1|1|1|2|509|219	9.70387224	1.3351265	7.04E-05	IPR000866	F:GO:0016209; F:GO:0016491; P:GO:0045454; F:GO:0051920; P:GO:0055114
**Xvis03_205034-PA**	late embryogenesis abundant protein D-34-like	1.19585914	3.09035173	7.65E-05	IPR007011 (PFAM)	no GO terms
**Xvis03_220240-PA**	augustus-gene-8.38-mRNA-1 protein Name:"Similar to LEA3 Late embryogenesis abundant protein, group 3 (Oryza sativa subsp. indica)" AED:0.00 eAED:0.00 QI:193|1|1|1|1|1|2|266|174	13.1791276	1.91790689	7.91E-05		no GO terms
**Xvis03_220597-PA**	processed-gene-1.22-mRNA-1 protein Name:"Similar to RAB18 Dehydrin Rab18 (Arabidopsis thaliana)" AED:0.00 eAED:0.00 QI:228|1|1|1|1|1|2|308|136	4.64527916	2.04137833	8.67E-05	IPR000167 (PFAM)	P:GO:0009415
**Xvis03_220002-PA**	processed-gene-8.116-mRNA-1 protein Name:"Similar to Desiccation-related protein PCC3-06 (Craterostigma plantagineum)" AED:0.00 eAED:0.00 QI:334|1|1|1|1|1|2|386|231	25.9049083	1.66968991	0.000135873		no GO terms
**Xvis03_221720-PA**	augustus-gene-5.43-mRNA-1 protein Name:"Similar to EIF-5A2 Eukaryotic translation initiation factor 5A-2 (Nicotiana plumbaginifolia)" AED:0.05 eAED:0.05 QI:177|1|1|1|0.8|0.66|6|330|160	2.1835228	2.09572901	0.000160339	IPR020189 (PFAM)	F:GO:0003723; F:GO:0003746; P:GO:0006452; F:GO:0043022; P:GO:0045901; P:GO:0045905

**Xvis03_223454-PA**	processed-gene-3.78-mRNA-1 protein Name:"Similar to PCKR1 Peptidyl-prolyl cis-trans isomerase (Catharanthus roseus)" AED:0.02 eAED:0.02 QI:0|-1|0|1|-1|1|1|0|173	7.4809848 9	1.26642199	0.000163465	IPR002130	P:GO:0000413; F:GO:0003755
**Xvis03_211917-PA**	augustus-gene-8.2-mRNA-1 protein Name:"Similar to UGT78D2 UDP-glycosyltransferase 78D2 (Arabidopsis thaliana)" AED:0.00 eAED:0.00 QI:173|1|1|1|1|1|2|210|454	1.6966120 4	2.78513244	0.00016923	IPR002213 (PFAM);	F:GO:0016758
**Xvis03_221252-PA**	augustus-gene-6.27-mRNA-1 protein Name:"Similar to SPG20 Spartin (Homo sapiens)" AED:0.04 eAED:0.04 QI:93|1|1|1|0.75|0.6|5|21|403	1.1886259	2.87087914	0.000184897	IPR009686 (PFAM)	no GO terms
**Xvis03_210802-PA**	processed-gene-9.8-mRNA-1 protein Name:"Similar to LTI65 Low-temperature-induced 65 kDa protein (Arabidopsis thaliana)" AED:0.00 eAED:0.00 QI:247|1|1|1|1|1|5|289|574	18.105578 8	1.90906986	0.000202692	IPR037491 (PANTHER)	P:GO:0009737
**Xvis03_221564-PA**	augustus-gene-1.8-mRNA-1 protein Name:"Similar to Late embryogenesis abundant protein D-34 (Gossypium hirsutum)" AED:0.08 eAED:0.23 QI:0|0|0.5|1|1|1|2|238|274	0.9701643 4	2.81667177	0.000227483	IPR007011 (PFAM)	no GO terms
**Xvis03_225199-PA**	processed-gene-0.101-mRNA-1 protein Name:"Similar to Aldose reductase (Hordeum vulgare)" AED:0.14 eAED:0.14 QI:154|0.87|0.88|1|0.62|0.55|9|25|337	6.8555564 5	1.61453776	0.000230193	IPR020471	F:GO:0016491; P:GO:0055114
**Xvis03_220240-PA**	augustus-gene-8.38-mRNA-1 protein Name:"Similar to LEA3 Late embryogenesis abundant protein, group 3 (Oryza sativa subsp. indica)" AED:0.00 eAED:0.00 QI:193|1|1|1|1|1|2|266|174	10.717152 8	2.54688193	0.000231897		no GO terms
**Xvis03_209653-PA**	processed-gene-17.23-mRNA-1 protein Name:"Similar to Os01g0270100 Cysteine proteinase inhibitor 12 (Oryza sativa subsp. japonica)" AED:0.07 eAED:0.07 QI:0|0|0|1|1|1|4|0|225	6.0337838 4	1.29142461	0.000294989		F:GO:0004869

**Xvis03_214372-PA**	processed-gene-6.62-mRNA-1 protein Name:"Similar to Eukaryotic translation initiation factor 5A (Manihot esculenta)" AED:0.20 eAED:0.20 QI:188|1|1|1|0.8|0.66|6|353|159	2.36761697	1.93495142	0.000335124	IPR020189 (PFAM	F:GO:0003723; F:GO:0003746; P:GO:0006452; F:GO:0043022; P:GO:0045901; P:GO:0045905
**Xvis03_217638-PA**	augustus-gene-2.63-mRNA-1 protein Name:"Similar to CCR1 Cinnamoyl-CoA reductase 1 (Arabidopsis thaliana)" AED:0.07 eAED:0.07 QI:0|0|0|1|1|1|6|0|324	1.42922142	2.60042155	0.000362868	IPR036291 (SUPERFAMILY)	F:GO:0003824; F:GO:0050662
**Xvis03_201015-PA**	augustus-gene-109.105-mRNA-1 protein Name:"Similar to MSRB5 Peptide methionine sulfoxide reductase B5 (Oryza sativa subsp. japonica)" AED:0.11 eAED:0.11 QI:31|0.33|0.5|1|1|1|4|790|250	1.02003153	2.59500971	0.000419649	IPR002579 (PFAM	P:GO:0006979; F:GO:0016671; P:GO:0030091; F:GO:0033743; P:GO:0055114
**Xvis03_223774-PA**	augustus-gene-3.64-mRNA-1 protein Name:"Similar to LEA14-A Late embryogenesis abundant protein Lea14-A (Gossypium hirsutum)" AED:0.40 eAED:0.40 QI:0|0|0|1|1|1|2|0|155	2.60953673	2.08462994	0.000431468	IPR004864 (PFAM);	no GO terms
**Xvis03_209643-PA**	processed-gene-16.29-mRNA-1 protein Name:"Similar to ERF1-3 Eukaryotic peptide chain release factor subunit 1-3 (Arabidopsis thaliana)" AED:0.00 eAED:0.00 QI:0|-1|0|1|-1|1|1|0|437	0.99538385	2.67709713	0.000456625	IPR005142 (PFAM);	F:GO:0003747; C:GO:0005737; P:GO:0006415
**Xvis03_222292-PA**	processed-gene-3.63-mRNA-1 protein Name:"Similar to CML18 Probable calcium-binding protein CML18 (Oryza sativa subsp. japonica)" AED:0.02 eAED:0.02 QI:0|-1|0|1|-1|1|1|0|161	0.97070487	2.64320394	0.000502823	IPR002048 (PFAM);	F:GO:0005509
**Xvis03_206202-PA**	processed-gene-9.17-mRNA-1 protein Name:"Similar to GMPM1 18 kDa seed maturation protein (Glycine max)" AED:0.23 eAED:0.23 QI:0|-1|0|1|-1|1|1|0|132	0.77583091	2.58855416	0.000767778	IPR005513 (PFAM)	P:GO:0009793
**Xvis03_223451-PA**	processed-gene-3.71-mRNA-1 protein Name:"Similar to CSP1 Cold shock protein 1 (Arabidopsis thaliana)" AED:0.30 eAED:0.30 QI:0|-1|0|1|-1|1|1|0|215	1.654539	2.16349653	0.000962503	IPR002059 (PFAM)	F:GO:0003676; F:GO:0008270
**Xvis03_207466-PA**	augustus-gene-21.70-mRNA-1 protein Name:"Similar to FLK Flowering locus K homology domain (Arabidopsis thaliana)" AED:0.22 eAED:0.22 QI:168|0.83|0.85|1|0.83|0.71|7|306|448	1.68075516	1.96005756	0.001155202		F:GO:0003723

**Xvis03_202655-PA**	processed-gene-53.13-mRNA-1 protein Name:"Similar to CCT2 Choline-phosphate cytidylyltransferase 2 (Arabidopsis thaliana)" AED:0.15 eAED:0.15 QI:50|1|1|1|1|1|8|392|291	0.98801174	2.43401057	0.001452068	IPR004821 (PFAM)	F:GO:0003824; P:GO:0009058
**Xvis03_208182-PA**	processed-gene-16.15-mRNA-1 protein Name:"Similar to EIF-5A2 Eukaryotic translation initiation factor 5A-2 (Nicotiana plumbaginifolia)" AED:0.11 eAED:0.11 QI:80|1|1|1|0.8|0.66|6|225|159	2.97866492	1.47675032	0.001520635	IPR001884	F:GO:0003723; F:GO:0003746; P:GO:0006452; F:GO:0043022; P:GO:0045901; P:GO:0045905
**Xvis03_220599-PA**	augustus-gene-1.77-mRNA-1 protein Name:"Similar to DHN1 Dehydrin DHN1 (Hordeum vulgare)" AED:0.00 eAED:0.00 QI:161|1|1|1|1|1|2|370|123	1.28815435	2.15460314	0.001607037	IPR000167 (PFAM)	P:GO:0009415
**Xvis03_207220-PA**	augustus-gene-29.99-mRNA-1 protein Name:"Similar to Bowman-Birk type trypsin inhibitor (Triticum aestivum)" AED:0.14 eAED:0.14 QI:36|1|1|1|1|1|2|205|120	1.35570052	2.18560715	0.001960747	IPR000877 (PFAM	F:GO:0004867; C:GO:0005576
**Xvis03_222487-PA**	processed-gene-2.59-mRNA-1 protein Name:"Similar to At4g22670 FAM10 family protein At4g22670 (Arabidopsis thaliana)" AED:0.11 eAED:0.11 QI:137|0.9|0.90|1|1|1|11|173|362	1.2118746	2.09622921	0.002077646	IPR034649 (PFAM)	F:GO:0005515; F:GO:0046983
**Xvis03_218117-PA**	augustus-gene-11.3-mRNA-1 protein Name:"Similar to Embryonic protein DC-8 (Daucus carota)" AED:0.04 eAED:0.04 QI:0|1|0.66|1|1|1|3|517|510	11.1566281	2.0199206	0.002120582		P:GO:0009737
**Xvis03_221354-PA**	augustus-gene-2.78-mRNA-1 protein Name:"Similar to RPS12 40S ribosomal protein S12 (Hordeum vulgare)" AED:0.26 eAED:0.26 QI:0|1|0.75|1|1|1|4|274|184	0.93357301	2.33749638	0.002343013	IPR004038 (PFAM)	F:GO:0003735; C:GO:0005840; P:GO:0006412
**Xvis03_207210-PA**	augustus-gene-28.48-mRNA-1 protein Name:"Similar to CLPB3 Chaperone protein ClpB3, mitochondrial (Oryza sativa subsp. japonica)" AED:0.12 eAED:0.12 QI:139|1|1|1|1|1|10|182|968	3.19122182	1.71878718	0.002414538	IPR003959 (PFAM)	F:GO:0005524; C:GO:0005737; P:GO:0009408; P:GO:0019538; P:GO:0042026
**Xvis03_213772-PA**	processed-gene-11.46-mRNA-1 protein Name:"Similar to MT4A Metallothionein-like protein 4A (Oryza sativa subsp.	0.6676822	2.38067769	0.002496221	IPR000347 (PFAM)	F:GO:0046872

	japonica)" AED:0.00 eAED:0.00 QI:173|1|1|1|1|1|2|379|80					
**Xvis03_215569-PA**	augustus-gene-5.91-mRNA-1 protein Name:"Similar to Carbonic anhydrase, chloroplastic (Hordeum vulgare)" AED:0.12 eAED:0.12 QI:674|1|1|1|1|1|8|205|260	12.65 2092 3	1.0636296 2	0.0026 88392	IPR0017 65 (PFAM	F:GO:0004089; F:GO:0008270
**Xvis03_216330-PA**	processed-gene-6.102-mRNA-1 protein Name:"Similar to PED1 3-ketoacyl-CoA thiolase 2, peroxisomal (Arabidopsis thaliana)" AED:0.14 eAED:0.14 QI:702|0.85|0.93|0.93|1|1|15|1904|479	2.142 1563 1	1.7195160 1	0.0030 16808	IPR0021 55	F:GO:0003824; F:GO:0016747
**Xvis03_200473-PA**	processed-gene-61.44-mRNA-1 protein Name:"Similar to HOP3 Hsp70-Hsp90 organizing protein 3 (Arabidopsis thaliana)" AED:0.09 eAED:0.09 QI:150|1|1|1|1|1|7|289|574	1.360 5723 6	2.1990004 6	0.0025 49607	IPR0119 90	F:GO:0005515
**Xvis03_212894-PA**	augustus-gene-2.1-mRNA-1 protein Name:"Similar to PORB Protochlorophyllide reductase B, chloroplastic (Hordeum vulgare)" AED:0.09 eAED:0.09 QI:162|1|1|1|1|1|5|457|393	0.863 6714 2	2.2836327 1	0.0026 17543	IPR0023 47	F:GO:0016630; P:GO:0055114
**Xvis03_219979-PA**	processed-gene-5.16-mRNA-1 protein Name:"Similar to GMPM1 18 kDa seed maturation protein (Glycine max)" AED:0.00 eAED:0.00 QI:97|1|1|1|1|1|2|370|156	2.192 7572 5	1.8584482 8	0.0035 30214	IPR0055 13 (PFAM)	P:GO:0009793
**Xvis03_223144-PA**	processed-gene-3.56-mRNA-1 protein Name:"Similar to RBCS1 Ribulose bisphosphate carboxylase small chain, chloroplastic (Musa	11.93 7209	0.8845895 1	0.0035 78017	IPR0246 81	no GO terms

	acuminata)" AED:0.05 eAED:0.05 QI:145|1|1|1|1|3|356|174					
**Xvis03_2212 3 6-PA**	augustus-gene-4.54-mRNA-1 protein Name:"Similar to PXG Peroxygenase (Oryza sativa subsp. japonica)" AED:0.38 eAED:0.38 QI:85|0.66|0.85|0.85|1|1|7|299|316	2.184 4627 7	1.6302500 3	0.0038 80978	IPR0077 36 (PFAM);	no GO terms
**Xvis03_2029 5 4-PA**	augustus-gene-28.13-mRNA-1 protein Name:"Similar to RPT1 26S protease regulatory subunit 7 (Prunus persica)" AED:0.05 eAED:0.05 QI:150|1|1|1|1|1|10|313|427	0.785 8625 2	2.2752088 1	0.0038 97195	IPR0415 69 (PFAM)	F:GO:0005524; C:GO:0005737; F:GO:0016787; P:GO:0030163
**Xvis03_2052 3 2-PA**	processed-gene-22.101-mRNA-1 protein Name:"Similar to DHN1 Dehydrin DHN1 (Hordeum vulgare)" AED:0.36 eAED:0.36 QI:115|1|1|1|1|1|2|350|132	0.748 8657	2.2392879 2	0.0041 90054	IPR0001 67 (PFAM)	P:GO:0009415
**Xvis03_2083 2 8-PA**	desiccation-related protein PCC13-62-like	14.19 7445 7	1.0339301	0.0042 3904	PF13668 (PFAM)	no GO terms
**Xvis03_2246 6 0-PA**	processed-gene-1.62-mRNA-1 protein Name:"Similar to FEN1 Flap endonuclease 1 (Zea mays)" AED:0.25 eAED:0.26 QI:20|0.7|0.71|0.95|1|1|21|362|621	0.790 0292 6	2.1774229 6	0.0046 13511	IPR0060 84 (PRINTS)	F:GO:0003735; F:GO:0004518; C:GO:0005840; P:GO:0006281; P:GO:0006412; F:GO:0016788
**Xvis03_2053 6 7-PA**	augustus-gene-34.43-mRNA-1 protein Name:"Similar to MTA Metallothionein-like protein 1 (Pisum sativum)" AED:0.00 eAED:0.00 QI:175|1|1|1|1|1|2|359|83	0.713 2959 4	2.1407999 9	0.0055 63873	IPR0003 47 (PFAM);	F:GO:0046872
**Xvis03_2190 5 5-PA**	augustus-gene-7.46-mRNA-1 protein Name:"Similar to TDX TPR repeat-containing thioredoxin TDX (Arabidopsis thaliana)" AED:0.04 eAED:0.04	3.129 9734 9	1.2722804 5	0.0062 35578	IPR0346 49 (PFAM)	F:GO:0005515; F:GO:0046983

	QI:262|1|1|1|1|1|11|486|390					
**Xvis03_2200 7 6-PA**	augustus-gene-4.88-mRNA-1 protein Name:"Similar to GDCSH Glycine cleavage system H protein, mitochondrial (Oryza sativa subsp. japonica)" AED:0.04 eAED:0.04 QI:127|1|1|1|1|1|4|484|165	1.846 7886 2	1.5457459 2	0.0063 44124	IPR0174 53	C:GO:0005960; P:GO:0019464
**Xvis03_2040 2 2-PA**	processed-gene-29.69-mRNA-1 protein Name:"Similar to RANBP1C Ran-binding protein 1 homolog c (Arabidopsis thaliana)" AED:0.01 eAED:0.01 QI:85|1|1|1|1|1|4|338|224	1.286 7017 4	1.8266814 7	0.0067 95094	IPR0001 56 (PFAM)	P:GO:0046907
**Xvis03_2165 1 5-PA**	augustus-gene-8.70-mRNA-1 protein Name:"Similar to SAPK7 Serine/threonine-protein kinase SAPK7 (Oryza sativa subsp. japonica)" AED:0.12 eAED:0.12 QI:115|0.83|1|1|1|1|7|226|305	1.408 9568 1	1.8659521 3	0.0070 1349	IPR0110 09	F:GO:0004672; F:GO:0005524; P:GO:0006468
**Xvis03_2055 1 2-PA**	processed-gene-10.28-mRNA- 1 protein Name:"Similar to Hsp83 Heat shock protein 83 (Drosophila melanogaster)" AED:0.12 eAED:0.12 QI:147|1|1|1|1|1|19|333|803	5.926 4619 8	1.0987413 5	0.0079 85264	IPR0205 75 (PRINTS	F:GO:0005524; P:GO:0006457; F:GO:0051082
**Xvis03_2154 3 4-PA**	augustus-gene-6.51-mRNA-1 protein Name:"Similar to Os01g0253300 Importin subunit alpha-1a (Oryza sativa subsp. japonica)" AED:0.21 eAED:0.21 QI:191|0.81|0.75|1|1|1|12|0|614	0.592 3643 7	2.0794711 8	0.0083 24319	IPR0119 89 (	F:GO:0005515; C:GO:0005634; C:GO:0005737; P:GO:0006606; F:GO:0008565; F:GO:0061608
**Xvis03_2250 4 2-PA**	augustus-gene-0.56-mRNA-1 protein Name:"Similar to CSD2 Superoxide dismutase [Cu-Zn] 2, chloroplastic (Arabidopsis thaliana)" AED:0.13 eAED:0.13 QI:217|0.85|0.87|1|1|1|8|225|22 6	10.89 4930 1	0.7706707 3	0.0085 11462	IPR0014 24 (PRINTS)	P:GO:0006801; F:GO:0046872

**Xvis03_2212 3 6-PA**	augustus-gene-4.54-mRNA-1 protein Name:"Similar to PXG Peroxygenase (Oryza sativa subsp. japonica)" AED:0.38 eAED:0.38 QI:85|0.66|0.85|0.85|1|1|7|299|316	2.138 1232 1	1.5111337	0.0093 16845	IPR0077 36 (PFAM)	no GO terms
**Xvis03_2248 8 7-PA**	augustus-gene-0.36-mRNA-1 protein Name:"Similar to At4g12130 Putative transferase At4g12130, mitochondrial (Arabidopsis thaliana)" AED:0.05 eAED:0.05 QI:50|1|1|1|1|1|5|427|411	1.726 6701	1.545695	0.0102 31088	IPR0177 03	F:GO:0005515
**Xvis03_2103 3 7-PA**	augustus-gene-8.1-mRNA-1 protein Name:"Similar to RPS12 40S ribosomal protein S12 (Hordeum vulgare)" AED:0.38 eAED:0.38 QI:0|1|0.75|1|1|1|4|393|161	0.788 9782 9	2.0046668 6	0.0104 36181	IPR0005 30 (PRINTS	F:GO:0003735; C:GO:0005840; P:GO:0006412
**Xvis03_2125 2 0-PA**	augustus-gene-6.14-mRNA-1 protein Name:"Similar to TUFB1 Elongation factor Tu, chloroplastic (Glycine max)" AED:0.04 eAED:0.16 QI:0|0|0.5|1|1|1|2|351|455	4.598 5812	0.9845974 2	0.0125 95747	IPR0007 95 (PRINTS)	F:GO:0003746; F:GO:0003924; F:GO:0005525; P:GO:0006414
**Xvis03_2197 8 4-PA**	processed-gene-6.0-mRNA-1 protein Name:"Similar to MBF1B Multiprotein-bridging factor 1b (Arabidopsis thaliana)" AED:0.06 eAED:0.06 QI:115|1|1|1|1|1|4|351|141	1.009 4991	1.8677196 4	0.0133 09481	IPR0137 29 (PFAM)	F:GO:0003677
**Xvis03_2027 8 4-PA**	processed-gene-9.6-mRNA-1 protein Name:"Similar to At5g12110 Elongation factor 1-beta 1 (Arabidopsis thaliana)" AED:0.07 eAED:0.07 QI:28|1|1|1|1|1|6|211|226	2.926 0132 7	1.1571813 6	0.0138 74725	IPR0147 17	F:GO:0003746; P:GO:0006414
**Xvis03_2148 7 1-PA**	augustus-gene-0.9-mRNA-1 protein Name:"Similar to CYS6 Cysteine proteinase inhibitor 6 (Arabidopsis thaliana)" AED:0.25 eAED:0.25	3.307 2269 5	1.0914322 6	0.0139 51824	IPR0000 10 (PFAM);	F:GO:0004869

	QI:34|0.2|0.33|1|0.8|0.83|6|0|2 3 7					
**Xvis03_2171 9 6-PA**	augustus-gene-1.26-mRNA-1 protein Name:"Similar to AKR2 Ankyrin repeat domain-containing protein 2 (Arabidopsis thaliana)" AED:0.11 eAED:0.11 QI:0|0|0|1|1|1|6|0|276	0.983 5682 7	1.7752949 7	0.0141 29423	IPR0206 83 (PFAM);	F:GO:0005515
**Xvis03_2151 5 3-PA**	augustus-gene-12.40-mRNA-1 protein Name:"Similar to RZ1B Glycine-rich RNA- binding protein RZ1B (Arabidopsis thaliana)" AED:0.25 eAED:0.25 QI:0|1|1|1|0.5|0.33|3|1599|326	0.738 4757 5	1.8385500 1	0.0176 2934	IPR0005 04 (PFAM);	F:GO:0003676; F:GO:0008270
**Xvis03_2182 0 0-PA**	augustus-gene-6.42-mRNA-1 protein Name:"Similar to PLP1 Patatin-like protein 1 (Oryza sativa subsp. japonica)" AED:0.26 eAED:0.26 QI:72|0.4|0.16|1|1|1|6|0|314	2.889 8699 1	1.3064709 9	0.0177 4307	IPR0026 41 (PFAM);	P:GO:0006629
**Xvis03_2255 3 2-PA**	processed-gene-0.13-mRNA-1 protein Name:"Similar to DHN3 Dehydrin DHN3 (Hordeum vulgare)" AED:0.00 eAED:0.00 QI:270|1|1|1|1|1|2|257|183	1.112 5766 4	1.5992062 4	0.0182 50186	IPR0001 67 (PFA	P:GO:0009415
**Xvis03_2029 2 9-PA**	augustus-gene-26.5-mRNA-1 protein Name:"Similar to RPL23A 60S ribosomal protein L23 (Arabidopsis thaliana)" AED:0.39 eAED:0.40 QI:0|0|0|0.5|1|1|6|0|279	2.398 3112 9	1.2000962 9	0.0182 86027	IPR0002 18 (PFAM)	F:GO:0003735; C:GO:0005840; P:GO:0006412
**Xvis03_2076 2 5-PA**	processed-gene-8.99-mRNA-1 protein Name:"Similar to COR47 Dehydrin COR47 (Arabidopsis thaliana)" AED:0.00 eAED:0.00 QI:100|1|1|1|1|1|2|435|268	1.896 6244 9	1.7208972 8	0.0186 44009	IPR0001 67 (PFAM)	P:GO:0009415
**Xvis03_2217 5 4-PA**	augustus-gene-1.62-mRNA-1 protein Name:"Similar to MORF1 Multiple organellar RNA editing factor 1, mitochondrial (Arabidopsis	0.936 6691 8	1.7289507 2	0.0187 5143	IPR0392 06 (PANTH ER);	P:GO:0016554

	thaliana)" AED:0.23 eAED:0.23 QI:47|1|1|1|0.75|0.6|5|384|406					
**Xvis03_2052 3 2-PA**	processed-gene-22.101-mRNA-1 protein Name:"Similar to DHN1 Dehydrin DHN1 (Hordeum vulgare)" AED:0.36 eAED:0.36 QI:115|1|1|1|1|1|2|350|132	1.467 1627 6	1.5839294 8	0.0189 02918	IPR0001 67 (PFAM)	P:GO:0009415
**Xvis03_2181 5 8-PA**	augustus-gene-3.24-mRNA-1 protein Name:"Similar to Glutaredoxin (Ricinus communis)" AED:0.01 eAED:0.01 QI:220|1|1|1|1|1|4|501|106	2.822 1512 7	1.1671811 2	0.0194 40464	IPR0140 25	F:GO:0009055; F:GO:0015035; P:GO:0045454
**Xvis03_2002 5 0-PA**	processed-gene-30.2-mRNA-1 protein Name:"Similar to HSP70 Stromal 70 kDa heat shock-related protein, chloroplastic (Pisum sativum)" AED:0.12 eAED:0.12 QI:46|0.75|0.88|0.88|1|1|9|437|741	19.81 3914	0.5548722 7	0.0209 00757	IPR0131 26 (PRINTS)	F:GO:0005524; P:GO:0006457; F:GO:0051082
**Xvis03_2056 9 1-PA**	processed-gene-31.124-mRNA-1 protein Name:"Similar to RuBisCO large subunit-binding protein subunit beta, chloroplastic (Pisum sativum)" AED:0.20 eAED:0.20 QI:97|0.8|0.81|0.87|0.93|0.87|16|0|678	13.01 9614	0.6372995 8	0.0209 57446	IPR0018 44	F:GO:0005524; P:GO:0042026
**Xvis03_2064 5 8-PA**	processed-gene-30.78-mRNA- 1 protein Name:"Similar to PCKR1 Peptidyl-prolyl cis-trans isomerase (Catharanthus roseus)" AED:0.01 eAED:0.01 QI:0|-1|0|1|-1|1|1|0|173	4.553 7958 2	0.8864205 6	0.0234 936	IPR0021 30	P:GO:0000413; F:GO:0003755

**Xvis03_2098 7 6-PA**	augustus-gene-14.5-mRNA-1 protein Name:"Similar to RPS27B 40S ribosomal protein S27-2 (Arabidopsis thaliana)" AED:0.17 eAED:0.17 QI:86|1|1|1|0.66|0.75|4|47|86	0.779 0348 2	1.7654785 2	0.0216 19681	IPR0005 92 (PFAM	F:GO:0003735; C:GO:0005840; P:GO:0006412
**Xvis03_2021 0 3-PA**	processed-gene-9.9-mRNA-1 protein Name:"Similar to Os02g0639900 Thioredoxin M1, chloroplastic (Oryza sativa subsp. japonica)" AED:0.00 eAED:0.00 QI:100|1|1|1|1|1|2|1208|190	1.754 2165 8	1.3518750 5	0.0216 60558	PR00421	P:GO:0006662; F:GO:0015035; P:GO:0045454
**Xvis03_2212 5 2-PA**	augustus-gene-6.27-mRNA-1 protein Name:"Similar to SPG20 Spartin (Homo sapiens)" AED:0.04 eAED:0.04 QI:93|1|1|1|0.75|0.6|5|21|403	4.900 4118 4	0.9144375 7	0.0235 04246	IPR0096 86 (PFAM)	no GO terms
**Xvis03_2125 2 0-PA**	augustus-gene-6.14-mRNA-1 protein Name:"Similar to TUFB1 Elongation factor Tu, chloroplastic (Glycine max)" AED:0.04 eAED:0.16 QI:0|0|0.5|1|1|1|2|351|455	6.587 0721 9	0.7929123	0.0280 19252	IPR0007 95	F:GO:0003746; F:GO:0003924; F:GO:0005525; P:GO:0006414
**Xvis03_2114 2 4-PA**	augustus-gene-2.30-mRNA-1 protein Name:"Similar to GLX2-2 Hydroxyacylglutathione hydrolase cytoplasmic (Arabidopsis thaliana)" AED:0.23 eAED:0.23 QI:367|1|1|1|1|0.83|6|257|190	0.783 7434 4	1.6639410 2	0.0286 71952	IPR0322 82 (PFAM)	F:GO:0004416; P:GO:0019243
**Xvis03_2218 8 3-PA**	processed-gene-4.16-mRNA-1 protein Name:"Similar to HVA22A HVA22-like protein a (Arabidopsis thaliana)" AED:0.26 eAED:0.26 QI:148|0.57|0.5|0.87|0.85|0.75|8|368|228	0.456 4551 3	1.7182068 5	0.0293 54802	IPR0043 45 (PANTH ER)	no GO terms
**Xvis03_2175 8 5-PA**	augustus-gene-6.44-mRNA-1 protein Name:"Similar to PAB8 Polyadenylate-binding protein 8 (Arabidopsis thaliana)"	1.743 5770 4	1.2466561 2	0.0295 18147	IPR012 6 77	F:GO:0003676; F:GO:0003723

	AED:0.09 eAED:0.09 QI:312|0.75|0.66|1|1|1|9|460|63 4					
**Xvis03_2095 4 8-PA**	processed-gene-8.27-mRNA-1 protein Name:"Similar to AN3 Naringenin,2-oxoglutarate 3-dioxygenase (Fragment) (Petunia hybrida)" AED:0.02 eAED:0.02 QI:65|1|1|1|1|1|3|192|370	0.883 5437 7	1.6641330 9	0.0306 7983	IPR027 4 43	F:GO:0016491; P:GO:0055114
**Xvis03_2161 3 5-PA**	augustus-gene-2.5-mRNA-1 protein Name:"Similar to GSA Glutamate-1-semialdehyde 2,1-aminomutase, chloroplastic (Oryza sativa subsp. japonica)" AED:0.00 eAED:0.00 QI:90|1|1|1|1|1|3|296|478	3.393 3298 2	0.9368866 8	0.0331 05684	IPR004 6 39	F:GO:0003824; F:GO:0008483; F:GO:0030170; P:GO:0033014; F:GO:0042286
**Xvis03_2025 6 2-PA**	augustus-gene-46.31-mRNA-1 protein Name:"Similar to Major pollen allergen Cor a 1 isoforms 5, 6, 11 and 16 (Corylus avellana)" AED:0.26 eAED:0.26 QI:0|1|0.5|1|1|1|2|234|182	2.336 4062 8	1.2451940 2	0.0343 20067	IPR000 9 16 (PFAM)	P:GO:0006952
**Xvis03_2157 5 9-PA**	augustus-gene-6.67-mRNA-1 protein Name:"Similar to At5g39570 Uncharacterized protein At5g39570 (Arabidopsis thaliana)" AED:0.00 eAED:0.00 QI:88|1|1|1|1|1|2|401|452	0.884 6234 4	1.6552063 4	0.0352 91239	IPR038 9 43 (PANT H ER)	F:GO:0070300
**Xvis03_2238 6 0-PA**	processed-gene-2.57-mRNA-1 protein Name:"Similar to v1g171563 Eukaryotic translation initiation factor 3 subunit G (Nematostella vectensis)" AED:0.02 eAED:0.02 QI:0|1|0.5|1|1|1|2|3401|289	1.202 2400 2	1.3756846 3	0.0373 58344	IPR024 6 75 (PFAM)	F:GO:0003676; F:GO:0003743; C:GO:0005737; C:GO:0005852

**Xvis03_2247 5 4-PA**	processed-gene-1.55-mRNA-1 protein Name:"Similar to At3g10130 Heme-binding-like protein At3g10130, chloroplastic (Arabidopsis thaliana)" AED:0.02 eAED:0.02 QI:0|-1|0|1|-1|1|1|0|209	0.556 5459 9	1.6212252 7	0.0397 11403	IPR006 9 17 (PFAM)	no GO terms
**Xvis03_2207 1 9-PA**	augustus-gene-6.9-mRNA-1 protein Name:"Similar to TSJT1 Stem-specific protein TSJT1 (Nicotiana tabacum)" AED:0.07 eAED:0.07 QI:59|0.5|0.8|0.8|1|1|5|232|256	0.527 9493 4	1.6116065 9	0.0412 78577	IPR029 0 55	no GO terms
**Xvis03_2081 4 0-PA**	processed-gene-11.6-mRNA-1 protein Name:"Similar to atad1a ATPase family AAA domain-containing protein 1-A (Danio rerio)" AED:0.12 eAED:0.12 QI:153|0.88|0.94|0.94|0.83|0.78|19|436|852	0.458 6671 1	1.5705785 9	0.0417 79846	IPR003 9 59 (PFAM)	F:GO:0005524
**Xvis03_21576 4-PA**	augustus-gene-6.61-mRNA-1 protein Name:"Similar to 29 kDa ribonucleoprotein B, chloroplastic (Nicotiana sylvestris)" AED:0.25 eAED:0.25 QI:94|0.75|0.6|0.8|1|1|5|0|355	3.073 1014 8	0.9518617 3	0.0427 28409	IPR0126 77	F:GO:0003676
**Xvis03_20141 6-PA**	processed-gene-12.20-mRNA-1 protein Name:"Similar to Phosphoglycerate kinase, chloroplastic (Nicotiana tabacum)" AED:0.04 eAED:0.04 QI:190|1|1|1|1|1|6|289|477	21.54 8955 5	0.4267875	0.0432 73487	IPR0015 76	F:GO:0004618; P:GO:0006096
**Xvis03_22054 9-PA**	processed-gene-6.81-mRNA-1 protein Name:"Similar to NFS2 Cysteine desulfurase 1, chloroplastic (Arabidopsis thaliana)" AED:0.13 eAED:0.23 QI:55|0.77|0.8|1|1|1|10|0|498	0.521 1484 4	1.5881456 9	0.0439 76028	IPR0154 21	F:GO:0003824; P:GO:0006534; F:GO:0030170; F:GO:0031071
**Xvis03_20314 0-PA**	processed-gene-45.24-mRNA-1 protein Name:"Similar to HSP81-1 Heat shock protein 81-1 (Oryza sativa subsp. japonica)" AED:0.48 eAED:0.48 QI:0|-1|0|1|-1|1|1|0|114	0.541 9099 6	1.5849319	0.0443 55192	IPR0014 04 (PFAM)	F:GO:0005524; P:GO:0006457; F:GO:0051082

**Xvis03_206453-PA**	processed-gene-29.128-mRNA-1 protein Name:"Similar to CSP4 Cold shock domain-containing protein 4 (Arabidopsis thaliana)" AED:0.32 eAED:0.32 QI:0|-1|0|1|-1|1|1|0|231	1.3646643	1.28219745	0.044671688	PR01228	F:GO:0003676; F:GO:0008270
**Xvis03_219977-PA**	augustus-gene-5.85-mRNA-1 protein Name:"Similar to Os02g0194200 Zinc finger CCCH domain-containing protein 14 (Oryza sativa subsp. japonica)" AED:0.00 eAED:0.00 QI:185|1|1|1|1|1|3|411|290	0.56026254	1.55102977	0.045328627	IPR036612	F:GO:0003723; F:GO:0046872
**Xvis03_222778-PA**	processed-gene-2.13-mRNA-1 protein Name:"Similar to PR1 Pathogenesis-related protein 1 (Asparagus officinalis)" AED:0.28 eAED:0.28 QI:65|1|1|1|1|1|2|141|157	1.87050016	1.17876116	0.046245264	IPR000916 (PFAM)	P:GO:0006952
**Xvis03_205594-PA**	augustus-gene-17.68-mRNA-1 protein Name:"Similar to RPS27B 40S ribosomal protein S27-2 (Arabidopsis thaliana)" AED:0.09 eAED:0.09 QI:0|1|0.75|1|1|1|4|342|163	0.98468332	1.40368325	0.048067861	IPR000592 (PFAM)	F:GO:0003735; C:GO:0005840; P:GO:0006412
**Xvis03_207368-PA**	processed-gene-12.44-mRNA-1 protein Name:"Similar to Polyphenol oxidase, chloroplastic (Malus domestica)" AED:0.07 eAED:0.08 QI:0|-1|0|1|-1|1|1|0|578	8.36580698	0.72935219	0.049840164	IPR002227 (PFAM)	F:GO:0004097; F:GO:0016491; P:GO:0046148; P:GO:0055114
**Xvis03_211532-PA**	est_gff_Cufflinks-gene-11.15-mRNA-1 protein Name:"Similar to Glucan endo-1,3-beta-glucosidase, acidic isoform (Zea mays)" AED:0.00 eAED:0.00 QI:59|1|1|1|1|1|2|102|335	3.15999	-2.188	0.00389	IPR000490 (PFAM)	
**Xvis03_212894- PA**	augustus-gene-2.1-mRNA-1 protein Name:"Similar to PORB Protochlorophyllide reductase B, chloroplastic (Hordeum vulgare)" AED:0.09 eAED:0.09 QI:162|1|1|1|1|1|5|457|393	3.05149	- 1.8088	0.00687	IPR002347 (PFAM	
**Xvis03_213363- PA**	processed-gene-12.66-mRNA-1 protein Name:"Similar to Os09g0529100 Probable 6-phosphogluconolactonase 4, chloroplastic (Oryza sativa subsp. japonica)" AED:0.04	0.91926	- 1.9052	0.00974	IPR006148 (PFAM	F:GO:0004553; P:GO:0005975

eAED:0.04 QI:89|1|1|1|1|3|259|276

**Xvis03_224897- PA**	augustus-gene-1.57-mRNA-1 protein Name:"Similar to HSP90 Endoplasmin homolog (Catharanthus roseus)" AED:0.10 eAED:0.10 QI:52|0.92|0.93|1|1|1|15|395|788	4.50708	- 1.1669	0.00987	IPR003594 (PFAM) ;	F:GO:0016630; P:GO:0055114
**Xvis03_224459- PA**	augustus-gene-1.13-mRNA-1 protein Name:"Similar to RABA2A Ras- related protein RABA2a (Arabidopsis thaliana)" AED:0.02 eAED:0.02 QI:202|1|1|1|1|1|2|509|220	1.26609	- 1.6274	0.01404	IPR001806 (PFAM)	P:GO:0005975; P:GO:0006098; F:GO:0017057
**Xvis03_207311- PA**	processed-gene-7.62-mRNA-1 protein Name:"Similar to ADK-B Adenylate kinase 4 (Oryza sativa subsp. japonica)" AED:0.05 eAED:0.05 QI:122|1|1|1|1|1|6|379|246	2.07578	- 1.4418	0.01502	IPR007862 (PFAM)	F:GO:0005524; P:GO:0006457; F:GO:0051082
**Xvis03_207665- PA**	processed-gene-12.71-mRNA-1 protein Name:"Similar to HSP90 Endoplasmin homolog (Catharanthus roseus)" AED:0.12 eAED:0.12 QI:42|1|1|1|1|1|15|358|812	3.28464	- 1.2209	0.01548	IPR003594 (PFAM	F:GO:0003924; F:GO:0005525
**Xvis03_209992- PA**	processed-gene-2.103-mRNA-1 protein Name:"Similar to Os06g0675700 Probable alpha-glucosidase Os06g0675700 (Oryza sativa subsp. japonica)" AED:0.08 eAED:0.08 QI:226|1|1|1|1|1|5|499|873	2.49202	- 1.2614	0.01656	IPR025887 (PFAM)	F:GO:0004017; F:GO:0005524; P:GO:0006139; F:GO:0016776; F:GO:0019205
**Xvis03_210350- PA**	processed-gene-9.1-mRNA-1 protein Name:"Similar to TP53I3 Quinone oxidoreductase PIG3 (Homo sapiens)" AED:0.11 eAED:0.11 QI:0|0.44|0.5|0.7|1|1|10|268|486	1.93408	- 1.5504	0.01721	IPR013149 (PFAM)	F:GO:0005524; P:GO:0006457; F:GO:0051082

**Xvis03_216387- PA**	augustus-gene-11.65-mRNA-1 protein Name:"Similar to Alpha-galactosidase (Coffea arabica)" AED:0.16 eAED:0.16 QI:81|1|1|1|0.92|0.92|14|139|642	3.48955	- 1.0714	0.01845	IPR041233 (PFAM)	F:GO:0003824; F:GO:0004553; P:GO:0005975; F:GO:0030246
**Xvis03_216480- PA**	augustus-gene-6.109-mRNA-1 protein Name:"Similar to UVR8 Ultraviolet-B receptor UVR8 (Arabidopsis thaliana)" AED:0.10 eAED:0.10 QI:223|0.9|1|1|1|1|11|290|403	1.25457	- 1.5671	0.02033	IPR000408	P:GO:0055114
**Xvis03_207311- PA**	processed-gene-7.62-mRNA-1 protein Name:"Similar to ADK-B Adenylate kinase 4 (Oryza sativa subsp. japonica)" AED:0.05 eAED:0.05 QI:122|1|1|1|1|1|6|379|246	2.07474	- 1.3597	0.02359	IPR007862 (PFAM	F:GO:0003824; F:GO:0004553; P:GO:0005975
**Xvis03_203608- PA**	processed-gene-32.69-mRNA-1 protein Name:"Similar to PNSL1 Photosynthetic NDH subunit of lumenal location 1, chloroplastic (Arabidopsis thaliana)" AED:0.12 eAED:0.08 QI:81|1|0.85|1|1|1|7|565|263	0.92383	- 1.6288	0.02519	IPR002683 (PFAM);	F:GO:0009881; P:GO:0010224; F:GO:0042803
**Xvis03_206138- PA**	processed-gene-3.62-mRNA-1 protein Name:"Similar to CHI4 Endochitinase PR4 (Phaseolus vulgaris)" AED:0.03 eAED:0.03 QI:30|1|1|1|1|1|2|158|277	1.24683	- 1.7023	0.02712	IPR001002 (PFAM)	F:GO:0004017; F:GO:0005524; P:GO:0006139; F:GO:0016776; F:GO:0019205
**Xvis03_219185- PA**	processed-gene-8.51-mRNA-1 protein Name:"Similar to tal Transaldolase (Acidothermus cellulolyticus (strain ATCC 43068 / 11B))" AED:0.03 eAED:0.03 QI:60|0.83|0.85|1|1|1|7|483|446	0.54474	- 1.7355	0.02777	IPR001585 (PFAM)	F:GO:0005509; C:GO:0009523; C:GO:0009654; P:GO:0015979; C:GO:0019898
**Xvis03_202672- PA**	processed-gene-54.103-mRNA-1 protein Name:"Similar to HEMC Porphobilinogen deaminase, chloroplastic (Oryza sativa	3.14776	-1.086	0.0314	IPR022418 (PFAM)	F:GO:0004568; P:GO:0005975; P:GO:0006032; F:GO:0008061; P:GO:0016998

	subsp. japonica)" AED:0.01 eAED:0.01 QI:117|1|1|1|1|1|5|327|378					
**Xvis03_203714- PA**	processed-gene-43.103-mRNA-1 protein Name:"Similar to At4g26910 Dihydrolipoyllysine-residue succinyltransferase component of 2- oxoglutarate dehydrogenase complex 2, mitochondrial (Arabidopsis thaliana)" AED:0.18 eAED:0.18 QI:18|1|1|1|0.85|0.8|15|384|475	4.416 19	- 0.894 4	0.0325 4	IPR02 0828 (PFA M)	F:GO:0003824; F:GO:0004801; C:GO:0005737; P:GO:0005975; P:GO:0006098
**Xvis03_207856- PA**	augustus-gene-3.47-mRNA-1 protein Name:"Similar to GAPC2 Glyceraldehyde-3-phosphate dehydrogenase 2, cytosolic (Oryza sativa subsp. japonica)" AED:0.71 eAED:0.72 QI:0|0|0|0.66|1|1|6|0|224	7.893 22	-0.733	0.0328 8	IPR02 0828 (PFA M)	F:GO:0004418; P:GO:0033014
**Xvis03_200416- PA**	processed-gene-51.69-mRNA-1 protein Name:"Similar to CHLM Magnesium protoporphyrin IX methyltransferase, chloroplastic (Arabidopsis thaliana)" AED:0.34 eAED:0.34 QI:0|-1|0|1|-1|1|1|0|322	1.284 94	- 1.548 5	0.0377 6	); IPR0109 40 (PFAM	F:GO:0004149; P:GO:0006099; F:GO:0016620; F:GO:0016746; C:GO:0045252; P:GO:0055114
**Xvis03_220112- PA**	augustus-gene-8.33-mRNA-1 protein Name:"Similar to RPL5 50S ribosomal protein L5, chloroplastic (Arabidopsis thaliana)" AED:0.02 eAED:0.03 QI:29|0.5|0.33|1|1|1|3|0|284	3.421 34	- 0.937 8	0.0414 9	IPR03 1309 (PFA M)	F:GO:0016620; P:GO:0055114
**Xvis03_215774- PA**	augustus-gene-7.23-mRNA-1 protein Name:"Similar to CHLP Geranylgeranyl diphosphate reductase, chloroplastic (Nicotiana tabacum)" AED:0.00 eAED:0.00 QI:382|1|1|1|1|1|2|212|364	0.901 09	- 1.542 3	0.0443 7	PTHR42 685 (PANTH ER)	P:GO:0015995; F:GO:0046406

**Xvis03_21360 5- PA**	processed-gene-14.84-mRNA-1 protein Name:"Similar to Os08g0288200 Probable adenylate kinase 5, chloroplastic (Oryza sativa subsp. japonica)" AED:0.22 eAED:0.22 QI:25|1|0.94|1|1|1|17|0|611	0.701 86	- 1.537 1	0.0450 6	IPR01 8962 (PFA M)	F:GO:0003735; C:GO:0005840; P:GO:0006412
**Xvis03_21892 7- PA**	processed-gene-4.16-mRNA-1 protein Name:"Similar to PORB Protochlorophyllide reductase B, chloroplastic (Hordeum vulgare)" AED:0.12 eAED:0.12 QI:128|0.75|0.6|1|1|1|5|341|353	2.115 55	- 1.344 3	0.0478 4	IPR00 2347 (PFA M)	P:GO:0015979; P:GO:0015995; F:GO:0045550; P:GO:0051188; P:GO:0055114
**Xvis03_21205 2- PA**	augustus-gene-18.3-mRNA-1 protein Name:"Similar to RPN8A 26S proteasome non-ATPase regulatory subunit 7 homolog A (Arabidopsis thaliana)" AED:0.29 eAED:0.29 QI:0|0.9|0.81|0.90|1|1|11|444|363	0.879 62	- 1.475 1	0.0482 2	IPR024 969 (PFAM)	F:GO:0004017; F:GO:0005524; P:GO:0006139; F:GO:0016776; F:GO:0019205
**Xvis03_21126 8- PA**	processed-gene-9.25-mRNA-1 protein Name:"Similar to Vacuolar- processing enzyme (Citrus sinensis)" AED:0.07 eAED:0.07 QI:3|1|1|1|1|1|9|417|590	0.810 8	- 1.481 1	0.0488 9	IPR001096	F:GO:0016630; P:GO:0055114
**Xvis03_22309 5- PA**	augustus-gene-3.96-mRNA-1 protein Name:"Similar to petC Cytochrome b6-f complex iron-sulfur subunit, chloroplastic (Oryza sativa subsp. japonica)" AED:0.11 eAED:0.11 QI:167|0.33|0.75|1|1|1|4|287|178	1.792 06	- 1.193 4	0.0491 3	IPR005805	F:GO:0005515; C:GO:0005838
**Xvis03_21211 3- PA**	augustus-gene-6.61-mRNA-1 protein Name:"Similar to GGAT1 Glutamate--glyoxylate aminotransferase 1 (Arabidopsis thaliana)" AED:0.19 eAED:0.19 QI:107|0.85|0.93|0.93|0.85|0.93|15|45 0|533	3.887 92	- 2.439 4	4.44E-05	IPR004 839 (PFAM) ;	P:GO:0006508; F:GO:0008233

**Xvis03_219859- PA**	augustus-gene-3.73-mRNA-1 protein Name:"Similar to PSBO Oxygen- evolving enhancer protein 1, chloroplastic (Solanum lycopersicum)" AED:0.03 eAED:0.03 QI:216|1|1|1|1|1|2|224|332	18.7878	- 1.2783	9.28E-05	IPR002628 (PFAM) ;	F:GO:0008121; F:GO:0009496; C:GO:0009507; C:GO:0016020; F:GO:0016491; F:GO:0016679; C:GO:0042651; F:GO:0045158; F:GO:0051537; P:GO:0055114
**Xvis03_223853- PA**	processed-gene-1.15-mRNA-1 protein Name:"Similar to RCA2 Ribulose bisphosphate carboxylase/oxygenase activase 2, chloroplastic (Larrea tridentata)" AED:0.12 eAED:0.12 QI:111|1|1|1|0.83|0.71|7|332|438	18.4585	-1.198	0.00026	IPR003959 (PFAM) ;	F:GO:0003824; P:GO:0009058; F:GO:0030170
**Xvis03_204705- PA**	processed-gene-11.1-mRNA-1 protein Name:"Similar to RPS17 40S ribosomal protein S17 (Solanum lycopersicum)" AED:0.06 eAED:0.06 QI:0|-1|0|1|-1|1|1|0|143	2.53029	- 2.2506	0.0004	IPR001210 (PFAM) ;	C:GO:0009654; P:GO:0010207; F:GO:0010242; P:GO:0042549
**Xvis03_214214- PA**	processed-gene-11.33-mRNA-1 protein Name:"Similar to APX1 L- ascorbate peroxidase, cytosolic (Pisum sativum)" AED:0.17 eAED:0.17 QI:131|1|1|1|1|1|7|558|249	2.83113	- 2.0161	0.00052	IPR002016	F:GO:0005524
**Xvis03_223077- PA**	processed-gene-1.89-mRNA-1 protein Name:"Similar to CAB13 Chlorophyll a-b binding protein 13, chloroplastic (Solanum lycopersicum)" AED:0.07 eAED:0.07 QI:182|1|1|1|1|1|4|188|263	2.97724	- 1.9812	0.00056	IPR022796 (PFAM) ;	F:GO:0003735; C:GO:0005840; P:GO:0006412
**Xvis03_223853- PA**	processed-gene-1.15-mRNA-1 protein Name:"Similar to RCA2 Ribulose bisphosphate carboxylase/oxygenase activase 2, chloroplastic (Larrea tridentata)" AED:0.12 eAED:0.12 QI:111|1|1|1|0.83|0.71|7|332|438	8.529	- 1.4157	0.00061		F:GO:0004601; P:GO:0006979; F:GO:0020037; P:GO:0055114

**Xvis03_204705- PA**	processed-gene-11.1-mRNA-1 protein Name:"Similar to RPS17 40S ribosomal protein S17 (Solanum lycopersicum)" AED:0.06 eAED:0.06 QI:0|-1|0|1|-1|1|1|0|143	2.4569	- 2.1525	0.00069	IPR001210 (PFAM);	P:GO:0009765; C:GO:0016020
**Xvis03_220112- PA**	augustus-gene-8.33-mRNA-1 protein Name:"Similar to RPL5 50S ribosomal protein L5, chloroplastic (Arabidopsis thaliana)" AED:0.02 eAED:0.03 QI:29|0.5|0.33|1|1|1|3|0|284	3.42134	- 1.8424	0.00091	IPR031309 (PFAM)	F:GO:0005524
**Xvis03_208337- PA**	augustus-gene-6.16-mRNA-1 protein Name:"Similar to PSBO Oxygen- evolving enhancer protein 1, chloroplastic (Solanum lycopersicum)" AED:0.08 eAED:0.08 QI:229|1|1|1|0.5|0.33|3|86|332	20.0766	- 1.0303	0.00095		F:GO:0003735; C:GO:0005840; P:GO:0006412
**Xvis03_209988- PA**	processed-gene-2.101-mRNA-1 protein Name:"Similar to PSBP Oxygen-evolving enhancer protein 2, chloroplastic (Cucumis sativus)" AED:0.32 eAED:0.32 QI:142|1|1|1|1|1|4|296|262	8.30561	- 1.3232	0.00099	IPR002683 (PFAM);	F:GO:0003735; C:GO:0005840; P:GO:0006412
**Xvis03_211060- PA**	processed-gene-14.7-mRNA-1 protein Name:"Similar to Chlorophyll a-b binding protein, chloroplastic (Spinacia oleracea)" AED:0.01 eAED:0.01 QI:0|-1|0|1|-1|1|1|0|268	3.49088	- 1.5912	0.00166	IPR023329	C:GO:0009654; P:GO:0010207; F:GO:0010242; P:GO:0042549
**Xvis03_209992- PA**	processed-gene-2.103-mRNA-1 protein Name:"Similar to Os06g0675700 Probable alpha-glucosidase Os06g0675700 (Oryza sativa subsp. japonica)" AED:0.08 eAED:0.08 QI:226|1|1|1|1|1|5|499|873	2.49202	- 1.8609	0.00168	IPR025887 (PFAM)	F:GO:0005509; C:GO:0009523; C:GO:0009654; P:GO:0015979; C:GO:0019898
**Xvis03_222105- PA**	augustus-gene-5.53-mRNA-1 protein Name:"Similar to APXT L-ascorbate peroxidase T,	1.93413	- 2.1494	0.00227	IPR002016	P:GO:0009765; C:GO:0016020

	chloroplastic (Arabidopsis thaliana)" AED:0.32 eAED:0.32 QI:0|0.5|0.45|0.72|0.9|0.81|11|0|364					
**Xvis03_219178- PA**	processed-gene-7.19-mRNA-1 protein Name:"Similar to CNX1 Calnexin homolog 1 (Arabidopsis thaliana)" AED:0.09 eAED:0.09 QI:144|1|1|1|0.83|0.71|7|378|540	5.205 08	- 1.579 1	0.0023 6	IPR001580	F:GO:0003824; F:GO:0004553; P:GO:0005975; F:GO:0030246
**Xvis03_223899- PA**	processed-gene-1.98-mRNA-1 protein Name:"Similar to RCA1 Ribulose bisphosphate carboxylase/oxygenase activase, chloroplastic (Zea mays)" AED:0.16 eAED:0.16 QI:463|0.90|0.91|1|0.81|0.66|12|2 25|2 80	20.31	- 0.901 6	0.0030 5	IPR003 959 (PFAM);	F:GO:0004601; P:GO:0006979; F:GO:0020037; P:GO:0055114
**Xvis03_201883- PA**	augustus-gene-50.73-mRNA-1 protein Name:"Similar to APX1 L- ascorbate peroxidase, cytosolic (Pisum sativum)" AED:0.15 eAED:0.15 QI:0|0.85|0.75|1|1|0.87|8|414|307	2.256 91	- 1.834 6	0.0031 2	IPR002 207 (PRINT S);	F:GO:0005509; F:GO:0005515; C:GO:0005783; P:GO:0006457; F:GO:0051082
**Xvis03_206991- PA**	augustus-gene-10.52-mRNA-1 protein Name:"Similar to CPN21 20 kDa chaperonin, chloroplastic (Arabidopsis thaliana)" AED:0.07 eAED:0.07 QI:99|1|1|1|1|1|5|439|254	5.803 16	- 1.302 9	0.0033 7	IPR020818	F:GO:0005524

**Xvis03_207825-PA**	processed-gene-26.31-mRNA-1 protein Name:"Similar to RPS17 40S ribosomal protein S17 (Solanum lycopersicum)" AED:0.03 eAED:0.03 QI:0|-1|0|1|-1|1|1|0|146	3.87617	-1.5202	0.00355	IPR036401	F:GO:0004601; P:GO:0006979; F:GO:0020037; P:GO:0055114
**Xvis03_213217-PA**	augustus-gene-16.1-mRNA-1 protein Name:"Similar to CSP41A Chloroplast stem-loop binding protein of 41 kDa a, chloroplastic (Arabidopsis thaliana)" AED:0.01 eAED:0.01 QI:41|1|1|1|1|1|6|52|409	9.28885	-1.198	0.00395		P:GO:0006457; F:GO:0046914; P:GO:1901671
**Xvis03_221799-PA**	processed-gene-5.86-mRNA-1 protein Name:"Similar to LHCB5 Chlorophyll a-b binding protein CP26, chloroplastic (Arabidopsis thaliana)" AED:0.06 eAED:0.06 QI:123|0.8|0.83|1|1|1|6|161|279	3.70776	-1.4652	0.00433	IPR022796 (PFAM);	F:GO:0003735; C:GO:0005840; P:GO:0006412
**Xvis03_225066-PA**	processed-gene-0.82-mRNA-1 protein Name:"Similar to Chlorophyll a-b binding protein, chloroplastic (Spinacia oleracea)" AED:0.00 eAED:0.00 QI:0|-1|0|1|-1|1|1|0|268	4.2432	-1.3049	0.00457	IPR022796 (PFAM);	F:GO:0003824; F:GO:0050662
**Xvis03_203608-PA**	processed-gene-32.69-mRNA-1 protein Name:"Similar to PNSL1 Photosynthetic NDH subunit of lumenal location 1, chloroplastic (Arabidopsis thaliana)" AED:0.12 eAED:0.08 QI:81|1|0.85|1|1|1|7|565|263	0.92383	-2.0582	0.0052	IPR002683 (PFAM);	P:GO:0009765; C:GO:0016020
**Xvis03_218527-PA**	augustus-gene-7.89-mRNA-1 protein Name:"Similar to FBPban1 Fructose-1,6-bisphosphatase, cytosolic (Musa acuminata)" AED:0.29 eAED:0.29 QI:151|0.63|0.75|1|0.54|0.66|12|242|350	2.84907	-1.5592	0.00612	IPR028343	P:GO:0009765; C:GO:0016020
**Xvis03_224654-PA**	processed-gene-1.9-mRNA-1 protein Name:"Similar to CAB21 Chlorophyll a-b binding protein 21, chloroplastic (Nicotiana tabacum)" AED:0.11 eAED:0.11 QI:0|0|0|0.5|1|1|2|0|328	2.58311	-1.5193	0.00684	IPR023329	F:GO:0005509; C:GO:0009523; C:GO:0009654; P:GO:0015979; C:GO:0019898
**Xvis03_210597-PA**	augustus-gene-8.18-mRNA-1 protein Name:"Similar to At1g67280 Probable lactoylglutathione lyase, chloroplast (Arabidopsis thaliana)" AED:0.06 eAED:0.06 QI:224|1|1|1|1|1|9|419|372	2.83797	-1.597	0.00703	IPR029068	P:GO:0005975; F:GO:0016791; F:GO:0042132
**Xvis03_213652-PA**	processed-gene-16.266-mRNA-1 protein Name:"Similar to RPL4 50S ribosomal protein L4, chloroplastic (Nicotiana tabacum)" AED:0.18 eAED:0.18 QI:0|-1|0|1|-1|1|1|0|272	2.15786	-1.7564	0.00707	IPR013005	P:GO:0009765; C:GO:0016020
**Xvis03_202672-PA**	processed-gene-54.103-mRNA-1 protein Name:"Similar to HEMC Porphobilinogen deaminase, chloroplastic (Oryza sativa subsp. japonica)" AED:0.01 eAED:0.01 QI:117|1|1|1|1|1|5|327|378	3.14577	-1.4667	0.00736	IPR000860	F:GO:0004462; F:GO:0046872
**Xvis03_207337-PA**	processed-gene-10.83-mRNA-1 protein Name:"Similar to rps5 30S ribosomal protein S5, chloroplastic (Arabidopsis thaliana)" AED:0.00 eAED:0.00 QI:186|1|1|1|1|1|2|216|309	3.17184	-1.4629	0.00756	IPR014721	F:GO:0003735; C:GO:0005840; P:GO:0006412

**Xvis03_223438-PA**	augustus-gene-1.2-mRNA-1 protein Name:"Similar to APX4 Probable L-ascorbate peroxidase 4 (Oryza sativa subsp. japonica)" AED:0.10 eAED:0.10 QI:188|1|1|1|1|9|226|284	3.419 37	- 1.425 4	0.0082 8	IPR002207	F:GO:0004418; P:GO:0033014
**Xvis03_225066-PA**	processed-gene-0.82-mRNA-1 protein Name:"Similar to Chlorophyll a-b binding protein, chloroplastic (Spinacia oleracea)" AED:0.00 eAED:0.00 QI:0|-1|0|1|-1|1|1|0|268	2.783 97	- 1.416 4	0.0093 2	IPR0227 96 (PFAM);	F:GO:0003723; F:GO:0003735; C:GO:0005840; P:GO:0006412; C:GO:0015935
**Xvis03_207668-PA**	processed-gene-12.85-mRNA-1 protein Name:"Similar to PETH Ferredoxin--NADP reductase, leaf isozyme, chloroplastic (Pisum sativum)" AED:0.14 eAED:0.14 QI:231|1|1|1|1|1|9|392|366	9.063 96	- 0.940 5	0.0094 2	IPR001709	F:GO:0004601; P:GO:0006979; F:GO:0020037; P:GO:0055114
**Xvis03_211745-PA**	augustus-gene-13.21-mRNA-1 protein Name:"Similar to TPIP1 Triosephosphate isomerase, cytosolic (Petunia hybrida)" AED:0.34 eAED:0.34 QI:46|0.88|0.8|0.9|1|1|10|0|326	5.568 14	- 1.139 2	0.0097 7	IPR000652	P:GO:0009765; C:GO:0016020
**Xvis03_219001-PA**	processed-gene-2.79-mRNA-1 protein Name:"Similar to Transketolase, chloroplastic (Solanum tuberosum)" AED:0.04 eAED:0.04 QI:238|1|1|1|1|1|7|473|742	12.56 51	-0.754	0.0110 9	IPR0054 75 (PFAM)	F:GO:0016491; P:GO:0055114
**Xvis03_213345-PA**	augustus-gene-10.80-mRNA-1 protein Name:"Similar to PGMP Phosphoglucomutase, chloroplastic (Solanum tuberosum)" AED:0.14 eAED:0.14 QI:0|0.88|0.94|1|0.88|0.89|19|523|691	3.167 3	- 1.382 4	0.0121 9	IPR005841	F:GO:0003824; F:GO:0004807; P:GO:0006096
**Xvis03_217041-PA**	augustus-gene-12.20-mRNA-1 protein Name:"Similar to CPN21 20 kDa chaperonin, chloroplastic (Arabidopsis thaliana)" AED:0.12 eAED:0.12 QI:32|0.33|0.71|0.71|1|1|7|174|298	5.079 52	- 1.101 8	0.0123	IPR020818	F:GO:0003824; F:GO:0004802
**Xvis03_218722-PA**	augustus-gene-3.37-mRNA-1 protein Name:"Similar to ATP synthase subunit delta', mitochondrial (Ipomoea batatas)" AED:0.20 eAED:0.20 QI:67|0|0.25|0.5|0.33|0.75|4|0|208	0.935 03	- 1.859 7	0.0126 6	IPR036771	P:GO:0005975; F:GO:0016868; P:GO:0071704

**Xvis03_200894-PA**	augustus-gene-100.71-mRNA-1 protein Name:"Similar to CURT1D Protein CURVATURE THYLAKOID 1D, chloroplastic (Arabidopsis thaliana)" AED:0.11 eAED:0.11 QI:0|0.42|0.5|0.62|1|1|8|520|390	1.44097	-1.7823	0.01285	IPR025564 (PFAM	P:GO:0006457
**Xvis03_220998-PA**	augustus-gene-7.121-mRNA-1 protein Name:"Similar to ADG2 Glucose-1-phosphate adenylyltransferase large subunit 1, chloroplastic (Arabidopsis thaliana)" AED:0.11 eAED:0.11 QI:196|0.92|0.86|1|0.85|0.86|15|136|540	4.0113	-1.1797	0.01293	G3DSA:2.160.1 0.10 (GENE3D);)	P:GO:0015986; C:GO:0045261; F:GO:0046933
**Xvis03_221570-PA**	augustus-gene-2.10-mRNA-1 protein Name:"Similar to LSF1 Phosphoglucan phosphatase LSF1, chloroplastic (Arabidopsis thaliana)" AED:0.06 eAED:0.06 QI:233|0.83|0.71|1|0.5|0.42|7|831|301	4.03939	-1.3096	0.01298	G3DSA:2.30.42.10 (GENE3D);	C:GO:0009579
**Xvis03_206281-PA**	augustus-gene-15.74-mRNA-1 protein Name:"Similar to CYCL Cytochrome c1-1, heme protein, mitochondrial (Solanum tuberosum)" AED:0.28 eAED:0.28 QI:33|0.85|0.87|1|1|1|8|457|361	1.35325	-1.6927	0.01317	IPR002326 (PRINTS);	P:GO:0009058; F:GO:0016779
**Xvis03_211268-PA**	processed-gene-9.25-mRNA-1 protein Name:"Similar to Vacuolar-processing enzyme (Citrus sinensis)" AED:0.07 eAED:0.07 QI:3|1|1|1|1|1|9|417|590	0.8108	-1.8733	0.01373	IPR001096 (PRINTS);	F:GO:0005515
**Xvis03_215883-PA**	processed-gene-4.2-mRNA-1 protein Name:"Similar to RPL8 60S ribosomal protein L8 (Solanum lycopersicum)" AED:0.00 eAED:0.00 QI:71|1|1|1|1|1|2|167|261	2.48107	-1.4888	0.01419	IPR014726	F:GO:0009055; F:GO:0020037
**Xvis03_218222-PA**	augustus-gene-8.105-mRNA-1 protein Name:"Similar to CURT1A Protein CURVATURE THYLAKOID 1A, chloroplastic (Arabidopsis thaliana)" AED:0.16 eAED:0.16 QI:215|1|1|1|0.8|0.66|6|98|167	2.17954	-1.5739	0.01442	IPR025564 (PFAM);	P:GO:0006508; F:GO:0008233

**Xvis03_205605-PA**	processed-gene-17.119-mRNA-1 protein Name:"Similar to At1g09760 U2 small nuclear ribonucleoprotein A' (Arabidopsis thaliana)" AED:0.23 eAED:0.27 QI:0|0.83|0.71|1|1|1|7|448|347	1.14694	-1.752	0.01459	PF14580 (PFAM);	F:GO:0003723; F:GO:0003735; C:GO:0005840; P:GO:0006412; C:GO:0015934
**Xvis03_220187-PA**	processed-gene-5.6-mRNA-1 protein Name:"Similar to HIR1 Hypersensitive-induced response protein 1 (Arabidopsis thaliana)" AED:0.13 eAED:0.13 QI:38|0.6|0.83|0.83|0.8|0.66|6|280|307	0.67439	-1.8741	0.01459	G3DSA:3.30.479.30 (GENE3D);	C:GO:0009579
**Xvis03_220863-PA**	processed-gene-0.82-mRNA-1 protein Name:"Similar to RPL3 60S ribosomal protein L3 (Oryza sativa subsp. japonica)" AED:0.08 eAED:0.08 QI:54|1|1|1|1|1|6|258|389	3.21493	-1.4162	0.01477	G3DSA:3.30.1430.10 ();	F:GO:0005515
**Xvis03_224754-PA**	processed-gene-1.55-mRNA-1 protein Name:"Similar to At3g10130 Heme-binding-like protein At3g10130, chloroplastic (Arabidopsis thaliana)" AED:0.02 eAED:0.02 QI:0|-1|0|1|-1|1|1|0|209	0.9696	-1.8449	0.01483	IPR006917 (PFAM);	no GO terms
**Xvis03_208987-PA**	augustus-gene-4.4-mRNA-1 protein Name:"Similar to BGAL9 Beta-galactosidase 9 (Arabidopsis thaliana)" AED:0.06 eAED:0.06 QI:63|1|1|1|0.94|0.94|19|309|904	4.0316	-1.1721	0.01497	IPR001944 (PRINTS);	F:GO:0003735; C:GO:0005840; P:GO:0006412
**Xvis03_225829-PA**	augustus-gene-0.17-mRNA-1 protein Name:"Similar to LHCA1 Chlorophyll a-b binding protein 6, chloroplastic (Arabidopsis thaliana)" AED:0.45 eAED:0.45 QI:0|0.44|0.4|0.5|1|1|10|334|459	2.89655	-1.3519	0.01499	PF02466 (PFAM);	no GO terms
**Xvis03_207774-PA**	processed-gene-21.50-mRNA-1 protein Name:"Similar to Transketolase, chloroplastic (Solanum tuberosum)" AED:0.17 eAED:0.17 QI:154|0.85|0.75|1|1|1|8|0|716	12.1963	-0.7238	0.01605	IPR005475 (PFAM)	F:GO:0004553; P:GO:0005975; F:GO:0030246
**Xvis03_216387-PA**	augustus-gene-11.65-mRNA-1 protein Name:"Similar to Alpha-galactosidase (Coffea arabica)" AED:0.16 eAED:0.16 QI:81|1|1|1|0.92|0.92|14|139|642	3.48955	-1.1722	0.0161	IPR002241 (PRINTS)	P:GO:0009765; C:GO:0016020

**Xvis03_216480-PA**	augustus-gene-6.109-mRNA-1 protein Name:"Similar to UVR8 Ultraviolet-B receptor UVR8 (Arabidopsis thaliana)" AED:0.10 eAED:0.10 QI:223|0.9|1|1|1|1|11|290|403	1.054 63	-1.779 3	0.0168 1	IPR000408 (PRINTS);	F:GO:0003824
**Xvis03_224225-PA**	augustus-gene-0.55-mRNA-1 protein Name:"Similar to TPIP1 Triosephosphate isomerase, chloroplastic (Spinacia oleracea)" AED:0.32 eAED:0.32 QI:0|0|0|0.55|0.87|1|9|0|311	5.334 33	-1.076 5	0.0168 7	IPR013785)	F:GO:0003824; F:GO:0004553; P:GO:0005975
**Xvis03_212711-PA**	processed-gene-16.130-mRNA-1 protein Name:"Similar to LHCB4.2 Chlorophyll a-b binding protein CP29.2, chloroplastic (Arabidopsis thaliana)" AED:0.05 eAED:0.05 QI:1875|1|1|1|0.5|0.33|3|204|289	5.229 23	-1.002 2	0.0170 4	IPR022796 (PFAM	F:GO:0009881; P:GO:0010224; F:GO:0042803
**Xvis03_223588-PA**	processed-gene-1.56-mRNA-1 protein Name:"Similar to VIT_19s0014g02480 Probable bifunctional methylthioribulose-1-phosphate dehydratase/enolase-phosphatase E1 1 (Vitis vinifera)" AED:0.24 eAED:0.24 QI:0|0.9|0.90|0.90|1|1|11|237|411	2.125 07	-1.450 9	0.0172 6	IPR036409	F:GO:0003824; F:GO:0004807; P:GO:0006096
**Xvis03_224459-PA**	augustus-gene-1.13-mRNA-1 protein Name:"Similar to RABA2A Ras-related protein RABA2a (Arabidopsis thaliana)" AED:0.02 eAED:0.02 QI:202|1|1|1|1|1|2|509|220	1.266 09	-1.612 3	0.0172 6	PR00449 (PRINTS	P:GO:0009765; C:GO:0016020
**Xvis03_213988-PA**	augustus-gene-11.18-mRNA-1 protein Name:"Similar to PAB1 Proteasome subunit alpha type-2 (Oryza sativa subsp. japonica)" AED:0.05 eAED:0.05 QI:70|1|1|1|1|1|10|615|222	2.815 4	-1.363 4	0.0177 8	IPR029055	F:GO:0000287; C:GO:0005737; F:GO:0016787; P:GO:0019509; F:GO:0043874; F:GO:0046872
**Xvis03_215165-PA**	augustus-gene-14.29-mRNA-1 protein Name:"Similar to EPHX2 Bifunctional epoxide hydrolase 2 (Sus scrofa)" AED:0.25 eAED:0.25 QI:33|1|1|1|0.75|0.6|5|416|321	1.403 31	-1.622 8	0.0185 8	IPR000073 (PRINTS);	F:GO:0003924; F:GO:0005525
**Xvis03_216531-PA**	augustus-gene-9.118-mRNA-1 protein Name:"Similar to RPL3 60S ribosomal protein L3 (Oryza sativa	3.434 94	-1.266	0.0209 3	IPR000597 (PFAM);	F:GO:0004175; F:GO:0004298; C:GO:0005839;

	subsp. japonica)" AED:0.07 eAED:0.07 QI:0|1|0.83|1|1|0.83|6|237|407					P:GO:0006511; C:GO:0019773; P:GO:0051603
**Xvis03_215806-PA**	processed-gene-9.114-mRNA-1 protein Name:"Similar to ANN1 Annexin D1 (Arabidopsis thaliana)" AED:0.04 eAED:0.04 QI:119|1|1|1|1|1|5|186|316	2.396 82	- 1.517 4	0.0211 7	IPR001464 (PRINTS);	F:GO:0003824
**Xvis03_224225-PA**	augustus-gene-0.55-mRNA-1 protein Name:"Similar to TPIP1 Triosephosphate isomerase, chloroplastic (Spinacia oleracea)" AED:0.32 eAED:0.32 QI:0|0|0|0.55|0.87|1|9|0|311	1.629 89	- 1.540 3	0.0213 4	IPR013785	F:GO:0003735; C:GO:0005840; P:GO:0006412
**Xvis03_201588-PA**	augustus-gene-28.7-mRNA-1 protein Name:"Similar to NAD-ME1 NAD-dependent malic enzyme 1, mitochondrial (Arabidopsis thaliana)" AED:0.22 eAED:0.22 QI:0|0.77|0.78|0.86|0.77|0.82|23|290|7 90	1.195 84	-1.668	0.0213 4	IPR001891 (PRINTS);	F:GO:0005509; F:GO:0005544
**Xvis03_219722-PA**	augustus-gene-9.73-mRNA-1 protein Name:"Similar to CAB36 Chlorophyll a-b binding protein 36, chloroplastic (Nicotiana tabacum)" AED:0.36 eAED:0.36 QI:240|1|1|1|1|1|2|393|264	3.822 32	- 1.066 2	0.0223 2	IPR022796 (PFAM	F:GO:0003824; F:GO:0004807; P:GO:0006096
**Xvis03_221307-PA**	processed-gene-5.33-mRNA-1 protein Name:"Similar to Peptide-N4-(N-acetyl-beta-glucosaminyl)asparagine amidase A (Prunus dulcis)" AED:0.04 eAED:0.04 QI:0|-1|0|1|-1|1|1|0|612	1.814 13	- 1.535 9	0.0226	IPR021102 (PFAM)	F:GO:0004470; F:GO:0004471; F:GO:0051287; P:GO:0055114
**Xvis03_205319-PA**	processed-gene-30.54-mRNA-1 protein Name:"Similar to PSAH1 Photosystem I reaction center subunit VI-1, chloroplastic (Arabidopsis thaliana)" AED:0.09 eAED:0.09 QI:15|1|1|1|1|1|3|336|145	1.433 39	- 1.555 6	0.0230 7	IPR004928 (PFAM);	P:GO:0009765; C:GO:0016020
**Xvis03_209971-PA**	augustus-gene-1.88-mRNA-1 protein Name:"Similar to IMPL1 Phosphatase IMPL1, chloroplastic (Arabidopsis thaliana)" AED:0.08 eAED:0.08	0.849 49	- 1.756 2	0.0232 9	IPR020552 (PRINTS);	no GO terms

QI:62|1|1|1|0.9|0.81|11|343|347

**Xvis03_223144-PA**	processed-gene-3.56-mRNA-1 protein Name:"Similar to RBCS1 Ribulose bisphosphate carboxylase small chain, chloroplastic (Musa acuminata)" AED:0.05 eAED:0.05 QI:145|1|1|1|1|1|3|356|174	11.9372	-0.8436	0.025	IPR024681 (PRINTS)	C:GO:0009522; C:GO:0009538; P:GO:0015979
**Xvis03_202356-PA**	processed-gene-29.12-mRNA-1 protein Name:"Similar to PBG1 Proteasome subunit beta type-4 (Arabidopsis thaliana)" AED:0.13 eAED:0.13 QI:0|0.71|0.75|0.87|1|1|8|335|329	2.24439	-1.361	0.02579	IPR029055	F:GO:0008934; P:GO:0046854; P:GO:0046855
**Xvis03_212711-PA**	processed-gene-16.130-mRNA-1 protein Name:"Similar to LHCB4.2 Chlorophyll a-b binding protein CP29.2, chloroplastic (Arabidopsis thaliana)" AED:0.05 eAED:0.05 QI:1875|1|1|1|0.5|0.33|3|204|289	4.29118	-1.0098	0.02717	IPR022796 (PFAM	no GO terms
**Xvis03_213887-PA**	remorin-like isoform X2	3.12009	-1.2184	0.02722	IPR005516 (PFAM	F:GO:0004298; C:GO:0005839; P:GO:0051603
**Xvis03_202061-PA**	processed-gene-6.69-mRNA-1 protein Name:"Similar to RPL9 50S ribosomal protein L9, chloroplastic (Arabidopsis thaliana)" AED:0.06 eAED:0.06 QI:100|1|1|1|0.83|0.71|7|568|196	1.47336	-1.5557	0.02735	IPR020069 (PFAM)	P:GO:0009765; C:GO:0016020
**Xvis03_202428-PA**	augustus-gene-35.90-mRNA-1 protein Name:"Similar to CURT1A Protein CURVATURE THYLAKOID 1A, chloroplastic (Arabidopsis thaliana)" AED:0.22 eAED:0.42 QI:0|0.75|0.6|1|0.75|0.6|5|269|208	2.03114	-1.4203	0.02897	IPR025564 (PFAM)	no GO terms
**Xvis03_203635-PA**	augustus-gene-35.43-mRNA-1 protein Name:"Similar to CLPP5 ATP-dependent Clp protease proteolytic subunit 5, chloroplastic (Arabidopsis thaliana)" AED:0.40 eAED:0.40 QI:0|1|0.71|1|1|1|7|484|324	1.51163	-1.5248	0.02928	IPR001907 (PRINTS)	F:GO:0003735; C:GO:0005840; P:GO:0006412

**Xvis03_208040-PA**	processed-gene-21.10-mRNA-1 protein Name:"Similar to SHM4 Serine hydroxymethyltransferase 4 (Arabidopsis thaliana)" AED:0.05 eAED:0.05 QI:76|1|1|1|1|1|4|235|515	15.57 43	- 0.612 9	0.0293 1	IPR015421	C:GO:0009579
**Xvis03_217729-PA**	processed-gene-11.5-mRNA-1 protein Name:"Similar to PETE Plastocyanin minor isoform, chloroplastic (Arabidopsis thaliana)" AED:0.37 eAED:0.37 QI:0|0|0|0.16|1|1|6|0|366	2.785 06	- 1.268 9	0.0302 2	IPR0023 87 (PRINTS	F:GO:0004252; P:GO:0006508
**Xvis03_225147-PA**	augustus-gene-0.41-mRNA-1 protein Name:"Similar to CAB6A Chlorophyll a-b binding protein 6A, chloroplastic (Solanum lycopersicum)" AED:0.00 eAED:0.00 QI:106|1|1|1|1|1|3|75|204	3.862 29	- 1.046 7	0.0309 5	IPR022796 (PFAM); SSF103511 (SUPERFAMI L Y)	F:GO:0003824; F:GO:0004372; P:GO:0019264; F:GO:0030170; P:GO:0035999
**Xvis03_216230-PA**	Basic secretory protease	1.585 22	-1.482	0.0310 8	IPR007541 (PFAM)	F:GO:0005507; F:GO:0009055
**Xvis03_217514-PA**	processed-gene-7.97-mRNA-1 protein Name:"Similar to SPAC9E9.15 Uncharacterized protein C9E9.15 (Schizosaccharomyces pombe (strain 972 / ATCC 24843))" AED:0.14 eAED:0.14 QI:59|0.93|0.87|1|1|1|16|145|594	3.507 08	- 1.103 5	0.0317 2	G3DSA:2.60. 12 0.430	P:GO:0009765; C:GO:0016020
**Xvis03_207968-PA**	augustus-gene-15.42-mRNA-1 protein Name:"Similar to CAP10A Chlorophyll a-b binding protein CP24 10A, chloroplastic (Solanum lycopersicum)" AED:0.00 eAED:0.00 QI:218|1|1|1|1|1|2|259|254	0.648 29	-1.688	0.0319 8	IPR023329	no GO terms
**Xvis03_212120-PA**	augustus-gene-8.8-mRNA-1 protein Name:"Similar to CEST Protein CHLOROPLAST ENHANCING STRESS TOLERANCE, chloroplastic (Oryza sativa subsp. japonica)" AED:0.04 eAED:0.04 QI:85|1|1|1|1|1|6|455|259	1.043 13	- 1.619 3	0.0320 7	PTHR33672: SF 3 (PANTHER);	P:GO:0032981
**Xvis03_223438-PA**	augustus-gene-1.2-mRNA-1 protein Name:"Similar to APX4 Probable L-ascorbate peroxidase 4 (Oryza sativa subsp. japonica)" AED:0.10 eAED:0.10 QI:188|1|1|1|1|1|9|226|284	2.609 42	- 1.204 9	0.0321	IPR0022 07 (PRINTS);	P:GO:0009765; C:GO:0016020

**Xvis03_211391-PA**	processed-gene-20.57-mRNA-1 protein Name:"Similar to GGH2 Gamma-glutamyl hydrolase 2 (Arabidopsis thaliana)" AED:0.20 eAED:0.20 QI:39|1|0.85|1|1|1|7|0|302	2.254 12	-1.310 1	0.0322 2	IPR0116 97 (PFAM	C:GO:0009535; P:GO:0048564; P:GO:0080183
**Xvis03_224849-PA**	processed-gene-1.76-mRNA-1 protein Name:"Similar to FKBP19 Peptidyl-prolyl cis-trans isomerase FKBP19, chloroplastic (Arabidopsis thaliana)" AED:0.46 eAED:0.49 QI:0|0.72|0.83|0.91|0.90|0.83|12|203|3 20	0.704 45	-1.651 5	0.0329 7	IPR0011 79 (PFAM)	F:GO:0004601; P:GO:0006979; F:GO:0020037; P:GO:0055114
**Xvis03_220733-PA**	processed-gene-1.13-mRNA-1 protein Name:"Similar to CAT1 Catalase-1 (Triticum aestivum)" AED:0.19 eAED:0.19 QI:0|0|0|0.66|1|1|9|0|623	6.462 83	-0.862 1	0.0330 2	IPR0180 28 (PRINTS)	F:GO:0008242; F:GO:0016787
**Xvis03_223095-PA**	augustus-gene-3.96-mRNA-1 protein Name:"Similar to petC Cytochrome b6-f complex iron-sulfur subunit, chloroplastic (Oryza sativa subsp. japonica)" AED:0.11 eAED:0.11 QI:167|0.33|0.75|1|1|1|4|287|178	1.792 06	-1.354 2	0.0338 3	IPR0058 05 (PRINTS)	no GO terms
**Xvis03_216531-PA**	augustus-gene-9.118-mRNA-1 protein Name:"Similar to RPL3 60S ribosomal protein L3 (Oryza sativa subsp. japonica)" AED:0.07 eAED:0.07 QI:0|1|0.83|1|1|0.83|6|237|407	3.111 12	-1.257 7	0.034	IPR000597 (PFAM)	F:GO:0004096; P:GO:0006979; F:GO:0020037; P:GO:0055114
**Xvis03_220973-PA**	augustus-gene-5.51-mRNA-1 protein Name:"Similar to RPS2C 40S ribosomal protein S2-3 (Arabidopsis thaliana)" AED:0.29 eAED:0.29 QI:54|1|1|1|0.5|0.33|3|154|274	3.119 06	-1.164 4	0.0349 5	IPR013810 (PFAM);	F:GO:0008121; F:GO:0009496; C:GO:0009507; C:GO:0016020; F:GO:0016491; F:GO:0016679; C:GO:0042651; F:GO:0045158; F:GO:0051537; P:GO:0055114
**Xvis03_213605-PA**	processed-gene-14.84-mRNA-1 protein Name:"Similar to Os08g0288200 Probable adenylate kinase 5, chloroplastic (Oryza sativa subsp. japonica)" AED:0.22 eAED:0.22	0.701 86	-1.619 5	0.0353 4	IPR000850 (PRINTS);	F:GO:0003735; C:GO:0005840; P:GO:0006412

QI:25|1|0.94|1|1|1|17|0|611

**Xvis03_202672-PA**	processed-gene-54.103-mRNA-1 protein Name:"Similar to HEMC Porphobilinogen deaminase, chloroplastic (Oryza sativa subsp. japonica)" AED:0.01 eAED:0.01 QI:117|1|1|1|1|1|5|327|378	4.01403	-1.0024	0.03547	IPR000860 (PRINTS)	F:GO:0003723; F:GO:0003735; C:GO:0005840; P:GO:0006412; C:GO:0015935
**Xvis03_207337-PA**	processed-gene-10.83-mRNA-1 protein Name:"Similar to rps5 30S ribosomal protein S5, chloroplastic (Arabidopsis thaliana)" AED:0.00 eAED:0.00 QI:186|1|1|1|1|1|2|216|309	0.96041	-1.5832	0.03649	IPR014721	F:GO:0004017; F:GO:0005524; P:GO:0006139; F:GO:0016776; F:GO:0019205
**Xvis03_207968-PA**	augustus-gene-15.42-mRNA-1 protein Name:"Similar to CAP10A Chlorophyll a-b binding protein CP24 10A, chloroplastic (Solanum lycopersicum)" AED:0.00 eAED:0.00 QI:218|1|1|1|1|1|2|259|254	0.92133	-1.5542	0.0365	IPR023329	F:GO:0004418; P:GO:0033014
**Xvis03_212925-PA**	processed-gene-5.93-mRNA-1 protein Name:"Similar to PETH Ferredoxin--NADP reductase, leaf-type isozyme, chloroplastic (Nicotiana tabacum)" AED:0.42 eAED:0.49 QI:0|0|0|0.8|1|1|5|0|177	1.71463	-1.4288	0.03651	IPR001433 (PFAM)	F:GO:0003723; F:GO:0003735; C:GO:0005840; P:GO:0006412; C:GO:0015935
**Xvis03_211745-PA**	augustus-gene-13.21-mRNA-1 protein Name:"Similar to TPIP1 Triosephosphate isomerase, cytosolic (Petunia hybrida)" AED:0.34 eAED:0.34 QI:46|0.88|0.8|0.9|1|1|10|0|326	4.98432	-0.9345	0.03734	IPR000652	P:GO:0009765; C:GO:0016020
**Xvis03_212711-PA**	processed-gene-16.130-mRNA-1 protein Name:"Similar to LHCB4.2 Chlorophyll a-b binding protein CP29.2, chloroplastic (Arabidopsis thaliana)" AED:0.05 eAED:0.05 QI:1875|1|1|1|0.5|0.33|3|204|289	3.26057	-1.0666	0.03818	IPR022796 (PFAM)	F:GO:0016491; P:GO:0055114
**Xvis03_223588-PA**	processed-gene-1.56-mRNA-1 protein Name:"Similar to VIT_19s0014g02480 Probable bifunctional methylthioribulose-1-phosphate dehydratase/enolase-phosphatase E1 1 (Vitis vinifera)" AED:0.24 eAED:0.24 QI:0|0.9|0.90|0.90|1|1|11|237|411	9.12494	-0.9422	0.03981	IPR036409	F:GO:0003824; F:GO:0004807; P:GO:0006096

**Xvis03_224459-PA**	augustus-gene-1.13-mRNA-1 protein Name:"Similar to RABA2A Ras-related protein RABA2a (Arabidopsis thaliana)" AED:0.02 eAED:0.02 QI:202|1|1|1|1|1|2|509|220	2.84135	-1.1073	0.0404	PR00449 (PRINTS)	P:GO:0009765; C:GO:0016020
**Xvis03_213988-PA**	augustus-gene-11.18-mRNA-1 protein Name:"Similar to PAB1 Proteasome subunit alpha type-2 (Oryza sativa subsp. japonica)" AED:0.05 eAED:0.05 QI:70|1|1|1|1|1|10|615|222	1.45642	-1.4357	0.04396	IPR029055	F:GO:0000287; C:GO:0005737; F:GO:0016787; P:GO:0019509; F:GO:0043874; F:GO:0046872
**Xvis03_207968-PA**	augustus-gene-15.42-mRNA-1 protein Name:"Similar to CAP10A Chlorophyll a-b binding protein CP24 10A, chloroplastic (Solanum lycopersicum)" AED:0.00 eAED:0.00 QI:218|1|1|1|1|1|2|259|254	1.21025	-1.4563	0.04448	IPR023329	F:GO:0003924; F:GO:0005525
**Xvis03_224672-PA**	processed-gene-1.89-mRNA-1 protein Name:"Similar to ALATS Alanine--tRNA ligase (Arabidopsis thaliana)" AED:0.10 eAED:0.10 QI:33|1|1|1|0.90|0.90|22|389|993	5.12135	-0.9111	0.04584	IPR002318 (PRINTS)	F:GO:0004175; F:GO:0004298; C:GO:0005839; P:GO:0006511; C:GO:0019773; P:GO:0051603
**Xvis03_208742-PA**	augustus-gene-3.45-mRNA-1 protein Name:"Similar to At4g13360 3- hydroxyisobutyryl-CoA hydrolase- like protein 3, mitochondrial (Arabidopsis thaliana)" AED:0.08 eAED:0.08 QI:31|1|1|1|0.88|0.9|10|444|426	2.07578	-1.188	0.04736	IPR032259 (PFAM)	P:GO:0009765; C:GO:0016020
**Xvis03_219507-PA**	augustus-gene-9.55-mRNA-1 protein Name:"Similar to POPTRDRAFT_831870 Biotin carboxylase 1, chloroplastic (Populus trichocarpa)" AED:0.11 eAED:0.11 QI:0|1|0.94|1|0.93|0.76|17|386|550	1.25457	-1.3382	0.04879	IPR005481 (PFAM)	F:GO:0000166; F:GO:0003676; F:GO:0004812; F:GO:0004813; F:GO:0005524; C:GO:0005737; P:GO:0006419; P:GO:0043039
**Xvis03_224372-PA**	processed-gene-2.16-mRNA-1 protein Name:"Similar to UAM1 UDP-arabinopyranose mutase 1 (Oryza sativa subsp. japonica)" AED:0.08 eAED:0.08 QI:87|1|1|1|1|1|4|347|359	1.29159	-1.3747	0.0489	IPR037595 (PFAM)	F:GO:0003860

**Xvis03_200047-PA**	augustus-gene-4.43-mRNA-1 protein Name:"Similar to BGLU22 Beta-glucosidase 22 (Oryza sativa subsp. japonica)" AED:0.38 eAED:0.38 QI:56|0.9|0.81|1|1|1|11|40|465	0.951 13	-1.505	0.0496 3	IPR001360	F:GO:0005524; F:GO:0016874; F:GO:0046872
**Xvis03_212711-PA**	processed-gene-2.106-mRNA-1 protein Name:"Similar to Beta-galactosidase (Asparagus officinalis)" AED:0.13 eAED:0.13 QI:277|1|1|1|1|1|19|418|832	1.951 18	- 1.341 2	0.0498 5	IPR001944	F:GO:0016866; P:GO:0071669
		5.229 23	- 3.599 1	1.84E-09	P:GO:000976 5; C:GO:001602 0	F:GO:0004553; P:GO:0005975
**Xvis03_216014-PA**	processed-gene-5.37-mRNA-1 protein Name:"Similar to PSAF Photosystem I reaction center subunit III, chloroplastic (Arabidopsis thaliana)" AED:0.24 eAED:0.24 QI:0|-1|0|1|-1|1|1|0|232	4.662 08	- 1.326 6	0.0053 4		F:GO:0004553; P:GO:0005975; F:GO:0030246
**Xvis03_220507-PA**	Xvis03_220507-PA processed-gene-1.26-mRNA-1 protein Name:"Similar to PAP27 Probable inactive purple acid phosphatase 27 (Arabidopsis thaliana)" AED:0.08 eAED:0.08 QI:27|1|1|1|1|1|12|227|617	0.960 41	- 2.079 6	0.0061 4		P:photosynthesis , light harvesting; C:membrane
**Xvis03_219507-PA**	augustus-gene-9.55-mRNA-1 protein Name:"Similar to POPTRDRAFT_831870 Biotin carboxylase 1, chloroplastic (Populus trichocarpa)" AED:0.11 eAED:0.11 QI:0|1|0.94|1|0.93|0.76|17|386|550	1.950 77	- 1.779 7	0.0062 2	F:ATP binding; F:ligase activity; F:metal ion binding	
**Xvis03_208742-PA**	augustus-gene-3.45-mRNA-1 protein Name:"Similar to At4g13360 3-hydroxyisobutyryl-CoA hydrolase-like protein 3, mitochondrial (Arabidopsis thaliana)" AED:0.08 eAED:0.08 QI:31|1|1|1|0.88|0.9|10|444|426	1.922 39	- 1.492 5	0.0112 6	F:3-hydroxyisobutyryl-CoA hydrolase activity	
**Xvis03_224672-PA**	processed-gene-1.89-mRNA-1 protein Name:"Similar to ALATS Alanine--tRNA ligase (Arabidopsis thaliana)" AED:0.10 eAED:0.10 QI:33|1|1|1|0.90|0.90|22|389|993	2.560 21	- 1.494 7	0.0116 1	F:nucleic acidbinding; F:alanine tRNA ligase activity; F:ATP binding;	

					C:cytoplasm; P:alanyl tRNAaminoacyl ation	
**Xvis03_224372-PA**	processed-gene-2.16-mRNA-1 protein Name:"Similar to UAM1 UDP-arabinopyranose mutase 1 (Oryza sativa subsp. japonica)" AED:0.08 eAED:0.08 QI:87|1|1|1|1|1|4|347|359	1.041 09	- 1.795 1	0.0117 3	F:intramolecula r transferase activity; P:plant-type cell wall organization or biogenesis	
**Xvis03_200047-PA**	augustus-gene-4.43-mRNA-1 protein Name:"Similar to BGLU22 Beta-glucosidase 22 (Oryza sativa subsp. japonica)" AED:0.38 eAED:0.38 QI:56|0.9|0.81|1|1|1|11|40|465	2.010 6	- 1.477 1	0.0119 1	F:hydrolase activity, hydrolyzing O-glycosyl compounds; P:carbohydrate metabolic process	
**Xvis03_223612-PA**	augustus-gene-2.49-mRNA-1 protein Name:"Similar to CEBIP Chitin elicitor-binding protein (Oryza sativa subsp. japonica)" AED:0.27 eAED:0.27 QI:0|0.6|0.5|0.66|1|1|6|353|477	3.499 38	- 1.276 4	0.0125 9	IPR036779	
**Xvis03_213033-PA**	processed-gene-15.37-mRNA-1 protein Name:"Similar to zta1 Probable quinone oxidoreductase (Schizosaccharomyces pombe (strain 972 / ATCC 24843))" AED:0.20 eAED:0.20 QI:0|0|0|0.8|1|1|5|0|292	0.901 09	-1.882	0.0145 5	IPR013154 (PFAM);	
**Xvis03_225238-PA**	augustus-gene-0.18-mRNA-1 protein Name:"Similar to WAXY Granule-bound starch synthase 1, chloroplastic/amyloplastic (Antirrhinum majus)" AED:0.11 eAED:0.11 QI:73|1|1|1|0.92|0.85|14|142|611	0.877 06	- 1.844 4	0.0163 3	IPR013534 (PFAM)	no GO terms
**Xvis03_210933-PA**	augustus-gene-1.32-mRNA-1 protein Name:"Similar to PAP2 Probable inactive purple acid phosphatase 2 (Arabidopsis thaliana)" AED:0.04 eAED:0.04 QI:0|1|0.66|1|0.5|0.33|3|1759|658	3.104 01	- 1.200 6	0.0169 6	IPR015914 (PFAM)	P:GO:0055114

**Xvis03_218675-PA**	processed-gene-10.89-mRNA-1 protein Name:"Similar to AGD2 Probable LL-diaminopimelate aminotransferase, chloroplastic (Oryza sativa subsp. japonica)" AED:0.20 eAED:0.20 QI:0|0.85|0.75|1|1|1|8|209|407	0.98322	-1.7713	0.01755	IPR004839 (PFAM)	F:GO:0004373
**Xvis03_212280-PA**	processed-gene-6.79-mRNA-1 protein Name:"Similar to THI1-2 Thiamine thiazole synthase 2, chloroplastic (Vitis vinifera)" AED:0.02 eAED:0.02 QI:212|1|1|1|1|1|2|483|354	3.04448	-1.2108	0.0182	IPR002922	F:GO:0003993; F:GO:0016787; F:GO:0046872
**Xvis03_205558-PA**	augustus-gene-14.7-mRNA-1 protein Name:"Similar to CAB7 Chlorophyll a-b binding protein 7, chloroplastic (Solanum lycopersicum)" AED:0.16 eAED:0.16 QI:0|0.25|0.4|1|0.75|0.8|5|892|278	0.76517	-1.7151	0.02405	IPR022796	F:GO:0003824; F:GO:0008483; P:GO:0009058; F:GO:0030170
**Xvis03_205102-PA**	processed-gene-9.15-mRNA-1 protein Name:"Similar to GOR Glutathione reductase, chloroplastic (Fragment) (Nicotiana tabacum)" AED:0.14 eAED:0.14 QI:0|1|0.90|1|0.9|0.81|11|639|590	2.8642	-1.2104	0.02478	PR00411	P:GO:0009228
**Xvis03_222506-PA**	augustus-gene-4.97-mRNA-1 protein Name:"Similar to EDA2 Probable serine protease EDA2 (Arabidopsis thaliana)" AED:0.31 eAED:0.31 QI:159|0.92|0.92|1|1|1|14|279|488	1.7679	-1.4029	0.02497	IPR029058	P:GO:0009765; C:GO:0016020
**Xvis03_225868-PA**	augustus-gene-0.5-mRNA-1 protein Name:"Similar to caa43 2-haloacrylate reductase (Burkholderia sp.)" AED:0.18 eAED:0.18 QI:0|0.25|0|0.8|0.5|0.4|5|0|313	0.8474	-1.667	0.02744	IPR013149 (PFAM)	F:GO:0004362; P:GO:0006749; F:GO:0016491; P:GO:0045454; F:GO:0050660; F:GO:0050661; P:GO:0055114
**Xvis03_204322-PA**	processed-gene-9.15-mRNA-1 protein Name:"Similar to AFRR Monodehydroascorbate reductase (Solanum lycopersicum)" AED:0.13 eAED:0.13 QI:138|0.72|0.83|0.83|1|1|12|368|489	4.19782	-1.0042	0.02888	PR00368 (	P:GO:0006508; F:GO:0008236

**Xvis03_211689-PA**	processed-gene-6.93-mRNA-1 protein Name:"Similar to Proteasome subunit beta type-5 (Spinacia oleracea)" AED:0.07 eAED:0.07 QI:0|1|0.87|1|1|1|8|489|310	2.204 52	- 1.270 7	0.0289 9	IPR000243	P:GO:0055114
**Xvis03_217074-PA**	processed-gene-2.49-mRNA-1 protein Name:"Similar to PME35 Probable pectinesterase/pectinesterase inhibitor 35 (Arabidopsis thaliana)" AED:0.36 eAED:0.36 QI:662|1|1|1|1|1|3|218|442	2.798 6	- 1.409 7	0.0352 3	IPR035513	F:GO:0016491; F:GO:0050660; P:GO:0055114
**Xvis03_201017-PA**	processed-gene-109.26-mRNA-1 protein Name:"Similar to PPD Pyruvate, phosphate dikinase, chloroplastic (Mesembryanthemum crystallinum)" AED:0.09 eAED:0.09 QI:8|1|1|1|0.89|0.9|20|257|985	0.631 76	- 1.637 5	0.0374 8		F:GO:0004175; F:GO:0004298; C:GO:0005839; P:GO:0051603
**Xvis03_221989-PA**	augustus-gene-2.19-mRNA-1 protein Name:"Similar to dapb3 Dipeptidyl aminopeptidase BIII (Pseudoxanthomonas mexicana)"	0.750 18	- 1.628 1	0.0375 8	IPR011042	F:GO:0004857; F:GO:0030599; P:GO:0042545
**Xvis03_200779-PA**	processed-gene-89.11-mRNA-1 protein Name:"Similar to PSAL Photosystem I reaction center subunit XI, chloroplastic (Spinacia oleracea)" AED:0.06 eAED:0.06 QI:217|1|1|1|1|1|2|190|220	0.852 47	- 1.576 8			
**Xvis03_217230-PA**	processed-gene-4.43-mRNA-1 protein Name:"Similar to PREP1 Presequence protease 1, chloroplastic/mitochondrial (Arabidopsis thaliana)" AED:0.19 eAED:0.19 QI:1251|0.91|0.84|1|0.75|0.69|13|382|303	1.512 92	- 1.447 6	0.0389 4	IPR036592	F:GO:0003824; F:GO:0005524; P:GO:0006090; F:GO:0016301; P:GO:0016310; F:GO:0016772; F:GO:0050242
**Xvis03_205724-PA**	processed-gene-33.32-mRNA-1 protein Name:"Similar to IBI1 Aspartate--tRNA ligase 2, cytoplasmic (Arabidopsis thaliana)" AED:0.10 eAED:0.10 QI:60|0.77|0.8|1|1|1|10|190|552	0.943 27	- 1.558 1	0.0389 5		P:GO:0006508; F:GO:0008236
**Xvis03_220977-PA**	processed-gene-6.91-mRNA-1 protein Name:"Similar to CYP74B2 Linolenate hydroperoxide lyase, chloroplastic (Arabidopsis thaliana)" AED:0.02 eAED:0.17 QI:0|-1|0|1|-1|1|1|0|486	2.324 82	- 1.253 7	0.0408 6	IPR002312 (PRINTS)	C:GO:0009522; C:GO:0009538; P:GO:0015979

**Xvis03_210782-PA**	augustus-gene-7.64-mRNA-1 protein Name:"Similar to Os08g0562700 Puromycin-sensitive aminopeptidase (Oryza sativa subsp. japonica)" AED:0.20 eAED:0.20 QI:160|0.75|0.81|0.90|0.84|0.84|33|284|1052	5.676 78	- 0.899 6	0.0411 1	IPR002403 (PRINTS);	F:GO:0003824; P:GO:0006508; F:GO:0046872
**Xvis03_219931-PA**	processed-gene-8.160-mRNA-1 protein Name:"Similar to SAG12 Senescence-specific cysteine protease SAG12 (Arabidopsis thaliana)" AED:0.00 eAED:0.00 QI:0|1|0.5|1|1|1|2|268|331	1.173 47	- 1.471 4	0.0416 5	IPR024601 (PFAM)	F:GO:0000166; F:GO:0003676; F:GO:0004812; F:GO:0004815; F:GO:0005524; C:GO:0005737; P:GO:0006418; P:GO:0006422
**Xvis03_219642-PA**	augustus-gene-3.70-mRNA-1 protein Name:"Similar to Serpin-Z1C (Triticum aestivum)" AED:0.04 eAED:0.04 QI:7|1|1|1|0.5|0.33|3|183|391	2.219 59	- 1.134 1	0.0433 5	IPR00066	F:GO:0004497; F:GO:0005506; F:GO:0016705; F:GO:0020037; P:GO:0055114
**Xvis03_221434-PA**	processed-gene-2.106-mRNA-1 protein Name:"Similar to Beta- galactosidase (Asparagus officinalis)" AED:0.13 eAED:0.13 QI:277|1|1|1|1|1|19|418|832	2.435 57	- 1.244 5	0.0459 9	IPR023796 (PFAM)	F:GO:0008237; F:GO:0008270
**Xvis03_215806-PA**	processed-gene-9.114-mRNA-1 protein Name:"Similar to ANN1 Annexin D1 (Arabidopsis thaliana)" AED:0.04 eAED:0.04 QI:119|1|1|1|1|1|5|186|316	2.396 82	- 1.230 1	0.0461 7	F:hydrolase activity, hydrolyzing O- glycosyl compounds; P:carbohydrate metabolic process; F:carbohydrate binding	P:GO:0006508; F:GO:0008234
				0.0489 7	F:calcium ion binding; F:calcium- dependent phospholipid binding	

Table S4: Upregulated proteins identified in *X. schlechteri* leaf tissue in response to dehydration at different dehydration stages. Early Response to Dehydration (ERD), Mid Response to Dehydration (MRD) and Late Response to Dehydration (LRD). The values represent the level of protein expression observed in each dehydration stage obtained from label-free quantitative proteomics data.

Protein Name	ERD	MRD	LRD	Dehydration Stage
**11 kDa late embryogenesis abundant protein (Helianthus annuus)**	.	1.655059944	2.557663979	MRD and LRD
**1-Cys peroxiredoxin (Medicago truncatula)**	.	.	1.335126495	LRD
**29 kDa ribonucleoprotein B, chloroplastic (Nicotiana sylvestris)**	.	.	0.951861733	LRD
**AAE3 Oxalate--CoA ligase (Arabidopsis thaliana)**	1.706599422	2.36880206	2.657314544	Across all dehydration stages
**ABP20 Auxin-binding protein ABP20 (Prunus persica)**	.	0.726395826	.	MRD
**AGT1 Serine--glyoxylate aminotransferase (Arabidopsis thaliana)**	0.795651548	.	.	ERD
**AKR2 Ankyrin repeat domain-containing protein 2 (Arabidopsis thaliana)**		.	1.77529497	LRD
**Aldose reductase (Hordeum vulgare)**	.	1.943676029	2.13724642	MRD and LRD
**AN3 Naringenin,2-oxoglutarate 3-dioxygenase (Fragment) (Petunia hybrida)**	.	.	1.664133089	LRD
**At3g10130 Heme-binding-like protein At3g10130, chloroplastic (Arabidopsis thaliana)**	.	.	1.621225267	LRD
**At4g12130 Putative transferase At4g12130, mitochondrial (Arabidopsis thaliana)**	.	.	1.545694996	LRD
**At4g13010 Putative quinone-oxidoreductase homolog, chloroplastic (Arabidopsis thaliana)**	.	.	2.458639066	LRD
**At4g22670 FAM10 family protein At4g22670 (Arabidopsis thaliana)**	.	.	2.096229206	LRD
**At5g12110 Elongation factor 1-beta 1 (Arabidopsis thaliana)**	.	.	1.157181359	LRD
**At5g13200 GEM-like protein 5 (Arabidopsis thaliana)**	.	.	2.050192399	LRD
**At5g39570 Uncharacterized protein At5g39570 (Arabidopsis thaliana)**	.	.	1.655206338	LRD
**At5g61250 Heparanase-like protein 2 (Arabidopsis thaliana)**	0.954132089	.	.	ERD
**atad1a ATPase family AAA domain-containing protein 1-A (Danio rerio)**	.	.	1.570578594	LRD
**B19.4 Late embryogenesis abundant protein B19.4 (Hordeum vulgare)**	1.159483161	1.816389763	2.516909095	Across all dehydration stages

**Blue copper protein (Pisum sativum)**	.	1.604443074	.	MRD
**Bowman-Birk type trypsin inhibitor (Triticum aestivum)**	1.490311914	.	2.18560715	ERD and LRD
**CAISE5 Glucose and ribitol dehydrogenase (Daucus carota)**	.	.	1.170057472	LRD
**CAL1 Calmodulin (Medicago sativa)**	1.635472647	.	.	ERD
**Carbonic anhydrase, chloroplastic (Hordeum vulgare)**	.	.	1.063629616	LRD
**CCR1 Cinnamoyl-CoA reductase 1 (Arabidopsis thaliana)**	1.561330267	1.623791745	2.600421553	Across all dehydration stages
**CCT2 Choline-phosphate cytidylyltransferase 2 (Arabidopsis thaliana)**	.	1.891082235	2.434010574	MRD and LRD
**CHLD Magnesium-chelatase subunit ChlD, chloroplastic (Oryza sativa subsp. japonica)**	.	.	2.199000455	LRD
**CHLI Magnesium-chelatase subunit ChlI, chloroplastic (Oryza sativa subsp. japonica)**	.	.	2.663002746	LRD
**CLPB3 Chaperone protein ClpB3, chloroplastic (Arabidopsis thaliana)**	.	.	1.718787179	LRD
**CML18 Probable calcium-binding protein CML18 (Oryza sativa subsp. japonica)**	.	1.972359689	2.643203935	MRD and LRD
**COR47 Dehydrin COR47 (Arabidopsis thaliana)**	.	.	1.720897276	LRD
**CSD2 Superoxide dismutase [Cu-Zn] 2, chloroplastic (Arabidopsis thaliana)**	.	.	0.770670729	LRD
**CSP1 Cold shock protein 1 (Arabidopsis thaliana)**	.	1.625479205	2.163496526	MRD and LRD
**CSP4 Cold shock domain-containing protein 4 (Arabidopsis thaliana)**	.	.	1.282197447	LRD
**CYP74A2 Allene oxide synthase 2 (Oryza sativa subsp. japonica)**	0.859536898	.	.	ERD
**CYS6 Cysteine proteinase inhibitor 6 (Arabidopsis thaliana)**	.	.	1.091432261	LRD
**Desiccation-related protein PCC13-62 (Craterostigma plantagineum)**	.	1.325612786	1.66968991	MRD and LRD
**Desiccation-related protein PCC3-06 (Craterostigma plantagineum)**	1.709026897	2.741681551	3.802948804	Across all dehydration stages
**DHN1 Dehydrin DHN1 (Hordeum vulgare)**	.	.	2.239287919	LRD
**DHN3 Dehydrin DHN3 (Hordeum vulgare)**	.	1.134434404	1.889777148	MRD and LRD
**ECU03_1610 Uncharacterized protein ECU03_1610 (Encephalitozoon cuniculi (strain GB-M1))**	.	.	3.039136047	LRD
**EIF-5A2 Eukaryotic translation initiation factor 5A-2 (Nicotiana plumbaginifolia)**	.	.	2.095729012	LRD

**Embryonic protein DC-8 (Daucus carota)**	.	1.456424806	2.545812228	MRD and LRD
**ERF1-3 Eukaryotic peptide chain release factor subunit 1-3 (Arabidopsis thaliana)**	.	1.543122406	2.677097134	MRD and LRD
**Eukaryotic translation initiation factor 5A (Manihot esculenta)**	.	.	1.934951423	LRD
**FEN1 Flap endonuclease 1 (Zea mays)**	.	.	2.177422956	LRD
**FLK Flowering locus K homology domain (Arabidopsis thaliana)**	.	.	1.960057559	LRD
**Fructose-bisphosphate aldolase, cytoplasmic isozyme 1 (Pisum sativum)**	.	.	0.979214196	LRD
**GAPC3 Glyceraldehyde-3-phosphate dehydrogenase 3, cytosolic (Oryza sativa subsp. japonica)**	.	0.869467807	.	MRD
**GDCSH Glycine cleavage system H protein, mitochondrial (Flaveria anomala)**	.	.	1.545745923	LRD
**GDCSP Glycine dehydrogenase (decarboxylating), mitochondrial (Solanum tuberosum)**	0.756563035	.	.	ERD
**GDCSPA Glycine dehydrogenase (decarboxylating) A, mitochondrial (Flaveria pringlei)**	0.870276947	.	.	ERD
**GLU Ferredoxin-dependent glutamate synthase, chloroplastic (Oryza sativa subsp. japonica)**	1.093872984	.	.	ERD
**Glutaredoxin (Ricinus communis)**	.	.	1.167181121	LRD
**GLX2-2 Hydroxyacylglutathione hydrolase cytoplasmic (Arabidopsis thaliana)**	.	.	1.66394102	LRD
**GMPM1 18 kDa seed maturation protein (Glycine max)**	.	.	2.588554158	LRD
**GSA Glutamate-1-semialdehyde 2,1-aminomutase, chloroplastic (Oryza sativa subsp. japonica)**	.	.	0.936886678	LRD
**Histone H4 (Glycine max)**	.	0.816958339	.	MRD
**HOP3 Hsp70-Hsp90 organizing protein 3 (Arabidopsis thaliana)**	.	.	2.004820501	LRD
**HSP70 Heat shock cognate 70 kDa protein (Petunia hybrida)**	.	0.607533066	.	MRD
**HSP70 Stromal 70 kDa heat shock-related protein, chloroplastic (Pisum sativum)**	.	.	0.554872272	LRD
**HSP81-1 Heat shock protein 81-1 (Oryza sativa subsp. japonica)**	.	.	1.584931903	LRD
**Hsp83 Heat shock protein 83 (Drosophila melanogaster)**	.	.	1.098741349	LRD
**HVA22A HVA22-like protein a (Arabidopsis thaliana)**	.	.	1.718206852	LRD
**Late embryogenesis abundant protein 76 (Brassica napus)**	1.533858234	1.877188535	3.861809893	Across all dehydration stages

**Late embryogenesis abundant protein D-29 (Gossypium hirsutum)**	1.5099243	2.576559174	2.865984352	Across all dehydration stages
**Late embryogenesis abundant protein D-34 (Gossypium hirsutum)**	2.07869733	1.933306564	3.090351727	Across all dehydration stages
**Late embryogenesis abundant protein Dc3 (Daucus carota)**	.	.	2.624853839	LRD
**LEA14-A Late embryogenesis abundant protein Lea14-A (Gossypium hirsutum)**	.	1.575799622	2.933865689	MRD and LRD
**LEA3 Late embryogenesis abundant protein, group 3 (Oryza sativa subsp. indica)**	.	1.310594012	2.660239854	MRD and LRD
**LTI65 Low-temperature-induced 65 kDa protein (Arabidopsis thaliana)**	.	2.00574943	3.1803771	MRD and LRD
**Major pollen allergen Cor a 1 isoforms 5, 6, 11 and 16 (Corylus avellana)**	.	.	1.245194023	LRD
**Mavicyanin (Cucurbita pepo)**	1.86719922	1.630580037	.	ERD and MRD
**MBF1B Multiprotein-bridging factor 1b (Arabidopsis thaliana)**	.	.	1.867719635	LRD
**MJ1232 Uncharacterized protein MJ1232 (Methanocaldococcus jannaschii (strain ATCC 43067 / DSM 2661 / JAL-1 / JCM 10045 / NBRC 100440))**	.	.	2.283632713	LRD
**MORF1 Multiple organellar RNA editing factor 1, mitochondrial (Arabidopsis thaliana)**	.	.	1.72895072	LRD
**MSRB5 Peptide methionine sulfoxide reductase B5 (Oryza sativa subsp. japonica)**	.	.	2.595009712	LRD
**MT4A Metallothionein-like protein 4A (Oryza sativa subsp. japonica)**	.	.	2.380677691	LRD
**MTA Metallothionein-like protein 1 (Pisum sativum)**	.	.	2.140799988	LRD
**MTH_273 UPF0098 protein MTH_273 (Methanothermobacter thermautotrophicus (strain ATCC 29096 / DSM 1053 / JCM 10044 / NBRC 100330 / Delta H))**	.	.	1.971340074	LRD
**NFS2 Cysteine desulfurase 1, chloroplastic (Arabidopsis thaliana)**	.	.	1.588145687	LRD
**Os01g0253300 Importin subunit alpha-1a (Oryza sativa subsp. japonica)**	.	.	2.079471181	LRD
**Os01g0270100 Cysteine proteinase inhibitor 12 (Oryza sativa subsp. japonica)**	.	.	1.291424614	LRD
**Os02g0194200 Zinc finger CCCH domain-containing protein 14 (Oryza sativa subsp. japonica)**	.	.	1.551029766	LRD

**Os02g0639900 Thioredoxin M1, chloroplastic (Oryza sativa subsp. japonica)**	.	.	1.351875046	LRD
**PAB8 Polyadenylate-binding protein 8 (Arabidopsis thaliana)**	.	.	1.246656116	LRD
**PCKR1 Peptidyl-prolyl cis-trans isomerase (Catharanthus roseus)**	.	.	1.26642199	LRD
**PED1 3-ketoacyl-CoA thiolase 2, peroxisomal (Arabidopsis thaliana)**	.	.	1.71951601	LRD
**Peptidyl-prolyl cis-trans isomerase (Lupinus luteus)**	.	.	0.90227811	LRD
**Phosphoglycerate kinase, chloroplastic (Nicotiana tabacum)**	.	.	0.426787502	LRD
**PHS2 Alpha-glucan phosphorylase 2, cytosolic (Arabidopsis thaliana)**	.	1.375643724	.	MRD
**PLP1 Patatin-like protein 1 (Oryza sativa subsp. japonica)**	1.552994529	1.416703369	1.306470985	Across all dehydration stages
**Polyphenol oxidase, chloroplastic (Vitis vinifera)**	0.950857782	.	0.729352191	ERD and LRD
**PORB Protochlorophyllide reductase B, chloroplastic (Hordeum vulgare)**	.	.	2.112431088	LRD
**PR1 Pathogenesis-related protein 1 (Asparagus officinalis)**	.	.	1.178761163	LRD
**PXG Peroxygenase (Oryza sativa subsp. japonica)**	.	.	1.63025003	LRD
**Pyruvate kinase, cytosolic isozyme (Glycine max)**	0.98750497	1.181922208	.	ERD and MRD
**RAB18 Dehydrin Rab18 (Arabidopsis thaliana)**	.	1.435795659	2.686911702	MRD and LRD
**RANBP1C Ran-binding protein 1 homolog c (Arabidopsis thaliana)**	.	.	1.826681471	LRD
**RBCS1 Ribulose bisphosphate carboxylase small chain, chloroplastic (Musa acuminata)**	.	.	0.884589514	LRD
**Remorin (Solanum tuberosum)**	.	.	1.760610351	LRD
**RPL23A 60S ribosomal protein L23 (Arabidopsis thaliana)**	.	.	1.200096286	LRD
**RPS12 40S ribosomal protein S12 (Hordeum vulgare)**	.	.	2.337496384	LRD
**RPS27B 40S ribosomal protein S27-2 (Arabidopsis thaliana)**	.	.	1.765478524	LRD
**RPT1 26S protease regulatory subunit 7 (Prunus persica)**	.	.	2.275208812	LRD
**RUB2 Ubiquitin-NEDD8-like protein RUB2 (Oryza sativa subsp. japonica)**	.	0.80210109	.	MRD
**RuBisCO large subunit-binding protein subunit beta, chloroplastic (Pisum sativum)**	.	.	0.637299581	LRD

**RZ1B Glycine-rich RNA-binding protein RZ1B (Arabidopsis thaliana)**	.	.	1.838550014	LRD
**SAPK7 Serine/threonine-protein kinase SAPK7 (Oryza sativa subsp. japonica)**	.	.	1.865952133	LRD
**SBT3.3 Subtilisin-like protease SBT3.3 (Arabidopsis thaliana)**	0.906750846	.	.	ERD
**SPG20 Spartin (Homo sapiens)**	1.383758686	2.136348103	3.216160058	Across all dehydration stages
**SYNPCC7002_A1590 Uncharacterized protein SYNPCC7002_A1590 (Synechococcus sp. (strain ATCC 27264 / PCC 7002 / PR-6))**	.	2.038698811	3.208950202	MRD and LRD
**TDX TPR repeat-containing thioredoxin TDX (Arabidopsis thaliana)**	.	.	1.272280451	LRD
**TMEM205 Transmembrane protein 205 (Homo sapiens)**	2.770021473	2.67668267	4.380864231	Across all dehydration stages
**TSJT1 Stem-specific protein TSJT1 (Nicotiana tabacum)**	.	.	1.611606585	LRD
**TUFB1 Elongation factor Tu, chloroplastic (Glycine max)**	.	.	0.984597415	LRD
**UGPA UTP--glucose-1-phosphate uridylyltransferase (Musa acuminata)**	.	0.654674746	.	MRD
**UGT78D2 UDP-glycosyltransferase 78D2 (Arabidopsis thaliana)**	1.920989868	2.561905006	2.78513244	Across all dehydration stages
**v1g171563 Eukaryotic translation initiation factor 3 subunit G (Nematostella vectensis)**	.	.	1.37568463	LRD

Table S5: Down-regulated proteins identified in *X. schlechteri* leaf tissue in response to dehydration at different dehydration stages. Early Response to Dehydration (ERD), Mid Response to Dehydration (MRD) and Late Response to Dehydration (LRD). The values represent the level of protein expression observed in each dehydration stage obtained from label-free quantitative proteomics data.

Protein Name	ERD	MRD	LRD	Dehydration stage
**14-3-3-like protein (Pisum sativum)**		-1.10726		MRD
**ACO2 Aconitate hydratase 2, mitochondrial (Arabidopsis thaliana)**			-1.25587	LRD
**ADG2 Glucose-1-phosphate adenylyltransferase large subunit 1, chloroplastic (Arabidopsis thaliana)**		-1.17966	-1.16885	MRD and LRD
**ADK-B Adenylate kinase 4 (Oryza sativa subsp. japonica)**	-1.44179	-1.37469		ERD and MRD
**AFRR Monodehydroascorbate reductase (Solanum lycopersicum)**			-1.00416	LRD
**AGD2 Probable LL-diaminopimelate aminotransferase, chloroplastic (Oryza sativa subsp. japonica)**			-1.77126	LRD
**ALATS Alanine--tRNA ligase (Arabidopsis thaliana)**			-1.49467	LRD
**Alpha-galactosidase (Coffea arabica)**	-1.07142	-1.17216	-1.0592	Across all dehydration stages
**Alpha-mannosidase (Canavalia ensiformis)**		-1.36506	-1.27805	MRD and LRD
**ANN1 Annexin D1 (Arabidopsis thaliana)**		-1.51742	-1.23012	MRD and LRD
**APX1 L-ascorbate peroxidase, cytosolic (Pisum sativum)**		-2.01611	-3.26533	MRD and LRD
**APX4 Probable L-ascorbate peroxidase 4 (Oryza sativa subsp. japonica)**		-1.42542		MRD
**APXT L-ascorbate peroxidase T, chloroplastic (Arabidopsis thaliana)**		-2.14943		MRD
**At1g09760 U2 small nuclear ribonucleoprotein A' (Arabidopsis thaliana)**		-1.75195		MRD
**At1g62810 Primary amine oxidase (Arabidopsis thaliana)**			-2.47528	LRD
**At1g67280 Probable lactoylglutathione lyase, chloroplast (Arabidopsis thaliana)**		-1.59699		MRD
**At2g04400 Indole-3-glycerol phosphate synthase, chloroplastic (Arabidopsis thaliana)**		-1.87518		MRD
**At2g21870 Probable ATP synthase 24 kDa subunit, mitochondrial (Arabidopsis thaliana)**		-0.93448		MRD
**At3g10130 Heme-binding-like protein At3g10130, chloroplastic (Arabidopsis thaliana)**		-1.84486		MRD

**At3g48420 Haloacid dehalogenase-like hydrolase domain-containing protein At3g48420 (Arabidopsis thaliana)**		-1.65737		MRD
**At3g63540 Thylakoid lumenal 19 kDa protein, chloroplastic (Arabidopsis thaliana)**		-1.21618		MRD
**At4g13360 3-hydroxyisobutyryl-CoA hydrolase-like protein 3, mitochondrial (Arabidopsis thaliana)**			-1.4925	LRD
**At4g26910 Dihydrolipoyllysine-residue succinyltransferase component of 2-oxoglutarate dehydrogenase complex 2, mitochondrial (Arabidopsis thaliana)**	-0.89439	-1.00452		ERD and MRD
**ATP synthase subunit delta', mitochondrial (Ipomoea batatas)**		-1.85971		MRD
**Basic secretory protease (Fragments) (Boswellia serrata)**	-1.6699	-1.48199	-2.38693	Across all dehydration stages
**Beta-galactosidase (Asparagus officinalis)**		-1.86712	-1.24451	MRD and LRD
**BGAL9 Beta-galactosidase 9 (Arabidopsis thaliana)**		-1.1721		MRD
**BGLU22 Beta-glucosidase 22 (Oryza sativa subsp. japonica)**			-1.47715	LRD
**caa43 2-haloacrylate reductase (Burkholderia sp.)**			-1.66697	LRD
**CAB13 Chlorophyll a-b binding protein 13, chloroplastic (Solanum lycopersicum)**		-1.98118	-3.54613	MRD and LRD
**CAB21 Chlorophyll a-b binding protein 21, chloroplastic (Nicotiana tabacum)**		-1.51935	-2.68394	MRD and LRD
**CAB36 Chlorophyll a-b binding protein 36, chloroplastic (Nicotiana tabacum)**		-1.0662	-2.82067	MRD and LRD
**CAB6A Chlorophyll a-b binding protein 6A, chloroplastic (Solanum lycopersicum)**		-1.04674	-3.1886	MRD and LRD
**CAB7 Chlorophyll a-b binding protein 7, chloroplastic (Solanum lycopersicum)**			-1.71514	LRD
**CAB8 Chlorophyll a-b binding protein 8, chloroplastic (Solanum lycopersicum)**			-2.88429	LRD
**CAP10A Chlorophyll a-b binding protein CP24 10A, chloroplastic (Solanum lycopersicum)**		-1.68796	-1.89943	MRD and LRD
**CAT1 Catalase-1 (Triticum aestivum)**		-0.86209		MRD
**CEBIP Chitin elicitor-binding protein (Oryza sativa subsp. japonica)**			-1.27643	LRD
**CEST Protein CHLOROPLAST ENHANCING STRESS TOLERANCE, chloroplastic (Oryza sativa subsp. japonica)**		-1.61931		MRD
**CHI4 Endochitinase PR4 (Phaseolus vulgaris)**	-1.70233		-1.95186	ERD and LRD

**CHLM Magnesium protoporphyrin IX methyltransferase, chloroplastic (Arabidopsis thaliana)**	-1.54851			ERD
**Chlorophyll a-b binding protein, chloroplastic (Spinacia oleracea)**		-1.59118	-2.75709	MRD and LRD
**CHLP Geranylgeranyl diphosphate reductase, chloroplastic (Nicotiana tabacum)**	-1.54227	-1.87164	-1.88202	Across all dehydration stages
**CLPP5 ATP-dependent Clp protease proteolytic subunit 5, chloroplastic (Arabidopsis thaliana)**		-1.52476		MRD
**CNX1 Calnexin homolog 1 (Arabidopsis thaliana)**		-1.57911		MRD
**CPN21 20 kDa chaperonin, chloroplastic (Arabidopsis thaliana)**		-1.30291		MRD
**CSP41A Chloroplast stem-loop binding protein of 41 kDa a, chloroplastic (Arabidopsis thaliana)**		-1.19802		MRD
**CSP41B Chloroplast stem-loop binding protein of 41 kDa b, chloroplastic (Arabidopsis thaliana)**		-0.94221		MRD
**CURT1A Protein CURVATURE THYLAKOID 1A, chloroplastic (Arabidopsis thaliana)**		-1.5739		MRD
**CURT1D Protein CURVATURE THYLAKOID 1D, chloroplastic (Arabidopsis thaliana)**		-1.78231		MRD
**CYCL Cytochrome c1-1, heme protein, mitochondrial (Solanum tuberosum)**		-1.69274		MRD
**CYP37 Peptidyl-prolyl cis-trans isomerase CYP37, chloroplastic (Arabidopsis thaliana)**		-1.5542		MRD
**CYP74B2 Linolenate hydroperoxide lyase, chloroplastic (Arabidopsis thaliana)**			-1.25374	LRD
**dapb3 Dipeptidyl aminopeptidase BIII (Pseudoxanthomonas mexicana)**			-1.62806	LRD
**EBM Mannosylglycoprotein endo-beta-mannosidase (Lilium longiflorum)**			-1.06139	LRD
**EDA2 Probable serine protease EDA2 (Arabidopsis thaliana)**			-1.40294	LRD
**Ephx2 Bifunctional epoxide hydrolase 2 (Mus musculus)**		-1.62283		MRD
**FBPban1 Fructose-1,6-bisphosphatase, cytosolic (Musa acuminata)**		-1.55921		MRD
**FKBP19 Peptidyl-prolyl cis-trans isomerase FKBP19, chloroplastic (Arabidopsis thaliana)**		-1.65151		MRD
**FKBP62 Peptidyl-prolyl cis-trans isomerase FKBP62 (Arabidopsis thaliana)**		-1.4288		MRD

**GAPC2 Glyceraldehyde-3-phosphate dehydrogenase 2, cytosolic (Oryza sativa subsp. japonica)**	-0.73304			ERD
**GGAT1 Glutamate--glyoxylate aminotransferase 1 (Arabidopsis thaliana)**		-2.4394		MRD
**GGH2 Gamma-glutamyl hydrolase 2 (Arabidopsis thaliana)**		-1.31007		MRD
**GLU Ferredoxin-dependent glutamate synthase, chloroplastic (Oryza sativa subsp. japonica)**			-1.80357	LRD
**Glucan endo-1,3-beta-glucosidase, acidic isoform (Zea mays)**	-2.188			ERD
**GOR Glutathione reductase, chloroplastic (Fragment) (Nicotiana tabacum)**			-1.21042	LRD
**HEMC Porphobilinogen deaminase, chloroplastic (Oryza sativa subsp. japonica)**	-1.08601	-1.46669		ERD and MRD
**HIR1 Hypersensitive-induced response protein 1 (Arabidopsis thaliana)**		-1.87411		MRD
**HIR1 Hypersensitive-induced response protein 1 (Arabidopsis thaliana)**			-1.80702	LRD
**HSP90 Endoplasmin homolog (Catharanthus roseus)**	-1.16691			ERD
**IBI1 Aspartate--tRNA ligase 2, cytoplasmic (Arabidopsis thaliana)**			-1.55808	LRD
**IMPL1 Phosphatase IMPL1, chloroplastic (Arabidopsis thaliana)**		-1.75618		MRD
**infC Translation initiation factor IF-3 (Nostoc sp. (strain PCC 7120 / UTEX 2576))**		-1.50504		MRD
**LHCA1 Chlorophyll a-b binding protein 6, chloroplastic (Arabidopsis thaliana)**		-1.35192	-2.65519	MRD and LRD
**LHCA4 Chlorophyll a-b binding protein 4, chloroplastic (Arabidopsis thaliana)**		-1.45625	-2.63069	MRD and LRD
**LHCB4.2 Chlorophyll a-b binding protein CP29.2, chloroplastic (Arabidopsis thaliana)**		-1.00218	-3.71008	MRD and LRD
**LHCB5 Chlorophyll a-b binding protein CP26, chloroplastic (Arabidopsis thaliana)**		-1.46518	-3.18151	MRD and LRD
**ligA DNA ligase (Bradyrhizobium sp. (strain ORS278))**			-1.86947	LRD
**LOX2.1 Linoleate 13S-lipoxygenase 2-1, chloroplastic (Solanum tuberosum)**			-2.25474	LRD
**LSF1 Phosphoglucan phosphatase LSF1, chloroplastic (Arabidopsis thaliana)**		-1.30958		MRD
**NAD-ME1 NAD-dependent malic enzyme 1, mitochondrial (Arabidopsis thaliana)**		-1.668	-1.65009	MRD and LRD
**OEP21B Outer envelope pore protein 21B, chloroplastic (Arabidopsis thaliana)**			-1.43681	LRD

**Os01g0895100 Probable membrane-associated 30 kDa protein, chloroplastic (Oryza sativa subsp. japonica)**		-1.44434		MRD
**Os02g0739600 Pyruvate dehydrogenase E1 component subunit alpha-1, mitochondrial (Oryza sativa subsp. japonica)**			-1.4089	LRD
**Os06g0675700 Probable alpha-glucosidase Os06g0675700 (Oryza sativa subsp. japonica)**	-1.26142	-1.86095	-1.70842	Across all dehydration stages
**Os08g0242700 ACT domain-containing protein DS12, chloroplastic (Oryza sativa subsp. japonica)**		-1.61348		MRD
**Os08g0288200 Probable adenylate kinase 5, chloroplastic (Oryza sativa subsp. japonica)**	-1.53706	-1.61952	-1.57662	Across all dehydration stages
**Os08g0562700 Puromycin-sensitive aminopeptidase (Oryza sativa subsp. japonica)**			-0.89965	LRD
**Os09g0529100 Probable 6-phosphogluconolactonase 4, chloroplastic (Oryza sativa subsp. japonica)**	-1.90522			ERD
**PAB1 Proteasome subunit alpha type-2 (Oryza sativa subsp. japonica)**		-1.36335		MRD
**PAP2 Probable inactive purple acid phosphatase 2 (Arabidopsis thaliana)**			-1.20063	LRD
**PAP27 Probable inactive purple acid phosphatase 27 (Arabidopsis thaliana)**		-1.5832		MRD
**PAP27 Probable inactive purple acid phosphatase 27 (Arabidopsis thaliana)**			-2.07959	LRD
**PBF1 Proteasome subunit beta type-1 (Oryza sativa subsp. japonica)**			-1.19524	LRD
**PBG1 Proteasome subunit beta type-4 (Arabidopsis thaliana)**		-1.36101		MRD
**Peptide-N4-(N-acetyl-beta-glucosaminyl)asparagine amidase A (Prunus dulcis)**		-1.5359	-1.48744	MRD and LRD
**petC Cytochrome b6-f complex iron-sulfur subunit, chloroplastic (Oryza sativa subsp. japonica)**	-1.19338	-1.35424		ERD and MRD
**PETE Plastocyanin minor isoform, chloroplastic (Arabidopsis thaliana)**		-1.26885		MRD
**PETH Ferredoxin--NADP reductase, leaf isozyme, chloroplastic (Pisum sativum)**		-0.9405		MRD
**PGMP Phosphoglucomutase, chloroplastic (Solanum tuberosum)**		-1.38242		MRD

**PME35 Probable pectinesterase/pectinesterase inhibitor 35 (Arabidopsis thaliana)**			-1.40972	LRD
**PNSL1 Photosynthetic NDH subunit of lumenal location 1, chloroplastic (Arabidopsis thaliana)**	-1.62881	-2.05822		ERD and MRD
**POPTRDRAFT_831870 Biotin carboxylase 1, chloroplastic (Populus trichocarpa)**			-1.77974	LRD
**PORB Protochlorophyllide reductase B, chloroplastic (Hordeum vulgare)**	-1.80876			ERD
**PPL1 PsbP-like protein 1, chloroplastic (Arabidopsis thaliana)**		-1.11766		MRD
**PREP1 Presequence protease 1, chloroplastic/mitochondrial (Arabidopsis thaliana)**			-1.44761	LRD
**Proteasome subunit beta type-5 (Spinacia oleracea)**			-1.27071	LRD
**psaD Photosystem I reaction center subunit II, chloroplastic (Cucumis sativus)**			-1.3584	LRD
**PSAD2 Photosystem I reaction center subunit II-2, chloroplastic (Arabidopsis thaliana)**			-1.28325	LRD
**PSAF Photosystem I reaction center subunit III, chloroplastic (Arabidopsis thaliana)**			-1.32661	LRD
**PSAG Photosystem I reaction center subunit V, chloroplastic (Arabidopsis thaliana)**			-2.25576	LRD
**PSAH1 Photosystem I reaction center subunit VI-1, chloroplastic (Arabidopsis thaliana)**		-1.55564		MRD
**PSAH1 Photosystem I reaction center subunit VI-1, chloroplastic (Arabidopsis thaliana)**			-2.7504	LRD
**PSAH2 Photosystem I reaction center subunit VI-2, chloroplastic (Arabidopsis thaliana)**			-2.32278	LRD
**PSAL Photosystem I reaction center subunit XI, chloroplastic (Hordeum vulgare)**			-2.04454	LRD
**PSAN Photosystem I reaction center subunit N, chloroplastic (Arabidopsis thaliana)**			-2.69906	LRD
**PSBO Oxygen-evolving enhancer protein 1, chloroplastic (Solanum lycopersicum)**		-1.27835	-1.09965	MRD and LRD
**PSBP Oxygen-evolving enhancer protein 2, chloroplastic (Pisum sativum)**		-1.3232		MRD
**PSBP Oxygen-evolving enhancer protein 2, chloroplastic (Pisum sativum)**			-1.77582	LRD
**PSBR Photosystem II 10 kDa polypeptide, chloroplastic (Solanum tuberosum)**			-2.69774	LRD
**PXG4 Probable peroxygenase 4 (Arabidopsis thaliana)**		-1.81513		MRD

**RABA2A Ras-related protein RABA2a (Arabidopsis thaliana)**	-1.62736	-1.61228	-1.45266	Across all dehydration stages
**RBCS1 Ribulose bisphosphate carboxylase small chain, chloroplastic (Musa acuminata)**		-0.84364		MRD
**RCA1 Ribulose bisphosphate carboxylase/oxygenase activase 1, chloroplastic (Larrea tridentata)**		-0.90161		MRD
**RCA2 Ribulose bisphosphate carboxylase/oxygenase activase 2, chloroplastic (Larrea tridentata)**		-1.41565		MRD
**Remorin (Solanum tuberosum)**		-1.21839		MRD
**RFS Galactinol--sucrose galactosyltransferase (Oryza sativa subsp. japonica)**	-1.69881	-2.55586	-2.80097	Across all dehydration stages
**RPL3 60S ribosomal protein L3 (Oryza sativa subsp. japonica)**		-1.41616		MRD
**RPL4 50S ribosomal protein L4, chloroplastic (Nicotiana tabacum)**		-1.75638		MRD
**RPL5 50S ribosomal protein L5, chloroplastic (Arabidopsis thaliana)**	-0.93784	-1.84237		ERD and MRD
**RPL8 60S ribosomal protein L8 (Solanum lycopersicum)**		-1.48883		MRD
**RPL9 50S ribosomal protein L9, chloroplastic (Arabidopsis thaliana)**		-1.55571		MRD
**RPN8A 26S proteasome non-ATPase regulatory subunit 7 homolog A (Arabidopsis thaliana)**	-1.47507			ERD
**RPS17 40S ribosomal protein S17 (Solanum lycopersicum)**		-2.25057		MRD
**RPS2C 40S ribosomal protein S2-3 (Arabidopsis thaliana)**		-1.16438		MRD
**rps5 30S ribosomal protein S5, chloroplastic (Arabidopsis thaliana)**		-1.46291		MRD
**SAG12 Senescence-specific cysteine protease SAG12 (Arabidopsis thaliana)**			-1.47142	LRD
**Serine hydroxymethyltransferase, mitochondrial (Solanum tuberosum)**		-0.61287		MRD
**Serpin-Z1C (Triticum aestivum)**			-1.13413	LRD
**SPAC9E9.15 Uncharacterized protein C9E9.15 (Schizosaccharomyces pombe (strain 972 / ATCC 24843))**		-1.10348	-1.52577	MRD and LRD
**tal Transaldolase (Shewanella loihica (strain ATCC BAA-1088 / PV-4))**	-1.73548			ERD

**TCTP Translationally-controlled tumor protein homolog (Elaeis guineensis var. tenera)**	-1.25771	-1.59903		ERD and MRD
**THI1-2 Thiamine thiazole synthase 2, chloroplastic (Vitis vinifera)**			-1.21078	LRD
**TIC62 Protein TIC 62, chloroplastic (Arabidopsis thaliana)**		-1.77576		MRD
**tolB Protein TolB (Geobacter metallireducens (strain GS-15 / ATCC 53774 / DSM 7210))**		-1.63042	-1.53492	MRD and LRD
**TP53I3 Quinone oxidoreductase PIG3 (Homo sapiens)**	-1.5504		-1.55183	ERD and LRD
**TPIP1 Triosephosphate isomerase, chloroplastic (Spinacia oleracea)**		-1.13924		MRD
**Transketolase, chloroplastic (Solanum tuberosum)**		-1.00238	-1.01196	MRD and LRD
**Triosephosphate isomerase, chloroplastic (Secale cereale)**		-1.54031		MRD
**UAM1 UDP-arabinopyranose mutase 1 (Oryza sativa subsp. japonica)**			-1.79507	LRD
**UVR8 Ultraviolet-B receptor UVR8 (Arabidopsis thaliana)**	-1.56712	-1.77932	-1.63009	Across all dehydration stages
**Vacuolar-processing enzyme (Citrus sinensis)**	-1.48109	-1.87333	-2.42145	Across all dehydration stages
**VIT_19s0014g02480 Probable bifunctional methylthioribulose-1-phosphate dehydratase/enolase-phosphatase E1 1 (Vitis vinifera)**		-1.45087	-1.39129	MRD and LRD
**WAXY Granule-bound starch synthase 1, chloroplastic/amyloplastic (Antirrhinum majus)**			-1.8444	LRD
**zta1 Probable quinone oxidoreductase (Schizosaccharomyces pombe (strain 972 / ATCC 24843))**			-1.81277	LRD
**Keratin, type I cytoskeletal 9 - Homo sapiens (Human).**	-1.58735		-2.07128	ERD and LRD
**Protein of unknown function**	-1.86452			ERD
**Protein of unknown function**	-1.59024			ERD
**Serum albumin OS=Bos taurus GN=ALB PE=1 SV=4**	-2.02522	-1.78496	-2.07012	Across all dehydration stages

Table S6: List of *Xerophyta schlechteri* unknown proteins identified during label-free proteomics. InterPro (https://www.ebi.ac.uk/interpro/) was used to find GO-terms and function of the unknown protein where possible.

X. schlechteri* ID	**Description**	**GO-Term**	**Function**
Xvis03_200013-PA	processed-gene-1.89-mRNA-1 protein Name:"Protein of unknown function" AED:0.15 eAED:0.15 QI:0|-1|0|1|-1|1|1|0|178	P:GO:0009408; P:GO:0010115	P:response to heat; P:regulation of abscisic acid biosynthetic process
Xvis03_200377-PA	processed-gene-47.99-mRNA-1 protein Name:"Protein of unknown function" AED:0.68 eAED:0.68 QI:0|-1|0|1|-1|1|1|0|186	P:GO:0009408; P:GO:0010115	P:response to heat; P:regulation of abscisic acid biosynthetic process
Xvis03_200562-PA	processed-gene-71.17-mRNA-1 protein Name:"Protein of unknown function" AED:0.29 eAED:0.29 QI:199|1|1|1|1|1|5|271|381	F:GO:0005509	F:calcium ion binding
Xvis03_202413-PA	processed-gene-34.23-mRNA-1 protein Name:"Protein of unknown function" AED:0.01 eAED:0.01 QI:0|-1|0|1|-1|1|1|0|140	C:GO:0005783; P:GO:0006886; C:GO:0016021	C:endoplasmic reticulum; P:intracellular protein transport; C:integral component of membrane
Xvis03_204624-PA	augustus-gene-37.61-mRNA-1 protein Name:"Protein of unknown function" AED:0.00 eAED:0.00 QI:188|1|1|1|1|1|5|376|359	F:GO:0003723	F:RNA binding
Xvis03_205607-PA	augustus-gene-18.67-mRNA-1 protein Name:"Protein of unknown function" AED:0.34 eAED:0.34 QI:138|1|1|1|1|1|4|447|216	P:GO:0000724; F:GO:0003677	P:double-strand break repair via homologous recombination; F:DNA binding
Xvis03_206711-PA	augustus-gene-20.17-mRNA-1 protein Name:"Protein of unknown function" AED:0.00 eAED:0.00 QI:112|1|1|1|1|1|2|153|186	F:GO:0005515	F:protein binding
Xvis03_206781-PA	processed-gene-26.3-mRNA-1 protein Name:"Protein of unknown function" AED:0.19 eAED:0.19 QI:331|1|1|1|1|1|2|174|193	F:GO:0005515	F:protein binding
Xvis03_206790-PA	processed-gene-27.3-mRNA-1 protein Name:"Protein of unknown function" AED:0.52 eAED:0.52 QI:0|0.7|0.72|0.72|1|1|11|0|294	F:GO:0005515	F:protein binding
Xvis03_209030-PA	processed-gene-10.24-mRNA-1 protein Name:"Protein of unknown function"	F:GO:0003743	F:translation initiation factor activity

	AED:0.25 eAED:0.25 QI:87|0|0.5|0.5|1|1|4|0|556		
Xvis03_209250-PA	augustus-gene-6.35-mRNA-1 protein Name:"Protein of unknown function" AED:0.09 eAED:0.09 QI:9|1|1|1|0.83|1|7|549|267	F:GO:0005509; C:GO:0009654; P:GO:0015979; C:GO:0019898	F:calcium ion binding; C:photosystem II oxygen evolving complex; P:photosynthesis; C:extrinsic component of membrane
Xvis03_209551-PA	augustus-gene-8.71-mRNA-1 protein Name:"Protein of unknown function" AED:0.00 eAED:0.00 QI:115|1|1|1|1|1|3|283|52	C:GO:0000276; F:GO:0015078; P:GO:0015986	C:mitochondrial proton-transporting ATP synthase complex, coupling factor F(o); F:proton transmembrane transporter activity; P:ATP synthesis coupled proton transport
Xvis03_211981-PA	processed-gene-13.88-mRNA-1 protein Name:"Protein of unknown function" AED:0.39 eAED:0.39 QI:90|0.33|0.25|0.5|1|1|4|0|156	F:GO:0004867; C:GO:0005576	F:serine-type endopeptidase inhibitor activity; C:extracellular region
Xvis03_212079-PA	augustus-gene-19.87-mRNA-1 protein Name:"Protein of unknown function" AED:0.24 eAED:0.24 QI:126|1|0.90|1|0.6|0.45|11|424|1275	F:GO:0005515	F:protein binding
Xvis03_212518-PA	augustus-gene-6.13-mRNA-1 protein Name:"Protein of unknown function" AED:0.14 eAED:0.14 QI:93|1|0.5|1|1|1|2|0|537	F:GO:0003743	F:translation initiation factor activity
Xvis03_212553-PA	augustus-gene-10.40-mRNA-1 protein Name:"Protein of unknown function" AED:0.16 eAED:0.16 QI:74|0.92|0.92|1|0.92|0.92|14|451|363	P:GO:1900034	P:regulation of cellular response to heat
Xvis03_212807-PA	processed-gene-7.89-mRNA-1 protein Name:"Protein of unknown function" AED:0.10 eAED:0.10 QI:219|0.91|0.84|1|0.91|0.92|13|567|841	P:GO:0046373; F:GO:0046556	P:L-arabinose metabolic process; F:alpha-L-arabinofuranosidase activity
Xvis03_213271-PA	augustus-gene-4.72-mRNA-1 protein Name:"Protein of unknown function" AED:0.03 eAED:0.03 QI:529|1|1|1|1|1|5|472|382	F:GO:0005509	F:calcium ion binding

Xvis03_213561-PA	augustus-gene-11.61-mRNA-1 protein Name:"Protein of unknown function" AED:0.05 eAED:0.05 QI:138|0.5|0.6|1|1|1|5|352|338	F:GO:0003723	F:RNA binding
Xvis03_214461-PA	augustus-gene-14.50-mRNA-1 protein Name:"Protein of unknown function" AED:0.25 eAED:0.25 QI:166|1|1|1|0.88|0.9|10|542|839	F:GO:0005515	F:protein binding
Xvis03_214462-PA	augustus-gene-14.52-mRNA-1 protein Name:"Protein of unknown function" AED:0.33 eAED:0.37 QI:0|0.72|0.66|0.83|1|1|12|303|933	F:GO:0005515	F:protein binding
Xvis03_214558-PA	processed-gene-7.54-mRNA-1 protein Name:"Protein of unknown function" AED:0.02 eAED:0.02 QI:170|1|1|1|1|1|6|290|375	F:GO:0003723	F:RNA binding
Xvis03_214692-PA	processed-gene-2.8-mRNA-1 protein Name:"Protein of unknown function" AED:0.04 eAED:0.04 QI:39|1|1|1|1|1|4|584|183	P:GO:0000724; F:GO:0003677	P:double-strand break repair via homologous recombination; F:DNA binding
Xvis03_214790-PA	processed-gene-10.70-mRNA-1 protein Name:"Protein of unknown function" AED:0.08 eAED:0.08 QI:0|0|0|1|1|1|4|0|183	P:GO:0000724; F:GO:0003677	P:double-strand break repair via homologous recombination; F:DNA binding
Xvis03_214817-PA	processed-gene-12.12-mRNA-1 protein Name:"Protein of unknown function" AED:0.22 eAED:0.22 QI:0|0.5|0.55|1|1|1|9|386|285	F:GO:0005515	F:protein binding
Xvis03_215263-PA	processed-gene-5.90-mRNA-1 protein Name:"Protein of unknown function" AED:0.07 eAED:0.07 QI:25|1|1|1|1|1|3|199|124	C:GO:0009522; P:GO:0015979	C:photosystem I; P:photosynthesis
Xvis03_215636-PA	augustus-gene-11.1-mRNA-1 protein Name:"Protein of unknown function" AED:0.06 eAED:0.06 QI:45|0.87|0.88|1|0.87|0.88|9|510|884	F:GO:0005515	F:protein binding
Xvis03_216099-PA	processed-gene-0.4-mRNA-1 protein Name:"Protein of unknown function" AED:0.04 eAED:0.04 QI:300|0.5|1|1|1|1|3|3437|201	P:GO:0000724; F:GO:0003677	P:double-strand break repair via homologous recombination; F:DNA binding

Xvis03_217664-PA	augustus-gene-4.63-mRNA-1 protein Name:"Protein of unknown function" AED:0.25 eAED:0.25 QI:465|0.8|0.81|0.90|1|1|11|14|581	F:GO:0003824	F:catalytic activity
Xvis03_217665-PA	est_gff_Cufflinks-gene-4.45-mRNA-1 protein Name:"Protein of unknown function" AED:0.33 eAED:0.33 QI:81|1|1|1|0|0.5|2|546|253	P:GO:0046373; F:GO:0046556	P:L-arabinose metabolic process; F:alpha-L-arabinofuranosidase activity
Xvis03_218355-PA	augustus-gene-4.74-mRNA-1 protein Name:"Protein of unknown function" AED:0.11 eAED:0.11 QI:176|0.66|0.85|1|0.83|1|7|900|405	F:GO:0003723	F:RNA binding
Xvis03_219348-PA	augustus-gene-3.41-mRNA-1 protein Name:"Protein of unknown function" AED:0.07 eAED:0.07 QI:66|1|1|1|1|1|2|366|422	F:GO:0003743	F:translation initiation factor activity
Xvis03_219634-PA	processed-gene-3.2-mRNA-1 protein Name:"Protein of unknown function" AED:0.00 eAED:0.00 QI:123|1|1|1|1|1|3|533|203	P:GO:0010183; F:GO:0036033	P:pollen tube guidance; F:mediator complex binding
Xvis03_220523-PA	augustus-gene-3.91-mRNA-1 protein Name:"Protein of unknown function" AED:0.02 eAED:0.02 QI:101|1|1|1|0.5|0.33|3|285|67		
Xvis03_220526-PA	augustus-gene-4.0-mRNA-1 protein Name:"Protein of unknown function" AED:0.61 eAED:0.63 QI:97|0.25|0.46|0.53|0.66|0.84|13|0|505		
Xvis03_220783-PA	processed-gene-7.30-mRNA-1 protein Name:"Protein of unknown function" AED:0.03 eAED:0.03 QI:54|1|1|1|0.77|0.9|10|270|997		
Xvis03_220839-PA	augustus-gene-7.57-mRNA-1 protein Name:"Protein of unknown function" AED:0.15 eAED:0.18 QI:0|0|0|0.85|1|0.85|7|0|1206		
Xvis03_221114-PA	augustus-gene-7.153-mRNA-1 protein Name:"Protein of unknown function" AED:0.31 eAED:0.31 QI:37|0.81|0.91|0.91|0.90|0.91|12|0|389	F:GO:0005515	F:protein binding

Xvis03_221241-PA	processed-gene-5.4-mRNA-1 protein Name:"Protein of unknown function" AED:0.08 eAED:0.08 QI:245|0.2|0.66|0.66|1|1|6|196|274		
Xvis03_221245-PA	augustus-gene-5.32-mRNA-1 protein Name:"Protein of unknown function" AED:0.09 eAED:0.09 QI:132|0.75|0.8|1|0.5|0.8|5|329|209		
Xvis03_221584-PA	augustus-gene-3.67-mRNA-1 protein Name:"Protein of unknown function" AED:0.09 eAED:0.09 QI:0|1|0.88|1|1|1|9|315|844		
Xvis03_221586-PA	est_gff_Cufflinks-gene-3.18-mRNA-1 protein Name:"Protein of unknown function" AED:0.00 eAED:0.00 QI:74|1|1|1|0|0|2|161|102		
Xvis03_221803-PA	processed-gene-5.17-mRNA-1 protein Name:"Protein of unknown function" AED:0.41 eAED:0.41 QI:0|0.5|0.33|0.66|1|1|3|324|192		
Xvis03_221874-PA	processed-gene-3.62-mRNA-1 protein Name:"Protein of unknown function" AED:0.05 eAED:0.05 QI:40|1|1|1|1|1|3|57|223		
Xvis03_222141-PA	processed-gene-2.132-mRNA-1 protein Name:"Protein of unknown function" AED:0.05 eAED:0.05 QI:31|1|1|1|1|1|5|508|372		
Xvis03_222453-PA	augustus-gene-5.66-mRNA-1 protein Name:"Protein of unknown function" AED:0.10 eAED:0.10 QI:170|1|1|1|1|1|5|279|183		
Xvis03_222491-PA	augustus-gene-2.15-mRNA-1 protein Name:"Protein of unknown function" AED:0.03 eAED:0.03 QI:12|1|1|1|1|1|4|441|657		
Xvis03_222633-PA	processed-gene-2.6-mRNA-1 protein Name:"Protein of unknown function" AED:0.00 eAED:0.00 QI:74|1|1|1|1|1|2|188|155		

Xvis03_223073-PA	processed-gene-1.5-mRNA-1 protein Name:"Protein of unknown function" AED:0.19 eAED:0.19 QI:0|0.5|0.33|0.66|1|1|3|248|338		
Xvis03_223402-PA	processed-gene-3.113-mRNA-1 protein Name:"Protein of unknown function" AED:0.15 eAED:0.15 QI:0|-1|0|1|-1|1|1|0|200		
Xvis03_223551-PA	augustus-gene-0.4-mRNA-1 protein Name:"Protein of unknown function" AED:0.05 eAED:0.05 QI:85|0.66|1|1|1|1|4|290|185		
Xvis03_223700-PA	processed-gene-0.36-mRNA-1 protein Name:"Protein of unknown function" AED:0.07 eAED:0.07 QI:74|0|0.66|0.66|1|1|3|231|183		
Xvis03_223934-PA	processed-gene-3.6-mRNA-1 protein Name:"Protein of unknown function" AED:0.65 eAED:0.65 QI:0|0.28|0.12|0.5|1|1|8|0|363		
Xvis03_224176-PA	processed-gene-0.70-mRNA-1 protein Name:"Protein of unknown function" AED:0.46 eAED:0.46 QI:0|-1|0|1|-1|1|1|0|84		
Xvis03_224299-PA	processed-gene-1.45-mRNA-1 protein Name:"Protein of unknown function" AED:0.17 eAED:0.17 QI:0|-1|0|1|-1|1|1|0|442		
Xvis03_224443-PA	processed-gene-2.98-mRNA-1 protein Name:"Protein of unknown function" AED:0.37 eAED:0.42 QI:0|0|0|1|1|1|2|0|148		
Xvis03_224539-PA	processed-gene-2.15-mRNA-1 protein Name:"Protein of unknown function" AED:0.34 eAED:0.34 QI:0|1|0.8|1|1|1|5|142|253		
Xvis03_224748-PA	augustus-gene-0.35-mRNA-1 protein Name:"Protein of unknown function" AED:0.10 eAED:0.10 QI:42|0.5|1|1|0.5|0.66|3|247|139		
Xvis03_224886-PA	augustus-gene-0.35-mRNA-1 protein Name:"Protein of unknown function" AED:0.15 eAED:0.15 QI:0|1|0.83|1|1|1|6|796|313		

Xvis03_224919-PA	processed-gene-1.1-mRNA-1 protein Name:"Protein of unknown function" AED:0.11 eAED:0.11 QI:31|1|1|1|1|1|4|3638|250		
Xvis03_225115-PA	augustus-gene-0.198-mRNA-1 protein Name:"Protein of unknown function" AED:0.20 eAED:0.11 QI:39|0.71|0.75|1|1|0.87|8|1386|134		
Xvis03_225434-PA	augustus-gene-0.49-mRNA-1 protein Name:"Protein of unknown function" AED:0.01 eAED:0.01 QI:74|1|1|1|1|1|2|209|143		
Xvis03_225567-PA	processed-gene-0.22-mRNA-1 protein Name:"Protein of unknown function" AED:0.19 eAED:0.19 QI:0|0.16|0.14|0.71|1|1|7|86|485		
Xvis03_225590-PA	augustus-gene-0.1-mRNA-1 protein Name:"Protein of unknown function" AED:0.00 eAED:0.00 QI:185|1|1|1|1|1|2|42|261		
Xvis03_225702-PA	processed-gene-0.52-mRNA-1 protein Name:"Protein of unknown function" AED:0.28 eAED:0.28 QI:29|0.77|0.9|1|1|1|10|29|322		

**X. schlechteri* database version 3

Table S7: Mapman BINs representing upregulated and downregulated *X. schlechteri* proteins at early response to drying.

Bin	Name	Elements	p-value

	**Upregulated proteins at ERD**	**(no.of proteins allocated)**	
35	Not assigned	11	0.676053
9	Secondary metabolism	2	0.791296
1	Photosynthesis	1	0.140458
4	Amino acid metabolism	1	0.140458
11	Phytohormone action	1	0.259640
25	Nutrient uptake	1	0.259640
5	Lipid metabolism	1	0.435156
2	Cellular respiration	1	0.664612
**Bin**	**Name Downregulated proteins at ERD**	**Elements (no.of proteins allocated)**	**p-value**
7	Co-enzyme metabolism	5	0.785817
19	Protein homeostasis	4	0.335234
35	Not assigned	3	0.276986
3	Carbohydrate metabolism	2	0.045141
1	Photosynthesis	2	0.920212
6	Nucleotide metabolism	2	0.920212
2	Cellular respiration	1	0.127153
17	Protein biosynthesis	1	0.165518
22	Vesicle trafficking	1	0.405381
26	External stimuli response	1	0.488074
27	Multi-process regulation	1	1

Table S8: Mapman BINs representing upregulated and downregulated *X. schlechteri* proteins at Mid response to drying.

**Bin**	**Name Upregulated proteins at MRD**	**Elements (no.of proteins allocated)**	**p-value**
35	Not assigned	2	0.029297
19	Protein homeostasis	2	0.028108
3	Carbohydrate metabolism	2	0.107347
5	Lipid metabolism	2	0.941653
9	Secondary metabolism	1	0.114128
12	Chromatin organisation	1	0.202622
2	Cellular respiration	1	0.332880
17	Protein biosynthesis	1	0.798859
16	RNA processing	1	0.878482
26	External stimuli response	1	0.878482

Bin	Name<br>**Downregulated proteins at MRD**	Elements (no.of proteins allocated)	p-value
1	Photosynthesis	27	2.71E-04
35	Not assigned	16	0.481927
17	Protein biosynthesis	11	0.297923
19	Protein homeostasis	6	0.855506
3	Carbohydrate metabolism	5	0.002388
2	Cellular respiration	5	0.185229
10	Redox homeostasis	5	0.210305
4	Amino acid metabolism	2	0.207697
21	Cell wall organisation	2	0.287358
18	Protein modification	2	0.575503
16	RNA processing	2	0.910829
6	Nucleotide metabolism	2	0.933061
27	Multi-process regulation	1	0.150744
26	External stimuli response	1	0.245505
22	Vesicle trafficking	1	0.442687
7	Co-enzyme metabolism	1	0.921600

Table S9: Mapman BINs representing upregulated and downregulated *X. schlechteri* proteins at late response to drying.

Bin	Name<br>**Upregulated proteins at LRD**	Elements (no.of proteins allocated)	p-value
35	Not assigned	48	3.66E-04
17	Protein biosynthesis	14	0.350356
7	Co-enzyme metabolism	5	0.701357
1	Photosynthesis	4	0.004989
18	Protein modification	4	0.067196
5	Lipid metabolism	4	0.776159
16	RNA processing	4	0.845021
10	Redox homeostasis	3	0.060389
19	Protein homeostasis	3	0.213074
26	External stimuli response	3	0.775051
9	Secondary metabolism	3	0.806496
23	Protein translocation	3	0.854240
2	Cellular respiration	2	0.156297
3	Carbohydrate metabolism	1	0.086411
13	Cell cycle organisation	1	0.506139
14	DNA damage response	1	0.506139
15	RNA biosynthesis	1	0.551926

Bin	Name<br>Downregulated proteins at LRD	Elements (no.of proteins allocated)	p-value
1	Photosynthesis	16	0.021967
35	Not assigned	14	0.335041
3	Carbohydrate metabolism	6	0.241955
19	Protein homeostasis	4	0.364960
10	Redox homeostasis	3	0.497135
21	Cell wall organisation	2	0.340023
7	Co-enzyme metabolism	2	0.896482
17	Protein biosynthesis	2	0.965407
27	Multi-process regulation	1	0.273913
26	External stimuli response	1	0.465759
4	Amino acid metabolism	1	0.584346
2	Cellular respiration	1	0.715337
22	Vesicle trafficking	1	0.761192

Table S10: List of the Bin names, id and description of the upregulated proteins at ERD

BinName	id	Description
**Secondary metabolism**. phenolics.flavonoid biosynthesis.flavonol glycosides.flavonol-3-O-rhamnosyltransferase'	xvis03_211917-pa	flavonol-3-O-rhamnosyltransferase (original description: augustus-gene-8.2-mRNA-1 protein Name:Similar to UGT78D2 UDP-glycosyltransferase 78D2 (Arabidopsis thaliana) AED:0.00 eAED:0.00 QI:173|1|1|1|1|1|2|210|454)'
**Secondary metabolism**. phenolics.flavonoid biosynthesis.aurones.aureusidin synthase'	xvis03_203578-pa	aureusidin synthase (original description: augustus-gene-30.42-mRNA-1 protein Name:Similar to Polyphenol oxidase, chloroplastic (Vitis vinifera) AED:0.01 eAED:0.01 QI:30|1|1|1|1|1|2|191|591)'

Table S11: List of the Bin names, id and description of the downregulated proteins at ERD

BinName	id	Description

**Coenzyme metabolism**.tetrapyrrol biosynthesis.uroporphyrinogen III formation.porphobilinogen deaminase'	xvis03_202672-pa	porphobilinogen deaminase (original description: processed-gene-54.103-mRNA-1 protein Name:Similar to HEMC Porphobilinogen deaminase, chloroplastic (Oryza sativa subsp. japonica) AED:0.01 eAED:0.01 QI:117|1|1|1|1|1|5|327|378)'
**Coenzyme metabolism.**tetrapyrrol biosynthesis.chlorophyll metabolism.Mg-protoporphyrin IX O-methyltransferase'	xvis03_200416-pa	Mg-protoporphyrin IX O-methyltransferase (original description: processed-gene-51.69-mRNA-1 protein Name:Similar to CHLM Magnesium protoporphyrin IX methyltransferase, chloroplastic (Arabidopsis thaliana) AED:0.34 eAED:0.34 QI:0|-1|0|1|-1|1|1|0|322)'
**Coenzyme metabolism**.tetrapyrrol biosynthesis.chlorophyll metabolism.protochlorophyllide oxidoreductase (pOR) activities.light-dependent pOR'	xvis03_212894-pa	light-dependent protochlorophyllide oxidoreductase (original description: augustus-gene- 2.1-mRNA-1 protein Name:Similar to PORB Protochlorophyllide reductase B, chloroplastic (Hordeum vulgare) AED:0.09 eAED:0.09 QI:162|1|1|1|1|1|5|457|393)'
**Coenzyme metabolism**.tetrapyrrol biosynthesis.chlorophyll metabolism.protochlorophyllide oxidoreductase (pOR) activities.light-dependent pOR'	xvis03_218927-pa	light-dependent protochlorophyllide oxidoreductase (original description: processed-gene-4.16-mRNA-1 protein Name:Similar to PORB Protochlorophyllide reductase B, chloroplastic (Hordeum vulgare) AED:0.12 eAED:0.12 QI:128|0.75|0.6|1|1|1|5|341|353)'

**Coenzyme metabolism**.tetrapyrrol biosynthesis.chlorophyll metabolism.chlorophyll(ide) interconversions.geranylgeranyl reductase (Chlp)'	xvis03_215774-pa	geranylgeranyl reductase (ChlP) (original description: augustus-gene-7.23-mRNA-1 protein Name:Similar to CHLP Geranylgeranyl diphosphate reductase, chloroplastic (Nicotiana tabacum) AED:0.00 eAED:0.00 QI:382|1|1|1|1|1|2|212|364)'
**protein homeostasis**.protein quality control.Hsp90 chaperone system.chaperone (Hsp90)'	xvis03_207665-pa	chaperone (Hsp90) (original description: processed-gene-12.71-mRNA-1 protein Name:Similar to HSP90 Endoplasmin homolog (Catharanthus roseus) AED:0.12 eAED:0.12 QI:42|1|1|1|1|1|15|358|812)'
**protein homeostasis**.protein quality control.Hsp90 chaperone system.chaperone (Hsp90)'	xvis03_224897-pa	chaperone (Hsp90) (original description: augustus-gene- 1.57-mRNA-1 protein Name:Similar to HSP90 Endoplasmin homolog (Catharanthus roseus) AED:0.10 eAED:0.10 QI:52|0.92|0.93|1|1|1|15|395|78 8)'
**protein homeostasis**.ubiquitin-proteasome system.26S proteasome.19S regulatory particle.non-ATPase components.regulatory component RPN8'	xvis03_212052-pa	regulatory component RPN8 of 26S proteasome (original description: augustus-gene-18.3-mRNA-1 protein Name:Similar to RPN8A 26S proteasome non-ATPase regulatory subunit 7 homolog A (Arabidopsis thaliana) AED:0.29 eAED:0.29 QI:0|0.9|0.81|0.90|1|1|11|444|3 63)'

**protein homeostasis**.proteolysis.cysteine-type peptidase activities.C13-class asparaginyl endopeptidase (Legumain)'	xvis03_211268-pa	asparaginyl endopeptidase (Legumain) (original description: processed-gene-9.25-mRNA-1 protein Name:Similar to Vacuolar-processing enzyme (Citrus sinensis) AED:0.07 eAED:0.07 QI:3|1|1|1|1|1|9|417|590)'
**Carbohydrate metabolism**.oxidative pentose phosphate pathway.oxidative phase.6-phosphogluconolactonase'	xvis03_213363-pa	6-phosphogluconolactonase (original description: processed-gene-12.66-mRNA-1 protein Name:Similar to Os09g0529100 Probable 6-phosphogluconolactonase 4, chloroplastic (Oryza sativa subsp. japonica) AED:0.04 eAED:0.04 QI:89|1|1|1|1|1|3|259|276)'
**Carbohydrate metabolism**.oxidative pentose phosphate pathway.non-oxidative phase.transaldolase'	xvis03_219185-pa	transaldolase (original description: processed-gene-8.51-mRNA-1 protein Name:Similar to tal Transaldolase (Acidothermus cellulolyticus (strain ATCC 43068 / 11B)) AED:0.03 eAED:0.03 QI:60|0.83|0.85|1|1|1|7|483|446)'
**photosynthesis.**photophosphorylation.cytochrome b6/f complex.Rieske iron-sulfur component petC'	xvis03_223095-pa	Rieske iron-sulfur component PetC of cytochrome b6/f complex (original description: augustus-gene-3.96-mRNA-1 protein Name:Similar to petC Cytochrome b6-f complex iron-sulfur subunit, chloroplastic (Oryza sativa subsp. japonica) AED:0.11 eAED:0.11 QI:167|0.33|0.75|1|1|1|4|287|178)'
**photosynthesis**.photophosphorylation.chlororespiration.NADH dehydrogenase-like (NDH) complex.lumen subcomplex L.component pnsL1'	xvis03_203608-pa	component PnsL1 of NDH lumen subcomplex L (original description: processed-gene-

		32.69-mRNA-1 protein Name:Similar to PNSL1 Photosynthetic NDH subunit of lumenal location 1, chloroplastic (Arabidopsis thaliana) AED:0.12 eAED:0.08 QI:81|1|0.85|1|1|1|7|565|263)'
**Nucleotide metabolism**.purines.phosphotransfers.adenylate kinase'	xvis03_207311-pa	adenylate kinase (original description: processed-gene-7.62-mRNA-1 protein Name:Similar to ADK-B Adenylate kinase 4 (Oryza sativa subsp. japonica) AED:0.05 eAED:0.05 QI:122|1|1|1|1|1|6|379|246)'
**Nucleotide metabolism**.purines.phosphotransfers.adenylate kinase'	xvis03_213605-pa	adenylate kinase (original description: processed-gene-14.84-mRNA-1 protein Name:Similar to Os08g0288200 Probable adenylate kinase 5, chloroplastic (Oryza sativa subsp. japonica) AED:0.22 eAED:0.22 QI:25|1|0.94|1|1|1|17|0|611)'

Table S12: List of the Bin names, id and description of the upregulated proteins at MRD

BinName	id	Description
**protein homeostasis**.protein quality control.cytosolic Hsp70 chaperone system.chaperone (Hsp70)'	xvis03_22222 3-pa	chaperone (Hsp70) (original description: processed-gene-2.73-mRNA-1 protein Name: Similar to HSP70 Heat shock cognate 70 kDa protein (Petunia hybrida) AED:0.11 eAED:0.11 QI:205|0.5|0.33|0.66|1|1|3|0|710)'

**protein homeostasis**.ubiquitin-proteasome system.ubiquitin-fold protein conjugation.RUB/NEDD8 conjugation (neddylation).ubiquitin-fold protein (RUB)'	xvis03_205813-pa	ubiquitin-fold protein (RUB) (original description: processed-gene-6.86-mRNA-1 protein Name: Similar to RUB2 Ubiquitin-NEDD8-like protein RUB2 (Oryza sativa subsp. japonica) AED:0.04 eAED:0.04 QI:73|1|1|1|1|1|3|301|153)'
**Carbohydrate metabolism.**sucrose metabolism.biosynthesis.cytosolic UDp-glucose pyrophosphorylase'	xvis03_202124-pa	cytosolic UDP-glucose pyrophosphorylase (original description: processed-gene-11.0-mRNA-1 protein Name:Similar to UGPA UTP--glucose-1-phosphate uridylyltransferase (Musa acuminata) AED:0.18 eAED:0.18 QI:271|0.88|0.89|1|1|1|19|372|458)'
**Carbohydrate metabolism**.starch metabolism.degradation.maltose metabolism.cytosolic alpha-glucan phosphorylase'	xvis03_204659-pa	cytosolic alpha-glucan phosphorylase (original description: augustus-gene-39.13-mRNA-1 protein Name:Similar to PHS2 Alpha-glucan phosphorylase 2, cytosolic (Arabidopsis thaliana) AED:0.23 eAED:0.23 QI:181|0.92|0.93|1|0.92|0.86|15|169|732)'
**Lipid metabolism**.glycerolipid biosynthesis.phosphatidylcholine.CTp:phosphorylcholine cytidylyltransferase'	xvis03_202655-pa	CTP:phosphorylcholine cytidylyltransferase (original description: processed-gene-53.13-mRNA-1 protein Name:Similar to CCT2 Choline-phosphate cytidylyltransferase 2 (Arabidopsis thaliana) AED:0.15 eAED:0.15 QI:50|1|1|1|1|1|8|392|291)'
**Lipid metabolism**.lipid degradation.phospholipase activities.phospholipase A2 activities.phospholipase A2 (ppLA2-II)'	xvis03_218200-pa	phospholipase A2 (pPLA2-II) (original description: augustus-gene-6.42-mRNA-1 protein Name:Similar to PLP1 Patatin-like protein 1 (Oryza sativa subsp. japonica) AED:0.26 eAED:0.26 QI:72|0.4|0.16|1|1|1|6|0|314)'

Table S13: List of the Bin names, id and description of the downregulated proteins at MRD

BinName	id	Description
**photosynthesis**.photophosphorylation.photosystem II.LHC-II complex.component LHCb1/2/3'	xvis03_211060-pa	component LHCb1/2/3 of LHC-II complex (original description: processed-gene-14.7-mRNA-1 protein Name:Similar to Chlorophyll a-b binding protein, chloroplastic (Spinacia oleracea) AED:0.01 eAED:0.01 QI:0|-1|0|1|-1|1|1|0|268)'
**photosynthesis**.photophosphorylation.photosystem II.LHC-II complex.component LHCb1/2/3'	xvis03_219722-pa	component LHCb1/2/3 of LHC-II complex (original description: augustus-gene-9.73-mRNA-1 protein Name:Similar to CAB36 Chlorophyll a-b binding protein 36, chloroplastic (Nicotiana tabacum) AED:0.36 eAED:0.36 QI:240|1|1|1|1|1|2|393|264)'
**photosynthesis**.photophosphorylation.photosystem II.LHC-II complex.component LHCb1/2/3'	xvis03_223077-pa	component LHCb1/2/3 of LHC-II complex (original description: processed-gene-1.89-mRNA-1 protein Name:Similar to CAB13 Chlorophyll a-b binding protein 13, chloroplastic (Solanum lycopersicum) AED:0.07 eAED:0.07 QI:182|1|1|1|1|1|4|188|263)'
**photosynthesis**.photophosphorylation.photosystem II.LHC-II complex.component LHCb1/2/3'	xvis03_224654-pa	component LHCb1/2/3 of LHC-II complex (original description: processed-gene-1.9-mRNA-1 protein Name:Similar to CAB21 Chlorophyll a-b binding protein 21, chloroplastic (Nicotiana tabacum) AED:0.11 eAED:0.11 QI:0|0|0|0.5|1|1|2|0|328)'
**photosynthesis**.photophosphorylation.photosystem II.LHC-II complex.component LHCb1/2/3'	xvis03_225066-pa	component LHCb1/2/3 of LHC-II complex (original description: processed-gene-0.82-mRNA-1 protein Name:Similar to Chlorophyll a-b binding protein,

		chloroplastic (Spinacia oleracea) AED:0.00 eAED:0.00 QI:0|-1|0|1|-1|1|1|0|268)'
**photosynthesis**.photophosphorylation.photosystem II.LHC-II complex.component LHCb4'	xvis03_212711-pa	component LHCb4 of LHC-II complex (original description: processed-gene-16.130-mRNA-1 protein Name:Similar to LHCB4.2 Chlorophyll a-b binding protein CP29.2, chloroplastic (Arabidopsis thaliana) AED:0.05 eAED:0.05 QI:1875|1|1|1|0.5|0.33|3|204|289)'
**photosynthesis**.photophosphorylation.photosystem II.LHC-II complex.component LHCb5'	xvis03_221799-pa	component LHCb5 of LHC-II complex (original description: processed-gene-5.86-mRNA-1 protein Name:Similar to LHCB5 Chlorophyll a-b binding protein CP26, chloroplastic (Arabidopsis thaliana) AED:0.06 eAED:0.06 QI:123|0.8|0.83|1|1|1|6|161|279)'
**photosynthesis**.photophosphorylation.photosystem II.LHC-II complex.component LHCb6'	xvis03_207968-pa	component LHCb6 of LHC-II complex (original description: augustus-gene-15.42-mRNA-1 protein Name:Similar to CAP10A Chlorophyll a-b binding protein CP24 10A, chloroplastic (Solanum lycopersicum) AED:0.00 eAED:0.00 QI:218|1|1|1|1|1|2|259|254)'
**photosynthesis.**photophosphorylation.photosystem II.pS-II complex.oxygen-evolving center (OEC) extrinsic proteins.component OEC33/psbO'	xvis03_208337-pa	component PsbO/OEC33 of PS-II oxygen-evolving center (original description: augustus-gene-6.16-mRNA-1 protein Name:Similar to PSBO Oxygen-evolving enhancer protein 1, chloroplastic (Solanum lycopersicum) AED:0.08 eAED:0.08 QI:229|1|1|1|0.5|0.33|3|86|332)'

**photosynthesis**.photophosphorylation.photosystem II.pS-II complex.oxygen-evolving center (OEC) extrinsic proteins.component OEC33/psbO'	xvis03_219859-pa	component PsbO/OEC33 of PS-II oxygen-evolving center (original description: augustus-gene-3.73-mRNA-1 protein Name:Similar to PSBO Oxygen-evolving enhancer protein 1, chloroplastic (Solanum lycopersicum) AED:0.03 eAED:0.03 QI:216|1|1|1|1|1|2|224|332)'
**photosynthesis.**photophosphorylation.photosystem II.pS-II complex.oxygen-evolving center (OEC) extrinsic proteins.Viridiplantae-specific components.component OEC23/psbp'	xvis03_209988-pa	component PsbP of PS-II oxygen-evolving center (original description: processed-gene-2.101-mRNA-1 protein Name:Similar to PSBP Oxygen-evolving enhancer protein 2, chloroplastic (Cucumis sativus) AED:0.32 eAED:0.32 QI:142|1|1|1|1|1|4|296|262)'
**photosynthesis.**photophosphorylation.photosystem II.assembly and maintenance.assembly factor (MET1)'	xvis03_221570-pa	MET1 protein involved in PS-II assembly (original description: augustus-gene-2.10-mRNA-1 protein Name:Similar to LSF1 Phosphoglucan phosphatase LSF1, chloroplastic (Arabidopsis thaliana) AED:0.06 eAED:0.06 QI:233|0.83|0.71|1|0.5|0.42|7|831|301)'
**photosynthesis**.photophosphorylation.cytochrome b6/f complex.Rieske iron-sulfur component petC'	xvis03_223095-pa	Rieske iron-sulfur component PetC of cytochrome b6/f complex (original description: augustus-

		gene-3.96-mRNA-1 protein Name:Similar to petC Cytochrome b6-f complex iron-sulfur subunit, chloroplastic (Oryza sativa subsp. japonica) AED:0.11 eAED:0.11 QI:167|0.33|0.75|1|1|1|4|287|178)'
**photosynthesis.** photophosphorylation. Cytb6/f to pS-I electron carriers.plastocyanin'	xvis03_217729-pa	plastocyanin electron carrier (original description: processed-gene-11.5-mRNA-1 protein Name:Similar to PETE Plastocyanin minor isoform, chloroplastic (Arabidopsis thaliana) AED:0.37 eAED:0.37 QI:0|0|0|0.16|1|1|6|0|366)'
**photosynthesis.**photophosphorylation.photosystem I.LHC-I complex.component LHCa1'	xvis03_225147-pa	component LHCa1 of LHC-I complex (original description: augustus-gene-0.41-mRNA-1 protein Name:Similar to CAB6A Chlorophyll a-b binding protein 6A, chloroplastic (Solanum lycopersicum) AED:0.00 eAED:0.00 QI:106|1|1|1|1|1|3|75|204)'
**photosynthesis**.photophosphorylation.photosystem I.LHC-I complex.component LHCa1'	xvis03_225829-pa	component LHCa1 of LHC-I complex (original description: augustus-gene-0.17-mRNA-1 protein Name:Similar to LHCA1 Chlorophyll a-b binding protein 6, chloroplastic (Arabidopsis thaliana) AED:0.45 eAED:0.45 QI:0|0.44|0.4|0.5|1|1|10|334|459)'
**photosynthesis.**photophosphorylation.photosystem I.pS-I complex.component psaH'	xvis03_205319-pa	component PsaH of PS-I complex (original description: processed-gene-30.54-mRNA-1 protein Name:Similar to PSAH1 Photosystem I reaction center subunit VI-1, chloroplastic (Arabidopsis thaliana) AED:0.09 eAED:0.09 QI:15|1|1|1|1|1|3|336|145)'

**photosynthesis.**photophosphorylation.linear electron flow.ferredoxin-NADp reductase (FNR) activity.ferredoxin-NADp oxidoreductase'	xvis03_207668-pa	ferredoxin-NADP oxidoreductase (original description: processed-gene-12.85-mRNA-1 protein Name:Similar to PETH Ferredoxin--NADP reductase, leaf isozyme, chloroplastic (Pisum sativum) AED:0.14 eAED:0.14 QI:231|1|1|1|1|1|9|392|366)'
**photosynthesis.**photophosphorylation.chlororespiration.NADH dehydrogenase-like (NDH) complex.lumen subcomplex L.component pnsL1'	xvis03_203608-pa	component PnsL1 of NDH lumen subcomplex L (original description: processed-gene-32.69-mRNA-1 protein Name:Similar to PNSL1 Photosynthetic NDH subunit of lumenal location 1, chloroplastic (Arabidopsis thaliana)

		AED:0.12 eAED:0.08 QI:81|1|0.85|1|1|1|7|565|263)'
**photosynthesis.**calvin cycle.ribulose-1,5-bisphosphat carboxylase/oxygenase (RuBisCo) activity.RuBisCo heterodimer.small subunit'	xvis03_223144-pa	small subunit of ribulose-1,5-bisphosphat carboxylase/oxygenase heterodimer (original description: processed-gene-3.56-mRNA-1 protein Name:Similar to RBCS1 Ribulose bisphosphate carboxylase small chain, chloroplastic (Musa acuminata) AED:0.05 eAED:0.05 QI:145|1|1|1|1|1|3|356|174)'

**photosynthesis**.calvin cycle.ribulose-1,5-bisphosphat carboxylase/oxygenase (RuBisCo) activity.RuBisCo assembly.auxiliary co-chaperone (CpN20)'	xvis03_206991-pa	CPN20 auxiliary co-chaperone involved in RuBisCo assembly (original description: augustus-gene-10.52-mRNA-1 protein Name:Similar to CPN21 20 kDa chaperonin, chloroplastic (Arabidopsis thaliana) AED:0.07 eAED:0.07 QI:99|1|1|1|1|1|5|439|254)'
**photosynthesis.**calvin cycle.ribulose-1,5-bisphosphat carboxylase/oxygenase (RuBisCo) activity.RuBisCo assembly.auxiliary co-chaperone (CpN20)'	xvis03_217041-pa	CPN20 auxiliary co-chaperone involved in RuBisCo assembly (original description: augustus-gene-12.20-mRNA-1 protein Name:Similar to CPN21 20 kDa chaperonin, chloroplastic (Arabidopsis thaliana) AED:0.12 eAED:0.12 QI:32|0.33|0.71|0.71|1|1|7|174|298)'
**photosynthesis**.calvin cycle.ribulose-1,5-bisphosphat carboxylase/oxygenase (RuBisCo) activity.RuBisCo regulation.ATp-dependent activase (RCA)'	xvis03_223853-pa	ATP-dependent activase involved in RuBisCo regulation (original description: processed-gene-1.15-mRNA-1 protein Name:Similar to RCA2 Ribulose bisphosphate carboxylase/oxygenase activase 2, chloroplastic (Larrea tridentata) AED:0.12 eAED:0.12 QI:111|1|1|1|0.83|0.71|7|332|438)'
**photosynthesis.**calvin cycle.triosephosphate isomerase'	xvis03_224225-pa	triosephosphate isomerase (original description: augustus-gene-0.55-mRNA-1 protein Name:Similar to TPIP1 Triosephosphate isomerase, chloroplastic (Spinacia oleracea) AED:0.32 eAED:0.32 QI:0|0|0|0.55|0.87|1|9|0|311)'
**photosynthesis**.calvin cycle.transketolase'	xvis03_207774-pa	transketolase (original description: processed-gene-21.50-mRNA-1 protein Name:Similar to Transketolase, chloroplastic (Solanum tuberosum) AED:0.17 eAED:0.17 QI:154|0.85|0.75|1|1|1|8|0|716)'

**photosynthesis.**calvin cycle.transketolase'	xvis03_219001-pa	transketolase (original description: processed-gene-2.79-mRNA-1 protein Name:Similar to Transketolase, chloroplastic (Solanum tuberosum) AED:0.04 eAED:0.04 QI:238|1|1|1|1|1|7|473|742)'
**photosynthesis.**photorespiration.serine hydroxymethyltransferase (SHM)'	xvis03_208040-pa	serine hydroxymethyltransferase (original description: processed-gene-21.10-mRNA-1 protein Name:Similar to SHM4 Serine hydroxymethyltransferase 4 (Arabidopsis thaliana) AED:0.05 eAED:0.05 QI:76|1|1|1|1|1|4|235|515)'
**protein biosynthesis**.ribosome biogenesis.large ribosomal subunit (LSU).LSU proteome.component RpL3'	xvis03_216531-pa	component RPL3 of LSU proteome component (original description: augustus-gene-9.118-mRNA-1 protein Name:Similar to RPL3 60S ribosomal protein L3 (Oryza sativa subsp. japonica) AED:0.07 eAED:0.07 QI:0|1|0.83|1|1|0.83|6|237|407)'
**protein biosynthesis**.ribosome biogenesis.large ribosomal subunit (LSU).LSU proteome.component RpL3'	xvis03_220863-pa	component RPL3 of LSU proteome component (original description: processed-gene-0.82-mRNA-1 protein Name:Similar to RPL3 60S ribosomal protein L3 (Oryza sativa subsp. japonica) AED:0.08 eAED:0.08 QI:54|1|1|1|1|1|6|258|389)'

**protein biosynthesis.**ribosome biogenesis.large ribosomal subunit (LSU).LSU proteome.component RpL8'	xvis03_215883-pa	component RPL8 of LSU proteome component (original description: processed-gene-4.2-mRNA-1 protein Name:Similar to RPL8 60S ribosomal protein L8 (Solanum lycopersicum) AED:0.00 eAED:0.00 QI:71|1|1|1|1|1|2|167|261)'
**protein biosynthesis**.ribosome biogenesis.small ribosomal subunit (SSU).SSU proteome.component RpS2'	xvis03_220973-pa	component RPS2 of SSU proteome (original description: augustus-gene-5.51-mRNA-1 protein Name:Similar to RPS2C 40S ribosomal protein S2-3 (Arabidopsis thaliana) AED:0.29 eAED:0.29 QI:54|1|1|1|0.5|0.33|3|154|274)'
**protein biosynthesis.**ribosome biogenesis.small ribosomal subunit (SSU).SSU proteome.component RpS17'	xvis03_204705-pa	component RPS17 of SSU proteome (original description: processed-gene-11.1-mRNA-1 protein Name:Similar to RPS17 40S ribosomal protein S17 (Solanum lycopersicum) AED:0.06 eAED:0.06 QI:0|-1|0|1|-1|1|1|0|143)'
**protein biosynthesis**.ribosome biogenesis.small ribosomal subunit (SSU).SSU proteome.component RpS17'	xvis03_207825-pa	component RPS17 of SSU proteome (original description: processed-gene-26.31-mRNA-1 protein Name:Similar to RPS17 40S ribosomal protein S17 (Solanum lycopersicum) AED:0.03 eAED:0.03 QI:0|-1|0|1|-

		1|1|1|0|146)'
**protein biosynthesis**.organelle machinery.plastidial ribosome.large ribosomal subunit proteome.component psRpL4'	xvis03_213652-pa	component psRPL4 of large ribosomal subunit proteome (original description: processed-gene-16.266-mRNA-1 protein Name:Similar to RPL4 50S ribosomal protein L4, chloroplastic (Nicotiana tabacum) AED:0.18 eAED:0.18 QI:0|-1|0|1|-1|1|1|0|272)'
**protein biosynthesis**.organelle machinery.plastidial ribosome.large ribosomal subunit proteome.component psRpL5'	xvis03_220112-pa	component psRPL5 of large ribosomal subunit proteome (original description: augustus-gene-8.33-mRNA-1 protein Name:Similar to RPL5 50S ribosomal protein L5, chloroplastic (Arabidopsis thaliana) AED:0.02 eAED:0.03 QI:29|0.5|0.33|1|1|1|3|0|284)'
**protein biosynthesis.**organelle machinery.plastidial ribosome.large ribosomal subunit proteome.component psRpL9'	xvis03_202061-pa	component psRPL9 of large ribosomal subunit proteome (original description: processed-gene-6.69-mRNA-1 protein Name:Similar to RPL9 50S ribosomal protein L9, chloroplastic (Arabidopsis thaliana) AED:0.06 eAED:0.06 QI:100|1|1|1|0.83|0.71|7|568|196)'
**protein biosynthesis.**organelle machinery.plastidial ribosome.small ribosomal subunit proteome.component psRpS5'	xvis03_207337-pa	component psRPS5 of small ribosomal subunit proteome (original description: processed-gene-10.83-mRNA-1 protein Name:Similar to rps5 30S ribosomal protein S5, chloroplastic (Arabidopsis thaliana) AED:0.00 eAED:0.00 QI:186|1|1|1|1|1|2|216|309)'

**protein biosynthesis**.organelle machinery.translation initiation.initiation factor (IF-3)'	xvis03_224254-pa	IF-3 translation initiation factor (original description: augustus-gene-0.0-mRNA-1 protein Name:Similar to infC Translation initiation factor IF-3 (Nostoc sp. (strain PCC 7120 / UTEX 2576)) AED:0.11 eAED:0.11 QI:31|1|1|1|0.55|0.5|10|360|276)'

**protein homeostasis**.protein quality control.ER Quality Control (ERQC) machinery.calnexin/calreticulin chaperone system.CNX-CRT cycle.lectin chaperone (CNX)'	xvis03_219178-pa	lectin chaperone (CNX) (original description: processed-gene-7.19-mRNA-1 protein Name:Similar to CNX1 Calnexin homolog 1 (Arabidopsis thaliana) AED:0.09 eAED:0.09 QI:144|1|1|1|0.83|0.71|7|378|540)'
**protein homeostasis.**protein quality control.Hsp60 chaperone system.Hsp60-co-chaperone (Hsp20)'	xvis03_206991-pa	Hsp60-co-chaperone (Hsp20) (original description: augustus-gene-10.52-mRNA-1 protein Name:Similar to CPN21 20 kDa chaperonin, chloroplastic (Arabidopsis thaliana) AED:0.07 eAED:0.07 QI:99|1|1|1|1|1|5|439|254)'
**protein homeostasis.**protein quality control.Hsp60 chaperone system.Hsp60-co-chaperone (Hsp20)'	xvis03_217041-pa	Hsp60-co-chaperone (Hsp20) (original description: augustus-gene-12.20-mRNA-1 protein Name:Similar to CPN21 20 kDa chaperonin, chloroplastic (Arabidopsis thaliana) AED:0.12 eAED:0.12 QI:32|0.33|0.71|0.71|1|1|7|174|298)'

**protein homeostasis**.ubiquitin-proteasome system.26S proteasome.20S core particle.alpha-type components.component alpha type-2'	xvis03_213988-pa	component alpha type-2 of 26S proteasome (original description: augustus-gene-11.18-mRNA-1 protein Name:Similar to PAB1 Proteasome subunit alpha type-2 (Oryza sativa subsp. japonica) AED:0.05 eAED:0.05 QI:70|1|1|1|1|1|10|615|222)'
**protein homeostasis.**ubiquitin-proteasome system.26S proteasome.20S core particle.beta-type components.component beta type-7'	xvis03_202356-pa	component beta type-7 of 26S proteasome (original description: processed-gene-29.12-mRNA-1 protein Name:Similar to PBG1 Proteasome subunit beta type-4 (Arabidopsis thaliana) AED:0.13 eAED:0.13 QI:0|0.71|0.75|0.87|1|1|8|335|329)'
**protein homeostasis.**proteolysis.cysteine-type peptidase activities.C13-class asparaginyl endopeptidase (Legumain)'	xvis03_211268-pa	asparaginyl endopeptidase (Legumain) (original description: processed-gene-9.25-mRNA-1 protein Name:Similar to Vacuolar-processing enzyme (Citrus sinensis) AED:0.07 eAED:0.07 QI:3|1|1|1|1|1|9|417|590)'
**Carbohydrate metabolism.**sucrose metabolism.biosynthesis.cytosolic triose-phosphate isomerase'	xvis03_211745-pa	cytosolic triose-phosphate isomerase (original description: augustus-gene-13.21-mRNA-1 protein Name:Similar to TPIP1 Triosephosphate isomerase, cytosolic (Petunia hybrida) AED:0.34 eAED:0.34 QI:46|0.88|0.8|0.9|1|1|10|0|326)'

**Carbohydrate metabolism.**starch metabolism.biosynthesis.ADp-glucose pyrophosphorylase'	xvis03_220998-pa	ADP-glucose pyrophosphorylase (original description: augustus-gene-7.121-mRNA-1 protein Name:Similar to ADG2 Glucose-1-phosphate adenylyltransferase large subunit 1, chloroplastic (Arabidopsis thaliana) AED:0.11 eAED:0.11 QI:196|0.92|0.86|1|0.85|0.86|15|136|540)'
**Carbohydrate metabolism**.oxidative pentose phosphate pathway.non-oxidative phase.transketolase'	xvis03_207774-pa	transketolase (original description: processed-gene-21.50-mRNA-1 protein Name:Similar to Transketolase, chloroplastic (Solanum tuberosum) AED:0.17 eAED:0.17 QI:154|0.85|0.75|1|1|1|8|0|716)'
**Carbohydrate metabolism.**oxidative pentose phosphate pathway.non-oxidative phase.transketolase'	xvis03_219001-pa	transketolase (original description: processed-gene-2.79-mRNA-1 protein Name:Similar to Transketolase, chloroplastic (Solanum tuberosum) AED:0.04 eAED:0.04 QI:238|1|1|1|1|1|7|473|742)'
**Carbohydrate metabolism**.plastidial glycolysis.triosephosphate isomerase'	xvis03_224225-pa	triosephosphate isomerase (original description: augustus-gene-0.55-mRNA-1 protein Name:Similar to TPIP1 Triosephosphate isomerase, chloroplastic (Spinacia oleracea) AED:0.32 eAED:0.32 QI:0|0|0|0.55|0.87|1|9|0|311)'
**Cellular respiration**.glycolysis.cytosolic glycolysis.triosephosphate isomerase'	xvis03_211745-pa	triosephosphate isomerase (original description: augustus-gene-13.21-mRNA-1 protein Name:Similar to TPIP1 Triosephosphate isomerase, cytosolic (Petunia hybrida) AED:0.34 eAED:0.34 QI:46|0.88|0.8|0.9|1|1|10|0|326)'

**Cellular respiration.**glycolysis.methylglyoxal degradation.lactoyl-glutathione lyase (GLX1)'	xvis03_210597-pa	lactoyl-glutathione lyase (GLX1) (original description: augustus-gene-8.18-mRNA-1 protein Name:Similar to At1g67280 Probable lactoylglutathione lyase, chloroplast (Arabidopsis thaliana) AED:0.06 eAED:0.06 QI:224|1|1|1|1|1|9|419|372)'
**Cellular respiration.**tricarboxylic acid cycle.mitochondrial NAD-dependent malic enzyme'	xvis03_201588-pa	mitochondrial NAD-dependent malic enzyme (original description: augustus-gene-28.7-mRNA-1 protein Name:Similar to NAD-ME1 NAD-dependent malic enzyme 1, mitochondrial (Arabidopsis thaliana) AED:0.22 eAED:0.22 QI:0|0.77|0.78|0.86|0.77|0.82|23|290|790)'
**Cellular respiration.**oxidative phosphorylation.cytochrome c reductase complex.component cytochrome c1'	xvis03_206281-pa	cytochrome c1 component of cytochrome c reductase complex (original description: augustus-gene-15.74-mRNA-1 protein Name:Similar to CYCL Cytochrome c1-1, heme protein, mitochondrial (Solanum tuberosum) AED:0.28 eAED:0.28 QI:33|0.85|0.87|1|1|1|8|457|361)'

**Cellular respiration.**oxidative phosphorylation.ATp synthase complex.peripheral MF1 subcomplex.subunit delta_'	xvis03_218722-pa	subunit delta_ of ATP synthase peripheral MF1 subcomplex (original description: augustus-gene-3.37-mRNA-1 protein Name:Similar to ATP synthase subunit delta', mitochondrial (Ipomoea batatas) AED:0.20 eAED:0.20 QI:67|0|0.25|0.5|0.33|0.75|4|0|208)'
**Redox homeostasis**.enzymatic reactive oxygen species scavengers.catalase'	xvis03_220733-pa	catalase (original description: processed-gene-1.13-mRNA-1 protein Name:Similar to CAT1 Catalase-1 (Triticum aestivum) AED:0.19 eAED:0.19 QI:0|0|0|0.66|1|1|9|0|623)'
**Redox homeostasis**.hydrogen peroxide removal.ascorbate-glutathione cycle.ascorbate peroxidase (ApX)'	xvis03_201883-pa	ascorbate peroxidase (APX) (original description: augustus-gene-50.73-mRNA-1 protein Name:Similar to APX1 L-ascorbate peroxidase, cytosolic (Pisum sativum) AED:0.15 eAED:0.15 QI:0|0.85|0.75|1|1|0.87|8|414|307)'
**Redox homeostasis.**hydrogen peroxide removal.ascorbate-glutathione cycle.ascorbate peroxidase (ApX)'	xvis03_214214-pa	ascorbate peroxidase (APX) (original description: processed-gene-11.33-mRNA-1 protein Name:Similar to APX1 L-ascorbate peroxidase, cytosolic (Pisum sativum) AED:0.17 eAED:0.17 QI:131|1|1|1|1|1|7|558|249)'
**Redox homeostasis**.hydrogen peroxide removal.ascorbate-glutathione cycle.ascorbate peroxidase (ApX)'	xvis03_222105-pa	ascorbate peroxidase (APX) (original description: augustus-gene-5.53-mRNA-1 protein Name:Similar to APXT L-ascorbate peroxidase T, chloroplastic (Arabidopsis thaliana) AED:0.32 eAED:0.32 QI:0|0.5|0.45|0.72|0.9|0.81|11|0|364)'

**Redox homeostasis.**hydrogen peroxide removal.ascorbate-glutathione cycle.ascorbate peroxidase (ApX)'	xvis03_223438-pa	ascorbate peroxidase (APX) (original description: augustus-gene-1.2-mRNA-1 protein Name:Similar to APX4 Probable L-ascorbate peroxidase 4 (Oryza sativa subsp. japonica) AED:0.10 eAED:0.10 QI:188|1|1|1|1|1|9|226|284)'
**Amino acid metabolism.**biosynthesis.aspartate family.aspartate-derived amino acids.methionine.salvage pathway.bifunctional methylthioribulose-1-phosphate dehydratase and enolase-phosphatase'	xvis03_223588-pa	bifunctional methylthioribulose-1-phosphate dehydratase and enolase-phosphatase (original description: processed-gene-1.56-mRNA-1 protein Name:Similar to VIT_19s0014g02480 Probable bifunctional methylthioribulose-1-phosphate dehydratase/enolase-phosphatase E1 1 (Vitis vinifera) AED:0.24 eAED:0.24 QI:0|0.9|0.90|0.90|1|1|11|237|411)'
**Amino acid metabolism**.biosynthesis.serine family.glycine.serine hydroxymethyltransferase	xvis03_208040-pa	serine hydroxymethyltransferase (original description: processed-gene-21.10-mRNA-1 protein Name:Similar to SHM4 Serine hydroxymethyltransferase 4 (Arabidopsis thaliana) AED:0.05 eAED:0.05 QI:76|1|1|1|1|1|4|235|515)'
**Nucleotide metabolism.**purines.phosphotransfers.adenylate kinase'	xvis03_207311-pa	adenylate kinase (original description: processed-gene-7.62-mRNA-1 protein Name:Similar to ADK-B Adenylate kinase 4 (Oryza sativa subsp. japonica) AED:0.05 eAED:0.05 QI:122|1|1|1|1|1|6|379|246)'

**Nucleotide metabolism**.purines.phosphotransfers.adenylate kinase'	xvis03_213605-pa	adenylate kinase (original description: processed-gene-14.84-mRNA-1 protein Name:Similar to Os08g0288200 Probable adenylate kinase 5, chloroplastic (Oryza sativa subsp. japonica) AED:0.22 eAED:0.22 QI:25|1|0.94|1|1|1|17|0|611)'
**RNA processing.**pre-mRNA splicing. U2-type-intron-specific major spliceosome. U2 small nuclear ribonucleoprotein particle (snRNp).protein factor (U2A)'	xvis03_205605-pa	protein factor U2A of U2 small nuclear ribonucleoprotein particle (snRNP) (original description: processed-gene-17.119-mRNA-1 protein Name:Similar to At1g09760 U2 small nuclear ribonucleoprotein A' (Arabidopsis thaliana) AED:0.23 eAED:0.27 QI:0|0.83|0.71|1|1|1|7|448|347)'
**RNA processing**.organelle machinery.ribonuclease activities.endoribonuclease (CSp41)'	xvis03_213217-pa	endoribonuclease (CSP41) (original description: augustus-gene-16.1-mRNA-1 protein Name:Similar to CSP41A Chloroplast stem-loop binding protein of 41 kDa a, chloroplastic (Arabidopsis thaliana) AED:0.01 eAED:0.01
**protein modification**.protein folding.protein folding catalyst (FKBp)'	xvis03_208652-pa	protein folding catalyst (FKBP) (original description: processed-gene-19.48-mRNA-1 protein Name:Similar to FKBP62 Peptidyl-prolyl cis-trans isomerase FKBP62 (Arabidopsis thaliana) AED:0.12 eAED:0.12 QI:105|0.91|0.92|1|1|1|13|323|570)'

**protein modification**.protein folding.protein folding catalyst (FKBp)'	xvis03_224849-pa	protein folding catalyst (FKBP) (original description: processed-gene-1.76-mRNA-1 protein Name:Similar to FKBP19 Peptidyl-prolyl cis-trans isomerase FKBP19, chloroplastic (Arabidopsis thaliana) AED:0.46 eAED:0.49 QI:0|0.72|0.83|0.91|0.90|0.83|12|203|320)'
**Cell wall organisation**.pectin.rhamnogalacturonan I.modification and degradation.beta-galactosidase (BGAL)'	xvis03_208987-pa	beta-galactosidase (BGAL) (original description: augustus- gene-4.4-mRNA-1 protein Name:Similar to BGAL9 Beta- galactosidase 9 (Arabidopsis thaliana) AED:0.06 eAED:0.06 QI:63|1|1|1|0.94|0.94|19|309|904)'
**Cell wall organisation.**pectin.rhamnogalacturonan I.modification and degradation.beta-galactosidase (BGAL)'	xvis03_221434-pa	beta-galactosidase (BGAL) (original description: processed- gene-2.106-mRNA-1 protein Name:Similar to Beta- galactosidase (Asparagus officinalis) AED:0.13 eAED:0.13 QI:277|1|1|1|1|1|19|418|832)'

Table S14: List of the Bin names, id and description of the upregulated proteins at LRD

**BinName**	**id**	**Description**
**protein biosynthesis.**ribosome biogenesis.large ribosomal subunit (LSU).LSU proteome.component RpL23'	xvis03_202929-pa	component RPL23 of LSU proteome component (original description: augustus-gene-26.5-mRNA-1 protein Name:Similar to RPL23A 60S ribosomal protein L23 (Arabidopsis thaliana) AED:0.39 eAED:0.40 QI:0|0|0|0.5|1|1|6|0|279)'
**protein biosynthesis**.ribosome biogenesis.small ribosomal subunit (SSU).SSU proteome.component RpS12'	xvis03_210337-pa	component RPS12 of SSU proteome (original description: augustus-gene-8.1-mRNA-1 protein Name:Similar to RPS12 40S ribosomal protein S12 (Hordeum vulgare) AED:0.38 eAED:0.38 QI:0|1|0.75|1|1|1|4|393|161)'

**protein biosynthesis.**ribosome biogenesis.small ribosomal subunit (SSU).SSU proteome.component RpS12'	xvis03_221354-pa	component RPS12 of SSU proteome (original description: augustus-gene-2.78-mRNA-1 protein Name:Similar to RPS12 40S ribosomal protein S12 (Hordeum vulgare) AED:0.26 eAED:0.26 QI:0|1|0.75|1|1|1|4|274|184)'
**protein biosynthesis.**ribosome biogenesis.small ribosomal subunit (SSU).SSU proteome.component RpS27'	xvis03_205594-pa	component RPS27 of SSU proteome (original description: augustus-gene-17.68-mRNA-1 protein Name:Similar to RPS27B 40S ribosomal protein S27-2 (Arabidopsis thaliana) AED:0.09 eAED:0.09 QI:0|1|0.75|1|1|1|4|342|163)'
**protein biosynthesis.**ribosome biogenesis.small ribosomal subunit (SSU).SSU proteome.component RpS27'	xvis03_209876-pa	component RPS27 of SSU proteome (original description: augustus-gene-14.5-mRNA-1 protein Name:Similar to RPS27B 40S ribosomal protein S27-2 (Arabidopsis thaliana) AED:0.17 eAED:0.17 QI:86|1|1|1|0.66|0.75|4|47|86)'
**protein biosynthesis.**mRNA quality control.NMD Nonsense-Mediated Decay.eRF1-eRF3 aberrant mRNA detection complex.component eRF1'	xvis03_209643-pa	eRF1 peptide release factor (original description: processed-gene-16.29-mRNA-1 protein Name:Similar to ERF1-3 Eukaryotic peptide chain release factor subunit 1-3 (Arabidopsis thaliana) AED:0.00 eAED:0.00 QI:0|-1|0|1|-1|1|1|0|437)'
**protein biosynthesis.**translation initiation.pre-Initiation Complex (pIC) module.eIF3 mRNA-to-pIC binding complex.component eIF3g'	xvis03_223860-pa	component eIF3g of eIF3 mRNA-to-PIC binding complex (original description: processed-gene-2.57-mRNA-1 protein Name:Similar to v1g171563 Eukaryotic translation initiation factor 3 subunit G (Nematostella vectensis) AED:0.02

		eAED:0.02 QI:0|1|0.5|1|1|1|2|3401|289)'
**protein biosynthesis.**translation initiation.mRNA loading.mRNA poly-A-tail binding factor (pABp)'	xvis03_217585-pa	mRNA poly-A-tail binding factor (PABP) (original description: augustus-gene-6.44-mRNA-1 protein Name:Similar to PAB8 Polyadenylate-binding protein 8 (Arabidopsis thaliana) AED:0.09 eAED:0.09 QI:312|0.75|0.66|1|1|1|9|460|634)'
**protein biosynthesis**.translation elongation.eEF1 aminoacyl-tRNA binding factor activity.eEF1B eEF1A-GDp-recycling complex.component eEF1B-beta/-delta'	xvis03_202784-pa	component eEF1B-beta/-delta of eEF1B eEF1A-GDP-recycling complex (original description: processed-gene-9.6-mRNA-1 protein Name:Similar to At5g12110 Elongation factor 1-beta 1 (Arabidopsis thaliana) AED:0.07 eAED:0.07 QI:28|1|1|1|1|1|6|211|226)'
**protein biosynthesis.**translation elongation.eEF5 poly-p/G elongation factor activity.poly-p/G elongation factor (eEF5/eIF5A)'	xvis03_208182-pa	poly-P/G elongation factor (eEF5/eIF5A) (original description: processed-gene-16.15-mRNA-1 protein Name:Similar to EIF-5A2 Eukaryotic translation initiation factor 5A-2 (Nicotiana plumbaginifolia) AED:0.11 eAED:0.11 QI:80|1|1|1|0.8|0.66|6|225|159)'
**protein biosynthesis.**translation elongation.eEF5 poly-p/G elongation factor activity.poly-p/G elongation factor (eEF5/eIF5A)'	xvis03_214372-pa	poly-P/G elongation factor (eEF5/eIF5A) (original description: processed-gene-6.62-mRNA-1 protein Name:Similar to Eukaryotic translation initiation factor 5A (Manihot esculenta) AED:0.20 eAED:0.20 QI:188|1|1|1|0.8|0.66|6|353|159)'

**protein biosynthesis.**translation elongation.eEF5 poly-p/G elongation factor activity.poly-p/G elongation factor (eEF5/eIF5A)'	xvis03_221720-pa	poly-P/G elongation factor (eEF5/eIF5A) (original description: augustus-gene-5.43-mRNA-1 protein Name:Similar to EIF-5A2 Eukaryotic translation initiation factor 5A-2 (Nicotiana plumbaginifolia) AED:0.05 eAED:0.05 QI:177|1|1|1|0.8|0.66|6|330|160)'
**protein biosynthesis**.organelle machinery.plastidial ribosome.large ribosomal subunit proteome.component psRpL18'	xvis03_224660-pa	component psRPL18 of large ribosomal subunit proteome (original description: processed-gene-1.62-mRNA-1 protein Name:Similar to FEN1 Flap endonuclease 1 (Zea mays) AED:0.25 eAED:0.26 QI:20|0.7|0.71|0.95|1|1|21|362|621)'
**protein biosynthesis**.organelle machinery.translation elongation.elongation factor (EF-Tu)'	xvis03_212520-pa	EF-Tu translation elongation factor (original description: augustus-gene-6.14-mRNA-1 protein Name:Similar to TUFB1 Elongation factor Tu, chloroplastic (Glycine max) AED:0.04 eAED:0.16 QI:0|0|0.5|1|1|1|2|351|455)'
**Coenzyme metabolism.**iron-sulfur cluster assembly machinery.plastidial SUF system.assembly phase.SUF-SE sulfur transfer complex.cysteine desulfurase component NFS2'	xvis03_220549-pa	cysteine desulfurase component NFS2 of plastidial SUF system assembly phase (original description: processed-gene-6.81-mRNA-1 protein Name:Similar to NFS2 Cysteine desulfurase 1, chloroplastic (Arabidopsis thaliana) AED:0.13 eAED:0.23 QI:55|0.77|0.8|1|1|1|10|0|498)'
**Coenzyme metabolism**.iron-sulfur cluster assembly machinery.mitochondrial ISC system.transfer phase.assembly factor (IBA57)'	xvis03_224887-pa	assembly factor BA57 of mitochondrial ISC system transfer phase (original description: augustus-gene-0.36-mRNA-1 protein Name:Similar to At4g12130 Putative transferase At4g12130,

		mitochondrial (Arabidopsis thaliana) AED:0.05 eAED:0.05 QI:50|1|1|1|1|1|5|427|411)'
**Coenzyme metabolism**.tetrapyrrol biosynthesis.5-aminolevulinic acid formation.glutamate-1-semialdehyde-2,1-aminomutase'	xvis03_216135-pa	glutamate-1-semialdehyde-2,1-aminomutase (original description: augustus-gene-2.5-mRNA-1 protein Name:Similar to GSA Glutamate-1-semialdehyde 2,1-aminomutase, chloroplastic (Oryza sativa subsp. japonica) AED:0.00 eAED:0.00 QI:90|1|1|1|1|1|3|296|478)'
**Coenzyme metabolism**.tetrapyrrol biosynthesis.chlorophyll metabolism.magnesium-chelatase complex.component CHL-I'	xvis03_222911-pa	component CHL-I of magnesium-chelatase complex (original description: processed-gene-1.35-mRNA-1 protein Name:Similar to CHLI Magnesium-chelatase subunit ChlI, chloroplastic (Oryza sativa subsp. japonica) AED:0.00 eAED:0.00 QI:218|1|1|1|1|1|3|269|418)'
**Coenzyme metabolism**.tetrapyrrol biosynthesis.chlorophyll metabolism.protochlorophyllide oxidoreductase (pOR) activities.light-dependent pOR'	xvis03_212894-pa	light-dependent protochlorophyllide oxidoreductase (original description: augustus-gene-2.1-mRNA-1 protein Name:Similar to PORB Protochlorophyllide reductase B, chloroplastic (Hordeum vulgare) AED:0.09 eAED:0.09 QI:162|1|1|1|1|1|5|457|393)'
**photosynthesis**.photophosphorylation.chlororespiration. NADH dehydrogenase-like (NDH) complex.assembly and stabilization.Cpn60 chaperonin heterodimer.subunit beta'	xvis03_205691-pa	subunit beta of Cpn60 chaperonin complex (original description: processed-gene-31.124-mRNA-1 protein Name:Similar to RuBisCO large subunit-binding protein subunit beta, chloroplastic (Pisum sativum) AED:0.20 eAED:0.20 QI:97|0.8|0.81|0.87|0.93|0.87|16|0|678)'

**photosynthesis.**calvin cycle.ribulose-1,5-bisphosphat carboxylase/oxygenase (RuBisCo) activity.RuBisCo heterodimer.small subunit'	xvis03_223144-pa	small subunit of ribulose-1,5-bisphosphat carboxylase/oxygenase heterodimer (original description: processed-gene-3.56-mRNA-1 protein Name:Similar to RBCS1 Ribulose bisphosphate carboxylase small chain, chloroplastic (Musa acuminata) AED:0.05 eAED:0.05 QI:145|1|1|1|1|1|3|356|174)'
**photosynthesis**.calvin cycle.ribulose-1,5-bisphosphat carboxylase/oxygenase (RuBisCo) activity.RuBisCo assembly.CpN60 assembly chaperone complex.component CpN60b'	xvis03_205691-pa	component CPN60b of CPN60 assembly chaperone complex (original description: processed-gene-31.124-mRNA-1 protein Name:Similar to RuBisCO large subunit-binding protein subunit beta, chloroplastic (Pisum sativum) AED:0.20 eAED:0.20 QI:97|0.8|0.81|0.87|0.93|0.87|16|0|678)'
**photosynthesis**.calvin cycle.phosphoglycerate kinase'	xvis03_201416-pa	phosphoglycerate kinase (original description: processed-gene-12.20-mRNA-1 protein Name:Similar to Phosphoglycerate kinase, chloroplastic (Nicotiana tabacum) AED:0.04 eAED:0.04 QI:190|1|1|1|1|1|6|289|477)'
**photosynthesis.**photorespiration.glycine decarboxylase complex.lipoamide-containing component H-protein'	xvis03_220076-pa	lipoamide-containing component H-protein of glycine cleavage system (original description: augustus-gene-4.88-mRNA-1 protein Name:Similar to GDCSH Glycine cleavage system H protein, mitochondrial (Oryza sativa subsp. japonica) AED:0.04 eAED:0.04 QI:127|1|1|1|1|1|4|484|165)'
**Cellular respiration**.glycolysis.cytosolic glycolysis. Phosphoglycerate kinase'	xvis03_201416-pa	phosphoglycerate kinase (original description: processed-gene-12.20-mRNA-1 protein Name:Similar to Phosphoglycerate kinase, chloroplastic (Nicotiana tabacum)

		AED:0.04 eAED:0.04 QI:190|1|1|1|1|1|6|289|477)' 0.4267875
**Cellular respiration.**glycolysis.methylglyoxal degradation. Hydroxy-acyl-glutathione hydrolase (GLX2)'	xvis03_211424-pa	hydroxy-acyl-glutathione hydrolase (GLX2) (original description: augustus-gene-2.30-mRNA-1 protein Name:Similar to GLX2-2 Hydroxyacylglutathione hydrolase cytoplasmic (Arabidopsis thaliana) AED:0.23 eAED:0.23 QI:367|1|1|1|1|0.83|6|257|190)' 1.663941
**protein modification.**phosphorylation CAMK protein kinase superfamily.SNF1-related protein kinase (SnRK2)'	xvis03_216515-pa	SNF1-related protein kinase (SnRK2) (original description: augustus-gene-8.70-mRNA-1 protein Name:Similar to SAPK7 Serine/threonine-protein kinase SAPK7 (Oryza sativa subsp. japonica) AED:0.12 eAED:0.12 QI:115|0.83|1|1|1|1|7|226|305)' 1.8659521
**protein modification.**S-glutathionylation.glutaredoxin'	xvis03_218158-pa	glutaredoxin (original description: augustus-gene-3.24-mRNA-1 protein Name:Similar to Glutaredoxin (Ricinus communis) AED:0.01 eAED:0.01 QI:220|1|1|1|1|1|4|501|106)' 1.1671811
**protein modification**.protein folding.protein folding catalyst (Cyclophilin)'	xvis03_206458-pa	protein folding catalyst (original description: processed-gene-30.78-mRNA-1 protein Name:Similar to PCKR1 Peptidyl-prolyl cis-trans isomerase (Catharanthus roseus) AED:0.01 eAED:0.01 QI:0|-1|0|1|-1|1|1|0|173)' 0.88642055
**protein modification.**protein folding.protein folding catalyst (Cyclophilin)'	xvis03_223454-pa	protein folding catalyst (original description: processed-gene-3.78-mRNA-1 protein Name:Similar to PCKR1 Peptidyl-prolyl cis-trans

		isomerase (Catharanthus roseus) AED:0.02 eAED:0.02 QI:0|-1|0|1|-1|1|1|0|173)' 1.266422
**protein translocation**.chloroplast.outer envelope insertion system.chaperone (AKR2)	xvis03_217196-pa	chaperone (AKR2) (original description: augustus-gene-1.26-mRNA-1 protein Name:Similar to AKR2 Ankyrin repeat domain-containing protein 2 (Arabidopsis thaliana) AED:0.11 eAED:0.11 QI:0|0|0|1|1|1|6|0|276)' 1.775295
**protein translocation.**nucleus.nucleocytoplasmic transport.importin alpha adaptor proteins'	xvis03_215434-pa	importin alpha adaptor proteins (original description: augustus-gene-6.51-mRNA-1 protein Name:Similar to Os01g0253300 Importin subunit alpha-1a (Oryza sativa subsp. japonica) AED:0.21 eAED:0.21 QI:191|0.81|0.75|1|1|1|12|0|614)' 2.079471
**protein translocation**.nucleus.nucleocytoplasmic transport.GTpase activation accessory protein (RanBp1)	xvis03_204022-pa	GTPase activation accessory protein (RanBP1) (original description: processed-gene-29.69-mRNA-1 protein Name:Similar to RANBP1C Ran-binding protein 1 homolog c (Arabidopsis thaliana) AED:0.01 eAED:0.01 QI:85|1|1|1|1|1|4|338|224)' 1.8266815
**RNA processing.**RNA chaperone activities.RNA chaperone (CSp)'	xvis03_206453-pa	RNA chaperone (CSP) (original description: processed-gene-29.128-mRNA-1 protein Name:Similar to CSP4 Cold shock domain-containing protein 4 (Arabidopsis thaliana) AED:0.32 eAED:0.32 QI:0|-1|0|1|-1|1|1|0|231)' 1.2821975
**RNA processing.**RNA chaperone activities.RNA chaperone (CSp)'	xvis03_223451-pa	RNA chaperone (CSP) (original description: processed-gene-3.71-mRNA-1 protein Name:Similar to

		CSP1 Cold shock protein 1 (Arabidopsis thaliana) AED:0.30 eAED:0.30 QI:0|-1|0|1|-1|1|1|0|215)' 2.1634965
**RNA processing.**RNA chaperone activities.RNA chaperone (RZ1|GR-RBp)'	xvis03_215153-pa	RNA chaperone (RZ1|GR-RBP) (original description: augustus-gene-12.40-mRNA-1 protein Name:Similar to RZ1B Glycine-rich RNA-binding protein RZ1B (Arabidopsis thaliana) AED:0.25 eAED:0.25 QI:0|1|1|1|0.5|0.33|3|1599|326)' 1.83855
**RNA processing**.organelle machinery.RNA editing.RNA editing factor (MORF-type)'	xvis03_221754-pa	RNA editing factor (MORF) (original description: augustus-gene-1.62-mRNA-1 protein Name:Similar to MORF1 Multiple organellar RNA editing factor 1, mitochondrial (Arabidopsis thaliana) AED:0.23 eAED:0.23 QI:47|1|1|1|0.75|0.6|5|384|406)' 1.7289507
**External stimuli response.** Temperature. Heat response.organellar chaperone (Clp-p|Clp-m)'	xvis03_207210-pa	organellar chaperone (Clp-p|Clp-m) (original description: augustus-gene-28.48-mRNA-1 protein Name:Similar to CLPB3 Chaperone protein ClpB3, mitochondrial (Oryza sativa subsp. japonica) AED:0.12 eAED:0.12 QI:139|1|1|1|1|1|10|182|968)' 1.7187872
**External stimuli response.**temperature.cold response.mRNA chaperone (CSD)'	xvis03_206453-pa	mRNA chaperone (CSD) (original description: processed-gene-29.128-mRNA-1 protein Name:Similar to CSP4 Cold shock domain-containing protein 4 (Arabidopsis thaliana) AED:0.32 eAED:0.32 QI:0|-1|0|1|-1|1|1|0|231)' 1.2821975

**External stimuli response.**temperature.cold response.mRNA chaperone (CSD)'	xvis03_223451-pa	mRNA chaperone (CSD) (original description: processed-gene-3.71-mRNA-1 protein Name:Similar to CSP1 Cold shock protein 1 (Arabidopsis thaliana) AED:0.30 eAED:0.30 QI:0|-1|0|1|-1|1|1|0|215)' 2.1634965
**Lipid metabolism**.glycerolipid biosynthesis.phosphatidylcholine.CTp:phosphorylcholine cytidylyltransferase'	xvis03_202655-pa	CTP:phosphorylcholine cytidylyltransferase (original description: processed-gene-53.13-mRNA-1 protein Name:Similar to CCT2 Choline-phosphate cytidylyltransferase 2 (Arabidopsis thaliana) AED:0.15 eAED:0.15 QI:50|1|1|1|1|1|8|392|291)' 2.4340105
**Lipid metabolism.**lipid degradation.phospholipase activities.phospholipase A2 activities.phospholipase A2 (ppLA2-II)'	xvis03_218200-pa	phospholipase A2 (pPLA2-II) (original description: augustus-gene-6.42-mRNA-1 protein Name:Similar to PLP1 Patatin-like protein 1 (Oryza sativa subsp. japonica) AED:0.26 eAED:0.26 QI:72|0.4|0.16|1|1|1|6|0|314)' 1.306471
**Lipid metabolism.**lipid degradation.fatty acid degradation.core beta-oxidation.3-ketoacyl-CoA thiolase (KAT)'	xvis03_216330-pa	3-ketoacyl-CoA thiolase (KAT) (original description: processed-gene-6.102-mRNA-1 protein Name:Similar to PED1 3-ketoacyl-CoA thiolase 2, peroxisomal (Arabidopsis thaliana) AED:0.14 eAED:0.14 QI:702|0.85|0.93|0.93|1|1|15|1904|479)' 2.3141983
**Lipid metabolism.**lipid bodies-associated activities.caleosin lipid body surface protein (CLO/pXG)'	xvis03_221236-pa	caleosin (original description: augustus-gene-4.54-mRNA-1 protein Name:Similar to PXG Peroxygenase (Oryza sativa subsp. japonica) AED:0.38 eAED:0.38

		QI:85|0.66|0.85|0.85|1|1|7|299|316)' 1.63025

Table S15: List of the Bin names, id and description of the downregulated proteins at LRD

BinName	id	Description
**photosynthesis**.photophosphorylation.photosystem II.LHC-II complex.component LHCb1/2/3'	xvis03_219722-pa	component LHCb1/2/3 of LHC-II complex (original description: augustus-gene-9.73-mRNA-1 protein Name:Similar to CAB36 Chlorophyll a-b binding protein 36, chloroplastic (Nicotiana tabacum) AED:0.36 eAED:0.36 QI:240|1|1|1|1|1|2|393|264)'
**photosynthesis**.photophosphorylation.photosystem II.LHC-II complex.component LHCb1/2/3'	xvis03_223077-pa	component LHCb1/2/3 of LHC-II complex (original description: processed-gene-1.89-mRNA-1 protein Name:Similar to CAB13 Chlorophyll a-b binding protein 13, chloroplastic (Solanum lycopersicum) AED:0.07 eAED:0.07 QI:182|1|1|1|1|1|4|188|263)'
**photosynthesis**.photophosphorylation.photosystem II.LHC-II complex.component LHCb1/2/3'	xvis03_224654-pa	component LHCb1/2/3 of LHC-II complex (original description: processed-gene-1.9-mRNA-1 protein Name:Similar to CAB21 Chlorophyll a-b binding protein 21, chloroplastic (Nicotiana tabacum) AED:0.11 eAED:0.11 QI:0|0|0|0.5|1|1|2|0|328)'
**photosynthesis.**photophosphorylation.photosystem II.LHC-II complex.component LHCb4'	xvis03_212711-pa	component LHCb4 of LHC-II complex (original description: processed-gene-16.130-mRNA-1 protein Name:Similar to LHCB4.2 Chlorophyll a-b binding protein CP29.2, chloroplastic (Arabidopsis thaliana) AED:0.05 eAED:0.05 QI:1875|1|1|1|0.5|0.33|3|204|289)'
**photosynthesis.**photophosphorylation.photosystem II.LHC-II complex.component LHCb5'	xvis03_202336-pa	component LHCb5 of LHC-II complex (original description: augustus-gene-27.98-mRNA-1 protein Name:Similar to LHCB5 Chlorophyll a-b binding protein CP26, chloroplastic (Arabidopsis thaliana)

		AED:0.08 eAED:0.08 QI:26|1|1|1|1|1|6|330|363)'
**photosynthesis**.photophosphorylation.photosystem II.LHC-II complex.component LHCb6'	xvis03_217301-pa	component LHCb6 of LHC-II complex (original description: augustus-gene-11.85-mRNA-1 protein Name:Similar to CAP10A Chlorophyll a-b binding protein CP24 10A, chloroplastic (Solanum lycopersicum) AED:0.00 eAED:0.00 QI:77|1|1|1|1|1|2|315|254)'
**photosynthesis.**photophosphorylation.photosystem II.pS-II complex.oxygen-evolving center (OEC) extrinsic proteins.component OEC33/psbO'	xvis03_219859-pa	component PsbO/OEC33 of PS-II oxygen-evolving center (original description: augustus-gene-3.73-mRNA-1 protein Name:Similar to PSBO Oxygen-evolving enhancer protein 1, chloroplastic (Solanum lycopersicum) AED:0.03 eAED:0.03 QI:216|1|1|1|1|1|2|224|332)'
**photosynthesis**.photophosphorylation.photosystem I.LHC-I complex.component LHCa1'	xvis03_225147-pa	component LHCa1 of LHC-I complex (original description: augustus-gene-0.41-mRNA-1 protein Name:Similar to CAB6A Chlorophyll a-b binding protein 6A, chloroplastic (Solanum lycopersicum) AED:0.00 eAED:0.00 QI:106|1|1|1|1|1|3|75|204)'
**photosynthesis**.photophosphorylation.photosystem I.LHC-I complex.component LHCa1'	xvis03_225829-pa	component LHCa1 of LHC-I complex (original description: augustus-gene-0.17-mRNA-1 protein Name:Similar to LHCA1 Chlorophyll a-b binding protein 6, chloroplastic (Arabidopsis thaliana) AED:0.45 eAED:0.45 QI:0|0.44|0.4|0.5|1|1|10|334|459)'
**photosynthesis.**photophosphorylation.photosystem I.LHC-I complex.component LHCa2'	xvis03_205558-pa	component LHCa2 of LHC-I complex (original description: augustus-gene-14.7-mRNA-1 protein Name:Similar to CAB7 Chlorophyll a-b binding protein 7, chloroplastic (Solanum lycopersicum) AED:0.16 eAED:0.16 QI:0|0.25|0.4|1|0.75|0.8|5|892|278)'

**photosynthesis.**photophosphorylation.photosystem I.pS-I complex.component psaD'	xvis03_207379-pa	component PsaD of PS-I complex (original description: processed-gene-13.65-mRNA-1 protein Name:Similar to psaD Photosystem I reaction center subunit II, chloroplastic (Cucumis sativus) AED:0.32 eAED:0.32 QI:0|-1|0|1|-1|1|1|0|274)'
**photosynthesis.**photophosphorylation.photosystem I.pS-I complex.component psaF'	xvis03_216014-pa	component PsaF of PS-I complex (original description: processed-gene-5.37-mRNA-1 protein Name:Similar to PSAF Photosystem I reaction center subunit III, chloroplastic (Arabidopsis thaliana) AED:0.24 eAED:0.24 QI:0|-1|0|1|-1|1|1|0|232)'
**photosynthesis.**photophosphorylation.photosystem I.pS-I complex.component psaH'	xvis03_205319-pa	component PsaH of PS-I complex (original description: processed-gene-30.54-mRNA-1 protein Name:Similar to PSAH1 Photosystem I reaction center subunit VI-1, chloroplastic (Arabidopsis thaliana) AED:0.09 eAED:0.09 QI:15|1|1|1|1|1|3|336|145)'
**photosynthesis**.photophosphorylation.photosystem I.pS-I complex.component psaL'	xvis03_200779-pa	component PsaL of PS-I complex (original description: processed-gene-89.11-mRNA-1 protein Name:Similar to PSAL Photosystem I reaction center subunit XI, chloroplastic (Spinacia oleracea) AED:0.06 eAED:0.06 QI:217|1|1|1|1|1|2|190|220)'
**photosynthesis.**calvin cycle.transketolase'	xvis03_207774-pa	transketolase (original description: processed-gene-21.50-mRNA-1 protein Name:Similar to Transketolase, chloroplastic (Solanum tuberosum) AED:0.17 eAED:0.17 QI:154|0.85|0.75|1|1|1|8|0|716)'
**photosynthesis**.calvin cycle.transketolase'	xvis03_219001-pa	transketolase (original description: processed-gene-2.79-mRNA-1 protein Name:Similar to Transketolase, chloroplastic (Solanum tuberosum) AED:0.04 eAED:0.04 QI:238|1|1|1|1|1|7|473|742)'
**Carbohydrate metabolism.**starch metabolism.biosynthesis.ADp-glucose pyrophosphorylase'	xvis03_220998-pa	ADP-glucose pyrophosphorylase (original description: augustus-gene-7.121-mRNA-1 protein Name:Similar to ADG2 Glucose-1-phosphate adenylyltransferase large subunit

		1, chloroplastic (Arabidopsis thaliana) AED:0.11 eAED:0.11 QI:196|0.92|0.86|1|0.85|0.86|15|136|540)'
**Carbohydrate metabolism**.starch metabolism.biosynthesis.starch synthase activities.granule-bound starch (amylose) synthase	xvis03_225238-pa	granule-bound starch (amylose) synthase (original description: augustus-gene-0.18-mRNA-1 protein Name:Similar to WAXY Granule-bound starch synthase 1, chloroplastic/amyloplastic (Antirrhinum majus) AED:0.11 eAED:0.11 QI:73|1|1|1|0.92|0.85|14|142|611)'
**Carbohydrate metabolism**.oxidative pentose phosphate pathway.non-oxidative phase.transketolase'	xvis03_207774-pa	transketolase (original description: processed-gene-21.50-mRNA-1 protein Name:Similar to Transketolase, chloroplastic (Solanum tuberosum) AED:0.17 eAED:0.17 QI:154|0.85|0.75|1|1|1|8|0|716)'
**Carbohydrate metabolism.**oxidative pentose phosphate pathway.non-oxidative phase.transketolase'	xvis03_219001-pa	transketolase (original description: processed-gene-2.79-mRNA-1 protein Name:Similar to Transketolase, chloroplastic (Solanum tuberosum) AED:0.04 eAED:0.04 QI:238|1|1|1|1|1|7|473|742)'
**Carbohydrate metabolism.**gluconeogenesis.pyruvate orthophosphate dikinase activity.pyruvate orthophosphate dikinase'	xvis03_201017-pa	pyruvate orthophosphate dikinase (original description: processed-gene-109.26-mRNA-1 protein Name:Similar to PPD Pyruvate, phosphate dikinase, chloroplastic (Mesembryanthemum crystallinum) AED:0.09 eAED:0.09 QI:8|1|1|1|0.89|0.9|20|257|985)'
**Carbohydrate metabolism.**nucleotide sugar biosynthesis.UDp-L-arabinose biosynthesis.UDp-L-arabinose mutase'	xvis03_224372-pa	UDP-L-arabinose mutase (original description: processed-gene-2.16-mRNA-1 protein Name:Similar to UAM1 UDP-arabinopyranose mutase 1 (Oryza sativa subsp. japonica) AED:0.08 eAED:0.08 QI:87|1|1|1|1|1|4|347|359)'
**protein homeostasis**.ubiquitin-proteasome system.26S proteasome.20S core particle.beta-type components.component beta type-5'	xvis03_211689-pa	component beta type-5 of 26S proteasome (original description: processed-gene-6.93-mRNA-1 protein Name:Similar to Proteasome subunit beta type-5 (Spinacia

		oleracea) AED:0.07 eAED:0.07 QI:0|1|0.87|1|1|1|8|489|310)'
**protein homeostasis**.proteolysis.cysteine-type peptidase activities.C13-class asparaginyl endopeptidase (Legumain)'	xvis03_211268-pa	asparaginyl endopeptidase (Legumain) (original description: processed-gene-9.25-mRNA-1 protein Name:Similar to Vacuolar-processing enzyme (Citrus sinensis) AED:0.07 eAED:0.07 QI:3|1|1|1|1|1|9|417|590)'
**protein homeostasis**.proteolysis.serine-type peptidase activities.S28-class serine carboxypeptidase'	xvis03_222506-pa	S28-class serine carboxypeptidase (original description: augustus-gene-4.97-mRNA-1 protein Name:Similar to EDA2 Probable serine protease EDA2 (Arabidopsis thaliana) AED:0.31 eAED:0.31 QI:159|0.92|0.92|1|1|1|14|279|488)'
**Protein homeostasis**.proteolysis.metallopeptidase activities.aminopeptidase activities.M1-class neutral/aromatic-hydroxyl amino acid aminopeptidase'	xvis03_210782-pa	M1 neutral/aromatic-hydroxyl amino acid aminopeptidase (original description: augustus-gene-7.64-mRNA-1 protein Name:Similar to Os08g0562700 Puromycin-sensitive aminopeptidase (Oryza sativa subsp. japonica) AED:0.20 eAED:0.20 QI:160|0.75|0.81|0.90|0.84|0.84|33|284|1052)'
**Redox homeostasis**.hydrogen peroxide removal.ascorbate-glutathione cycle.ascorbate peroxidase (ApX)'	xvis03_214214-pa	ascorbate peroxidase (APX) (original description: processed-gene-11.33-mRNA-1 protein Name:Similar to APX1 L-ascorbate peroxidase, cytosolic (Pisum sativum) AED:0.17 eAED:0.17 QI:131|1|1|1|1|1|7|558|249)'
**Redox homeostasis.**hydrogen peroxide removal.ascorbate-glutathione cycle.monodehydroascorbate reductase (MDAR)'	xvis03_204322-pa	monodehydroascorbate reductase (MDAR) (original description: processed-gene-9.15-mRNA-1 protein Name:Similar to AFRR Monodehydroascorbate reductase (Solanum lycopersicum) AED:0.13 eAED:0.13 QI:138|0.72|0.83|0.83|1|1|12|368|489)'
**Redox homeostasis.**hydrogen peroxide removal.ascorbate-glutathione cycle.glutathione reductase (GR)'	xvis03_205102-pa	glutathione reductase (GR) (original description: processed-gene-9.15-mRNA-1 protein Name:Similar to GOR Glutathione reductase, chloroplastic (Fragment)

		(Nicotiana tabacum) AED:0.14 eAED:0.14 QI:0|1|0.90|1|0.9|0.81|11|639|590)'
**Cell wall** organisation.pectin.homogalacturonan.modification and degradation.pectin methylesterase'	xvis03_217074-pa	pectin methylesterase (original description: processed-gene-2.49-mRNA-1 protein Name:Similar to PME35 Probable pectinesterase/pectinesterase inhibitor 35 (Arabidopsis thaliana) AED:0.36 eAED:0.36 QI:662|1|1|1|1|1|3|218|442)'
**Cell wall** organisation.pectin.rhamnogalacturonan I.modification and degradation.beta-galactosidase (BGAL)'	xvis03_221434-pa	beta-galactosidase (BGAL) (original description: processed-gene-2.106-mRNA-1 protein Name:Similar to Beta-galactosidase (Asparagus officinalis) AED:0.13 eAED:0.13 QI:277|1|1|1|1|1|19|418|832)'
**Coenzyme metabolism**.thiamine pyrophosphate biosynthesis.thiazole biosynthesis.biosynthetic protein (Thi4)'	xvis03_212280-pa	biosynthetic protein (Thi4) (original description: processed-gene-6.79-mRNA-1 protein Name:Similar to THI1-2 Thiamine thiazole synthase 2, chloroplastic (Vitis vinifera) AED:0.02 eAED:0.02 QI:212|1|1|1|1|1|2|483|354)'
**Coenzyme metabolism.**tetrapyrrol biosynthesis.chlorophyll metabolism.chlorophyll(ide) interconversions.geranylgeranyl reductase (Chlp)'	xvis03_215774-pa	geranylgeranyl reductase (ChlP) (original description: augustus-gene-7.23-mRNA-1 protein Name:Similar to CHLP Geranylgeranyl diphosphate reductase, chloroplastic (Nicotiana tabacum) AED:0.00 eAED:0.00 QI:382|1|1|1|1|1|2|212|364)'
**protein biosynthesis**.aminoacyl-tRNA synthetase activities.alanine-tRNA ligase'	xvis03_224672-pa	alanine-tRNA ligase (original description: processed-gene-1.89-mRNA-1 protein Name:Similar to ALATS Alanine--tRNA ligase (Arabidopsis thaliana) AED:0.10 eAED:0.10 QI:33|1|1|1|0.90|0.90|22|389|993)'
**protein biosynthesis**.aminoacyl-tRNA synthetase activities.aspartate-tRNA ligase'	xvis03_205724-pa	aspartate-tRNA ligase (original description: processed-gene-33.32-mRNA-1 protein Name:Similar to IBI1 Aspartate--tRNA ligase 2, cytoplasmic (Arabidopsis thaliana) AED:0.10 eAED:0.10 QI:60|0.77|0.8|1|1|1|10|190|552)'
